# Downstream community risks post dam-spillage flooding along the Volta River in Ghana: potential pathogens and public health implications

**DOI:** 10.1371/journal.pone.0346766

**Published:** 2026-07-02

**Authors:** Grace Semabia Kpeli, Prince Agyirey-Kwakye, Counseller Nutifafa Livingstone, Lillian Teye Cudjoe, Emmanuel Edem Dotse, Ebenezer Nyarko, Ebenezer Zar, Obed Nyarko-Otu, Hubert Kwame Agbogli, Godwin Glilekpeh, Solomon Korankye, Priscilla Essandoh, Daniel Elorm Kabotso

**Affiliations:** 1 Department of Biomedical Science, University of Health and Allied Sciences, Sokode Lokoe, Ho, Ghana; 2 Department of Clinical Genomics, AttoDiagnostics Ltd, Attogroup, Norwich, Norfolk, United Kingdom; 3 Department of Basic Science, University of Health and Allied Sciences, Sokode Lokoe, Ho, Ghana; Universidade Estadual de Ponta Grossa, BRAZIL

## Abstract

Flooding compromises the microbiological quality of domestic and recreational water sources, increasing exposure to waterborne and opportunistic pathogens in affected communities. Following widespread flooding triggered by the September 2023 controlled spillage of the Akosombo and Kpong dams in Ghana, which led to massive flooding, we assessed the microbial quality of water bodies in communities downstream of the Volta River and associated public and ecosystem health implications. In July 2024, water was collected from multiple source types across four downstream townships (Asutsuare, Aveyime, Battor, and Mepe), including river sections, ponds, puddles, wells, boreholes, and canal-associated sites. Samples were filtered, microbial DNA was extracted, and bacterial community composition was profiled using 16S rRNA gene sequencing, with downstream bioinformatics analyses used to characterize microbial diversity, assess spatial variation across sites, and stratify detected taxa according to ecological origin and public health risk. Sequence data obtained from 17 out of 22 samples revealed communities dominated by Proteobacteria with frequent co-occurrence of Bacteroidetes and Firmicutes. Across all sites, 56 microbial taxa and 118 microorganisms were detected. Community composition varied by location and water source type, with bank and mid-river sites generally exhibiting higher richness than puddles and a canal drain. Thirteen samples contained site-specific taxa, indicating marked spatial heterogeneity within and between communities. Microorganisms of public health relevance were frequently detected, including enteric-associated bacteria such as *Escherichia coli, Salmonella enterica, Shigella species*, and *Cronobacter sakazakii*, as well as opportunistic and healthcare-associated bacteria including *Acinetobacter baumannii*, *Klebsiella pneumoniae*, *Pseudomonas aeruginosa*, and *Stenotrophomonas maltophilia*. Higher-risk organisms were most widely distributed in Mepe and Battor, including in groundwater-associated sources. These findings provide molecular evidence of persistent, spatially heterogeneous microbial hazards months after flooding, underscoring continued exposure risks in downstream communities and the need for targeted water safety interventions and risk-informed public health responses.

## Introduction

Events that occur post-flooding are a major concern for public health, stemming from the fact that there is the drastic deterioration of drinking and recreational water quality due to increased microbial contamination ([1,2]). Floodwaters act as vectors, transporting a diverse range of microbial pathogens and fecal contaminants from various sources, including agriculture, industrial effluents, human and animal waste, into receiving rivers and streams. This poses dire, long-lasting health challenges to affected communities ([3,4]). An increase in microbial contamination implies an increase in the risk of acquiring waterborne diseases such as dysentery, typhoid fever, leptospirosis, diarrhea etc. which may be life-threatening especially for the vulnerable (children, elderly individuals, immunocompromised individuals, pregnant women etc.) members of affected communities [5,6]. An additional post-flood concern is that affected individuals are at risk of developing skin, ear, eye and respiratory infections due to the presence of diverse pathogens [7].

These infections, depending on their severity and repeated occurrences, may lead to chronic, life-long organ damage, examples being bladder cancer and liver fibrosis caused by Schistosomiasis infection. In addition to the risk of drowning from rising water levels, infections caused by pathogens found in waterbodies post-flood may lead to increased rates of mortality [8]. Studies have consistently demonstrated a rise in fecal indicator bacteria in flood-affected rivers, emphasizing the increased likelihood of microbial contamination and subsequent health risks [9]. The presence of indicator organisms such as *Escherichia coli*, fecal coliforms, enterococci, *Pseudomonas aeruginosa*, *Clostridium perfringens*, and specific viruses and protozoans, signals potential fecal contamination and the increased risk of pathogenic microorganisms known for causing diseases such as diarrhea, urinary tract infections (UTIs), cholera among many others [10,11]. These contaminants pose significant health threats to communities that depend heavily on affected water sources for survival (domestic purposes) frequently leading to outbreaks of waterborne diseases [12,13].

Beyond the immediate public health concerns, the broader impacts of flooding extend to socio-economic consequences, including reduced agricultural yields and harsh effects on the health-related quality of life for affected populations, manifesting as increased rates of depression, anxiety, and chronic pain that can persist for years [14,15].

The Kpong Dam, a significant hydroelectric facility on Ghana's Volta River, was constructed between 1977 and 1982 and since its construction, has been extremely beneficial to Ghana through the generation of electricity [16–18]. In addition to power generation, the dam serves as a water source for irrigation and municipal water supply [19]. However, its operation poses serious public health challenges for downstream communities, with Bilharzia (Schistosomiasis) endemic in some of these communities since its construction [20–22]. Facing critical water levels due to unusually heavy rainfall in late 2023, the Volta River Authority (VRA) as part of regulatory duties initiated a controlled spillage with increased discharge volumes on September 15^th^ 2023, to avert potential structural failure. This controlled spillage coinciding with heavy rainfalls at the time, resulted in widespread and devastating flooding across downstream communities along the Volta River highly impacting 7 districts of the Volta, Eastern and Greater Accra Regions [23,24]. Towns including Mepe, Sogakope, Battor, Adidome, Mafi, Tefle, Wume and Sokpoe were severely hit, with Mepe experiencing the worst inundation. The spillage triggered a humanitarian crisis, displacing tens of thousands of individuals, leading to significant losses of homes, livelihoods, and critical public health infrastructure [25,26].

Understanding the microbiological quality of water post-flood particularly in downstream communities is necessary for assessing the extent of contamination, identifying potential health threats, and implementing effective risk mitigation strategies and public health interventions. This paper aimed at assessing the potential emergence of pathogenic and potentially pathogenic bacteria post flooding, with implications for human and ecosystem health. We sought to comprehensively investigate the complex and varying effects of the Kpong Dam spillage on public health in the downstream communities of Asutsuare, Aveyime, Battor and Mepe communities using 16S rRNA gene sequencing of water samples from these towns.

## Materials and methods

The study utilized a cross-sectional approach to assess the presence and prevalence of pathogenic and potentially pathogenic bacteria in the downstream communities with implications for human health post-flooding. Water samples were collected from four townships; Asutsuare, Aveyime, Battor, and Mepe, located downstream of the Kpong dam in the North Tongu District of the Volta Region. These communities except for Asutsuare were affected by the controlled spillage from the dam. Sample collection was done in July 2024, when floodwaters had receded, and it was safe to go into the communities. The interval between the September 2023 dam spillage and the July 2024 sampling reflects two sequential phases: an initial sampling round was conducted once floodwaters had receded and it was safe to access the communities, but DNA quality from those initial samples was insufficient for sequencing. A second sampling round was therefore undertaken in July 2024 to obtain samples of adequate quality. Importantly, this extended post-flood window also allowed assessment of the persistence of microbial contamination well after the acute flooding phase. All study procedures, including field site access and sample collection, were conducted under ethical clearance obtained from the Research Ethics Committee of the University of Health and Allied Sciences (UHAS), Ho. Approval was granted for each study community under the following protocol identification numbers: Asutsuare (UHAS-REC A.10 [162] 23–24), Aveyime (UHAS-REC A.10 [245] 23–24), Battor (UHAS-REC A.10 [248] 23–24), and Mepe (UHAS-REC A.10 [175] 23–24). No additional permits were required for field site access, as sampling was conducted from publicly accessible water bodies along the Volta River downstream of the Kpong Dam.

### Sampling design and sample collection

Sampling sites were selected in the field to represent various water sources within the community, including sections of the Volta River, taps, wells, boreholes, ponds, and other local water bodies. The exact locations of each sampling site were geo-referenced using a handheld GPS device, and coordinates were recorded to ensure spatial traceability. GPS coordinates for all 22 sampling sites are provided as supporting information S1 Table. Water samples were collected using the direct immersion grab sampling method. Sterile bottles were immersed below the surface, and the caps were removed only after the bottles were submerged to minimize surface contamination and ensure representative water collection. At each site, 1.5 L of water was collected in sterile containers in accordance with Environmental Protection Agency (EPA) guidelines [27] for water sample collection. Each sample was given a unique identifier and transported on ice, in tightly sealed sterile styrofoam boxes. For quality control, samples were taken in duplicate from each site. A total of 22 samples were obtained from the Volta River, as well as other sources in the townships including wells, domestic taps, ponds, and puddles. Except for the Battor township where 7 samples were collected, 5 samples each were collected in the Asutsuare, Aveyime, and Mepe townships. In the Asutsuare township: 1 sample was obtained from a canal, 1 from the canal drain and 3 from different sections across the river. From Aveyime: 2 samples were obtained from the river, 1 from a pond, and 2 from two separate puddles, on the riverbank. From Battor: 4 samples were obtained from the river, and one each from a pond, borehole, and a domestic groundwater well. From Mepe: 1 sample was obtained from a pond; 3 obtained directly from the river (banks and middle), while one was collected from a household groundwater well. The detailed sampling protocol is available at https://dx.doi.org/10.17504/protocols.io.4r3l2dd64g1y/v2 [28].

### Sample processing

Samples underwent vacuum filtration using a Whatman 0.45µm filter paper (Cytvia, UK). The filter papers were subsequently immersed in 1X phosphate-buffered saline solution to resuspend the cells. DNA was extracted using the QIAamp® DNA Mini Kit (Qiagen N.V., Germany), following the manufacturer’s instructions. The extracted DNA was stored at −20^o^C till use. The concentrations and purities of the extracts were assessed using a NanoDrop^TM^ Spectrophotometer (Thermo Fisher Scientific, U.S.) at the Noguchi Memorial Institute for Medical Research, Accra, Ghana; and Qubit^TM^ dsDNA HS quantification (Thermo Fisher Scientific, Waltham, Massachusetts, U.S.) at AttoDiagnostics Limited, Norwich, UK. The detailed laboratory protocol is available https://dx.doi.org/10.17504/protocols.io.kqdg3mmkpl25/v2 [29].

### 16S rRNA gene sequencing and post-sequencing analyses

16S rRNA gene sequencing was performed for 17 samples which were made up of 2 samples from Asutsuare, 5 from Aveyime, 6 from Battor, and 4 from Mepe. Five other samples were not processed due to low DNA quality. DNA sequencing was done at AttoDiagnostics Limited, Norwich, UK. 16S rRNA amplicons covering 8 out of the 9 hypervariable regions (HV2 to HV9) were amplified using the Ion AmpliSeq Microbiome Health Research Kit, followed by targeted sequencing using the Ion 540 Chip (Cat# 46496, ThermoFisher Scientific, USA) on the Ion GeneStudio™ S5 platform (Thermo Fisher Scientific, U.S.) following the manufacturers protocol. Demultiplexing and quality control processes were performed using Ion Reporter^TM^ Software version 5.18 (Thermo Fisher Scientific, U.S.). Taxonomic assignments were made using the Greengenes database (version 13.5), while alpha diversity metrics were calculated and visualized using the RStudio IDE version 2025.05.0 + 496 (“Mariposa Orchid”) (Posit Software, PBC). The minimum read counts for the different taxonomic levels were set at 500 for the genus and family, and 100 for the species level, while normalization thresholds were established at 0.5 for genus and family levels, and 0.25 for the species. Results obtained after the sequencing and post-sequencing analyses were organized using Microsoft Excel LTSC Standard Version 16.99.1 (25072013). The detailed sequencing protocol is available at: https://dx.doi.org/10.17504/protocols.io.5jyl84468g2w/v1 [30].

### Ecological and risk-based classification of microorganisms

Following 16S rRNA sequencing, microbial isolates were categorized using an ecological and risk-based classification framework into healthcare-associated, environmental/aquatic, enteric and opportunistic organisms. This enabled the grouping of microorganisms by both their likely origin and potential impact on human health. Healthcare-associated microbes included organisms commonly found in healthcare settings and typically associated with nosocomial infections; environmental and aquatic microbes referred to those naturally occurring in soil, water bodies and surrounding ecosystems without direct association with human disease. Enteric organisms were defined as those originating from faecal contamination and typically associated with the gastrointestinal tract of humans and animals while opportunistic pathogens included microbes which are generally harmless in healthy individuals but have the potential to cause infection in the immunocompromised. This classification facilitated the understanding of the microbial diversity present in the samples collected while highlighting potential health risks associated with different microbes.

### Data analysis

All data was organized in Microsoft Excel Version 16.99.1 (25072013) and analyzed using RStudio (IDE version 2025.05.0 + 496 “Mariposa Orchid”; Posit Software, PBC) and the R packages *ggplot2*, *dplyr*, *tidyr*, *readr*, *viridis*, and *pheatmap*. The detailed bioinformatics and risk classification protocol is available at https://dx.doi.org/10.17504/protocols.io.rm7vz44o2lx1/v1 [31].

## Results

### Taxonomic composition and diversity of microbial isolates

From the 17 samples successfully sequenced, a total of 56 distinct microbial taxa and 118 distinct microorganisms were recorded (Table 1). Samples from all 4 towns were consistently dominated by Proteobacteria, whiles Bacteroidetes and Firmicutes also frequently co-occurred across sites. Acinetobacter were detected at lower relative abundances in several samples

**Table 1:** Taxonomic composition of samples.

Town	Sample ID	Total Microbial Taxa Identified	Phylum Level Distribution	Identified Microbial Species	Unique Microbes	Public Health Relevance
ASUTSUARE	ALBQ(Bank)	28	Proteobacteria 89%; Bacteroidetes 4%; Firmicutes 4%; Actinobacteria 3%	*Acinetobacter baumannii,* *Acidovorax* sp.*, Aeromonas hydrophila, Bacillus cereus,* *Burkholderia* sp.*, Citrobacter freundii, Cronobacter sakazakii, Enterobacter aerogenes, Enterobacter cloacae, Enterobacter hormaechei, Enterobacter ludwigii,* *Enterobacter* sp.*, Escherichia coli,* *Flavobacterium* sp.*,* *Iodobacter* sp.*, Klebsiella oxytoca, Klebsiella pneumoniae,* *Legionella* sp.*, Pantoea agglomerans, Propionibacterium acnes, Pseudomonas aeruginosa, Pseudomonas putida, Rickettsia endosymbiont, Salmonella enterica, Serratia marcescens, Shigella flexneri, Shigella sonnei, Stenotrophomonas maltophilia*	*Citrobacter freundii, Enterobacter hormaechei,* *Iodobacter* sp*., Shigella sonnei*	High microbial richness; strong fecal contamination signal (multiple enteric pathogens); several MDR-associated nosocomial organisms detected
	CDQ (Canal Drain)	6	Proteobacteria 67%; Bacteroidetes 33%	*Bacteroides* sp.*,* *Comamonas* sp.*, Comamonas testosteroni,* *Desulfovibrio* sp.*,* *Sphingomonas* sp.*,* *Sphingobacterium* sp.	*Desulfovibrio* sp.	Mostly low-virulence environmental/opportunistic taxa; no major high-risk pathogens
AVEYIME	AV. 1 (Puddle)	7	Proteobacteria 71%; Bacteroidetes 14%; Firmicutes 14%	*Pseudomonas aeruginosa, Escherichia coli, Aeromonas veronii,* *Stenotrophomonas* sp.*,* *Bacillus* sp.*, Burkholderia cepacia,* *Flavobacterium* sp.	None	Mixed fecal and opportunistic signal; moderate public-health concern
	AV. A (Puddle)	4	Proteobacteria 75%; Bacteroidetes 25%	*Acidovorax* sp.*, Chitinimonas taiwanensis, Escherichia coli,* *Flavobacterium* sp.	*Chitinimonas taiwanensis*	Clear fecal contamination signal (*E. coli*) despite small sample size
	AV. B (Bank)	18	Proteobacteria 89%; Bacteroidetes 11%	*Acinetobacter baumannii,* *Acinetobacter* sp.*, Aeromonas veronii,* *Alcaligenes* sp., *Brevundimonas* sp.*, Burkholderia cepacia,* *Burkholderia* sp.*,* *Chryseobacterium* sp.*, Comamonas* sp.*, Comamonas aquatica, Comamonas testosteroni, Flavobacterium* sp.*, Pseudomonas aeruginosa, Pseudomonas putida, Pseudomonas* sp.*, Rickettsia endosymbiont, Sphingomonas* sp.*, Stenotrophomonas maltophilia*	*Brevundimonas* sp.*, Comamonas aquatica*	Cluster of MDR-associated HAIs mixed with aquatic opportunists; elevated clinical relevance
	AV. M (Mid-river)	9	Proteobacteria 78%; Bacteroidetes 22%	*Acidovorax facilis, Acidovorax* sp.*, Alcaligenes* sp.*, Enhydrobacter aerosaccus, Flavobacterium psychrophilum, Flavobacterium* sp.*, Pseudomonas aeruginosa, Pseudomonas putida, Pseudomonas* sp.	*Acidovorax facilis, Enhydrobacter aerosaccus, Flavobacterium psychrophilum*	Environmental water community with notable *P. aeruginosa,* moderate human-health relevance
	AV. P(Pond)	14	Proteobacteria 64%; Bacteroidetes 29%; Firmicutes 7%	*Acidovorax* sp.*, Aeromonas hydrophila, Aeromonas veronii, Alcaligenes* sp.*, Bacillus* sp.*, Chryseobacterium* sp.*, Flavobacterium columnare, Flavobacterium* sp.*, Flavisolibacter* sp.*, Pseudomonas aeruginosa, Pseudomonas putida, Pseudomonas* sp.*, Sphingomonas* sp*., Stenotrophomonas maltophilia*	*Flavobacterium columnare, Flavisolibacter* sp.	Freshwater / fish-associated taxa common; presence of opportunistic pathogens
BATTOR	BB(Borehole)	21	Proteobacteria 48%; Bacteroidetes 24%; Firmicutes 19%; Actinobacteria 10%	*Acinetobacter baumannii, Acinetobacter johnsonii, Acinetobacter* sp.*, Aeromonas* sp.*, Bacillus* sp.*, Bacteroides* sp.*, Burkholderia cepacia, Chryseobacterium* sp*., Clostridium* sp.*, Escherichia coli, Flavobacterium* sp.*, Klebsiella* sp*., Mycobacterium* sp.*, Porphyromonas* sp.*, Propionibacterium* sp*., Pseudomonas aeruginosa, Pseudomonas* sp.*, Sphingobacterium* sp.*, Staphylococcus* sp.*, Stenotrophomonas* sp.*, Streptococcus* sp.	*Clostridium* sp.*, Porphyromonas* sp.*, Propionibacterium* sp.*, Staphylococcus* sp.*, Streptococcus* sp.	Highest diversity in Battor; mix of oral/anaerobic, environmental and opportunistic species; broad opportunistic infection potential
	BP(Pond)	12	Proteobacteria 67%; Bacteroidetes 17%; Firmicutes 8%; Actinobacteria 8%	*Acinetobacter baumannii, Acinetobacter* sp.*, Aeromonas* sp.*, Alcaligenes faecalis, Bacillus* sp.*, Burkholderia* sp.*, Flavobacterium* sp.*, Legionella* sp.*, Mycobacterium* sp.*, Pseudomonas* sp.*, Sphingobacterium* sp.*, Stenotrophomonas maltophilia*	*Alcaligenes faecalis*	Combined HAI-Aquatic signature; notable respiratory-associated pathogens
	BVRHq (*Volta River along Battor Holy Quarters***)**	11	Proteobacteria 45%; Bacteroidetes 36%; Firmicutes 9%; Actinobacteria 9%	*Acinetobacter baumannii, Acinetobacter* sp.*, Aeromonas veronii, Bacillus* sp.*, Chryseobacterium* sp.*, Flavobacterium* sp.*, Flavobacterium chungnamense, Flavobacterium macrobrachii, Propionibacterium acnes, Pseudomonas putida, Stenotrophomonas maltophilia*	*Flavobacterium chungnamense, Flavobacterium macrobrachii*	Aquatic/soil organisms common; moderate clinical relevance with some opportunists
	BVRm (Mid-river)	12	Proteobacteria 92%; Bacteroidetes 8%	*Acinetobacter junii, Acinetobacter nosocomialis, Acinetobacter baumannii, Acinetobacter calcoaceticus, Acinetobacter* sp.*, Aeromonas hydrophila, Aeromonas veronii, Aeromonas* sp.*, Escherichia coli, Flavobacterium* sp.*, Pseudomonas* sp.*, Stenotrophomonas* sp.	*Acinetobacter junii, Acinetobacter nosocomialis*	Moderate microbial load; mix of opportunistic and enteric organisms; some MDR indicators
	BVRo (Bank)	20	Proteobacteria 70%; Bacteroidetes 15%; Firmicutes 10%; Actinobacteria 5%	*Acinetobacter johnsonii, Acinetobacter baumannii, Acinetobacter calcoaceticus, Acinetobacter* sp.*, Aeromonas hydrophila, Aeromonas veronii, Bacillus cereus, Bacillus* sp.*, Bacteroides* sp.*, Burkholderia* sp.*, Burkholderia cepacia, Chryseobacterium* sp.*, Escherichia coli, Flavobacterium* sp.*, Klebsiella pneumoniae, Legionella sp., Propionibacterium acnes, Pseudomonas putida, Pseudomonas* sp.*, Stenotrophomonas maltophilia*	None	High complexity: strong healthcare-associated signature plus aquatic *Legionella;* elevated risk
	BW(well)	15	Proteobacteria 67%; Bacteroidetes 27%; Firmicutes 7%	*Aeromonas hydrophila, Aeromonas veronii, Acinetobacter baumannii, Bacillus* sp.*, Chryseobacterium anthropi, Chryseobacterium hominis, Chryseobacterium* sp.*, Escherichia coli, Flavobacterium* sp.*, Pseudomonas alcaligenes, Pseudomonas aeruginosa, Pseudomonas putida, Pseudomonas* sp.*, Pseudomonas stutzeri, Stenotrophomonas maltophilia*	*Chryseobacterium anthropi, Chryseobacterium hominis, Pseudomonas alcaligenes, Pseudomonas stutzeri*	High load of opportunistic Gram-negative rods; several *Chryseobacterium* sp. present
MEPE	M. Bank (Bank)	23	Proteobacteria 74%; Bacteroidetes 13%; Actinobacteria 9%; Firmicutes 4%	*Aeromonas hydrophila, Acinetobacter johnsonii, Acinetobacter tjernbergiae, Acinetobacter radioresistens, Acinetobacter baumannii, Acinetobacter calcoaceticus, Acinetobacter* sp.*, Acinetobacter lwoffii, Acinetobacter beijerinckii, Bacillus* sp.*, Bdellovibrio bacteriovorus, Burkholderia cepacia, Burkholderia* sp.*, Chryseobacterium* sp.*, Flavobacterium* sp.*, Legionella* sp.*, Pseudomonas putida, Pseudomonas* sp.*, Rhodococcus erythropolis, Rhodococcus* sp.*, Sphingobacterium* sp.*, Stenotrophomonas maltophilia, Thermomonas haemolytica*	*Bdellovibrio bacteriovorus*	Highest richness overall; diverse Acinetobacter population and mix of environmental and HAI-associated taxa
	M. Pond (Pond)	22	Proteobacteria 100%	*Acinetobacter baumannii, Aeromonas hydrophila, Aeromonas veronii, Cronobacter sakazakii, Enterobacter aerogenes, Enterobacter asburiae, Enterobacter cloacae, Enterobacter ludwigii, Enterobacter* sp.*, Escherichia coli, Klebsiella oxytoca, Klebsiella* sp.*, Pantoea agglomerans, Pectobacterium carotovorum, Pseudomonas aeruginosa, Salmonella enterica, Serratia marcescens, Serratia* sp.*, Shigella boydii, Shigella flexneri, Stenotrophomonas maltophilia, Xanthomonas* sp.	*Enterobacter asburiae, Pectobacterium carotovorum, Serratia* sp.*, Shigella boydii, Xanthomonas* sp.	Severe fecal contamination signal; many clinically significant enteric pathogens, high public-health concern
	T. Bank (Bank)	4	Proteobacteria 75%; Bacteroidetes 25%	*Aeromonas hydrophila, Acinetobacter* sp.*, Flavobacterium* sp.*, Stenotrophomonas maltophilia*	None	Low-diversity sample dominated by opportunistic species
	T. Middle (Mid-river)	21	Proteobacteria 71%; Bacteroidetes 14%; Actinobacteria 10%; Firmicutes 5%	*Acinetobacter johnsonii, Acinetobacter tjernbergiae, Acinetobacter radioresistens, Acinetobacter baumannii, Acinetobacter calcoaceticus, Acinetobacter* sp.*, Acinetobacter lwoffii, Acinetobacter beijerinckii, Bacillus* sp.*, Burkholderia* sp.*, Burkholderia cepacia, Chryseobacterium* sp.*, Flavobacterium* sp.*, Legionella* sp.*, Pseudomonas putida, Pseudomonas* sp.*, Rhodococcus* sp.*, Rhodococcus erythropolis, Sphingobacterium* sp.*, Stenotrophomonas maltophilia, Thermomonas haemolytica*	None	Extensive *Acinetobacter* representation; strong HAI signature with mixed opportunistic load

At the town level, samples from Asutsuare comprised 34 microbial taxa with the bank sample (ALBQ) showing the highest richness (28 taxa) and a strong dominance of Proteobacteria (89%). Multiple enteric and opportunistic organisms such as *Escherichia coli, Salmonella enterica, Shigella flexneri, Shigella sonnei*, *Acinetobacter baumannii, Klebsiella pneumoniae, Pseudomonas aeruginosa, and Stenotrophomonas maltophilia* were detected, whereas the canal drain sample (CDQ) exhibited lower diversity (6 taxa) and was largely composed of environmental and opportunistic organisms including *Bacteroides* sp., *Comamonas testosteroni*, *Sphingomonas* sp., and *Desulfovibrio* sp.

In Aveyime, a total of 31 taxa were identified across five sampling sites. Proteobacteria remained the dominant phylum in all the samples (64–89%), with variable representation of Bacteroidetes and Firmicutes. The bank and mid-river samples showed higher taxonomic richness compared with puddle samples, with several opportunistic Gram-negative species repeatedly detected. Detected organisms included environmental and opportunistic Gram-negative bacteria such as *Pseudomonas putida*, *Flavobacterium* sp., *Acidovorax* sp., and *Comamonas aquatica*, as well as potentially pathogenic taxa including *Escherichia coli*, *Aeromonas hydrophila*, *Aeromonas veronii*, and *Burkholderia cepacia*.

Samples from Battor showed the highest overall diversity, with 38 microbial taxa identified. Phylum-level distributions were more heterogeneous, with notable contributions from Firmicutes and Actinobacteria alongside Proteobacteria. Detected taxa included opportunistic Gram-negative organisms such as *Acinetobacter baumannii*, *Pseudomonas aeruginosa*, and *Stenotrophomonas maltophilia*, as well as Gram-positive and anaerobic taxa including *Bacillus* sp., *Clostridium* sp., *Staphylococcus* sp., and *Streptococcus* sp. Borehole and bank samples showed broad taxonomic representation, including anaerobic, environmental, and opportunistic organisms compared with the pond and mid-river samples.

In Mepe, 36 taxa were identified, with substantial variation across sites. The pond sample demonstrated complete dominance of Proteobacteria (100%) and included multiple enteric species such as *Escherichia coli*, *Salmonella enterica*, *Shigella boydii*, *Shigella flexneri*, and *Klebsiella* sp, while the bank and mid-river samples showed higher overall richness and broader phylum representation. Several samples displayed extensive representation of *Acinetobacter* species (*A. baumannii*, *A. lwoffii*, *A. calcoaceticus*) alongside other opportunistic taxa such as *Pseudomonas* sp., *Legionella* sp., and *Stenotrophomonas maltophilia* (Table 1).

All 17 successfully sequenced samples displayed distinct microbial profiles. Of these, 13 samples contained microbial taxa that were not detected at any other sampling site indicating the presence of site-specific microorganisms. These unique taxa were distributed across all townships and multiple sample types, including bank, pond, mid-river, canal drain, borehole, and well samples. Several sampling sites were characterized by single unique taxa, such as Desulfovibrio sp. in the canal drain sample (CDQ), *Chitinimonas taiwanensis* in AV. A, *Alcaligenes faecalis* in BP, and *Bdellovibrio bacteriovorus* in the Mepe bank sample. Other sites recorded multiple unique microorganisms, including ALBQ, BB, BW, and M. Pond, each of which harbored between two and five taxa not identified elsewhere. The unique microorganisms identified spanned a range of ecological groups, including enteric bacteria (e.g., *Shigella sonnei*, *Shigella boydii*, *Enterobacter asburiae*), environmental and aquatic taxa (e.g., *Flavobacterium psychrophilum*, *Flavobacterium columnare*, *Bdellovibrio bacteriovorus*), and opportunistic or healthcare-associated organisms (e.g., *Acinetobacter nosocomialis*, *Acinetobacter junii*, *Chryseobacterium anthropi*). Together, these findings highlight pronounced site-level heterogeneity in microbial composition across the sampled environments (Table 1).

**Fig 1:**
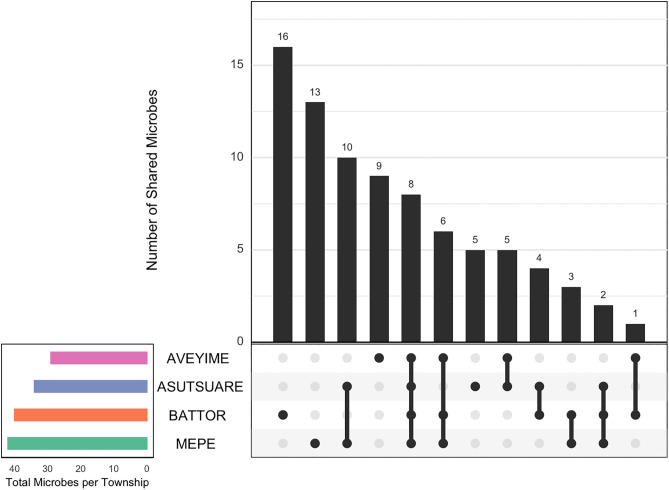
UpSet plot showing the distribution and overlap of microbial taxa across townships. The left horizontal bar chart **(A)** represents the total number of unique microbial taxa identified within each individual township. The main vertical bar chart **(B)**, together with the intersection matrix **(C)**, shows the number of microbial taxa present exclusively in specific township combinations, as indicated by dark dots, and absent from the remaining townships.

Across all the towns, bank and mid-river samples generally exhibited higher microbial richness compared with puddle and canal drain samples. Unique taxa were detected at multiple sites, indicating localized microbial signatures within and between communities. There were nine unique microbes identified in the Aveyime Township, none of which were detected in the other townships. Similarly, 5, 16, and 13 unique microbes were identified in Asutsuare, Battor and Mepe respectively. The UpSet plot (Fig 1) further illustrates the distribution and overlap of microbial taxa across the four townships, highlighting both shared microbial communities and township-specific taxa.

### Microbial community structure and beta diversity

**Fig 2:**
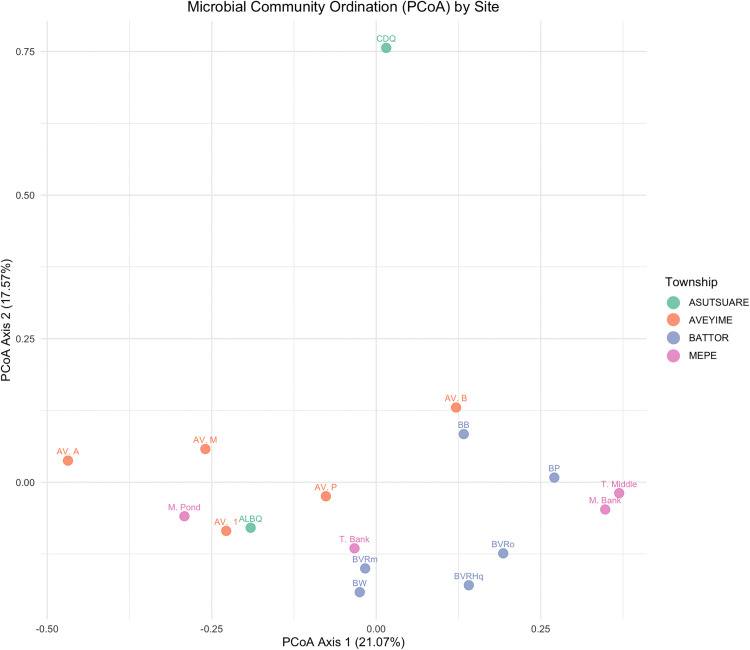
Principal Coordinates Analysis (PCoA) plot representing spatial variations in microbial community compositions across sampling sites and towns. Each point represents a unique sampling site with points coloured by townships (Mepe, Battor, Aveyime, Asutsuare). The distance between points reflects the degree of dissimilarity in the microbial composition. The plot uses two main axes, PCoA Axis 1 (explaining 21.57% of the variation) and PCoA Axis 2 (explaining 17.57% of the variation), to capture the largest dimensions of microbial community differences.

Principal Coordinates Analysis (PCoA) was used to assess beta diversity and examine differences in microbial community composition across sampling sites and townships (Fig 2). The first two principal coordinate axes explained 21.57% (PCoA Axis 1) and 17.57% (PCoA Axis 2) of the total variation, capturing the major gradients in community dissimilarity among samples. Distinct clustering and dispersion patterns were observed across towns with samples from Battor clustering within the lower right quadrant of the ordination space, with relatively uniform spacing, indicating a moderately consistent microbial community structure across sites. In contrast, Aveyime samples exhibited greater dispersion, reflecting higher within-site variability in community composition. Mepe samples were heterogeneously distributed, with bank and mid-river samples clustering together yet clearly separated from other sites within the town, suggesting spatial heterogeneity in microbial communities within the township. Samples from Asutsuare displayed the greatest degree of dispersion in ordination space, indicating pronounced variation in microbial community composition among sites. Notably, the Asutsuare bank sample (ALBQ) clustered closely with samples AV.1 (Aveyime) and M. Pond (Mepe), suggesting partial overlap in microbial community structure across townships. This overlap may reflect shared environmental characteristics or hydrological connectivity influencing microbial assemblages at these sites.

### Risk-based stratification of microorganisms with ecological subclassification

The identified microorganisms were classified both by risk levels and ecological origin. Microorganisms were stratified broadly by risk levels into high, medium, and low categories. Within each risk level, organisms were further categorized according to their ecological classification, such that healthcare-associated pathogens, environmental/aquatic organisms, enteric pathogens, and opportunistic organisms were represented as subsets of the respective risk categories (Table 2).

**Table 2:** Distribution of microbial taxa across risk levels and ecological categories.

Town	Risk Level	Ecological Category	Frequency	Specific Microbes	Associated Diseases Summary
ASUTSUARE	High	Healthcare-associated	6	*Acinetobacter baumannii; Klebsiella oxytoca; Klebsiella pneumoniae; Pseudomonas aeruginosa; Stenotrophomonas maltophilia; Serratia marcescens*	Major nosocomial infections including pneumonia, bloodstream infections, surgical-site infections, Urinary Tract Infections; Multi-Drug Resistance concerns.
		Environmental/Aquatic	1	*Legionella* sp.	Legionnaires’ disease, Pontiac fever.
		Enteric	5	*Escherichia coli; Salmonella enterica; Shigella flexneri; Shigella sonnei; Cronobacter sakazakii*	Diarrhea, gastroenteritis, neonatal infection risk (*C. sakazakii*).
		Opportunistic	0		
	Medium	Healthcare-associated	6	*Enterobacter aerogenes; Enterobacter cloacae; Enterobacter hormaechei; Enterobacter ludwigii; Enterobacter* sp*.; Citrobacter freundii*	Healthcare-associated infections including bacteremia, Urinary Tract Infections, wound infections.
		Environmental/Aquatic	0		
		Enteric	3	*Aeromonas hydrophila; Bacillus cereus; Aeromonas* sp.	Gastroenteritis, wound infections (*Aeromonas*); food poisoning (*B. cereus*).
		Opportunistic	7	*Pseudomonas putida; Pantoea agglomerans; Burkholderia* sp*.; Bacteroides* sp*.; Comamonas* sp*.; Comamonas testosteroni Sphingomonas* sp.	Opportunistic infections (wound, device-related, respiratory).
	Low	Healthcare-associated	1	*Propionibacterium acnes*	Low-virulence skin commensal; device-related infections.
		Environmental/Aquatic	4	*Flavobacterium* sp*.; Iodobacter* sp*.; Acidovorax* sp*. Desulfovibrio* sp.	Low direct human risk; fish/plant-associated bacteria.
		Enteric	0		
		Opportunistic	1	*Rickettsia endosymbiont*	Endosymbiont; very low pathogenicity.
AVEYIME	High	Healthcare-associated	4	*Acinetobacter baumannii; Pseudomonas aeruginosa; Stenotrophomonas maltophilia; Burkholderia cepacia*	Nosocomial infections with Multi-Drug Resistance trends; pneumonia, bloodstream infections.
		Environmental/Aquatic	0		
		Enteric	1	*Escherichia coli*	Diarrheal disease, Urinary Tract Infections.
		Opportunistic	1	*Stenotrophomonas maltophilia*	Opportunistic Multi-Drug Resistance pathogen.
	Medium	Healthcare-associated	3	*Acinetobacter* sp*.; Alcaligenes* sp*.; Stenotrophomonas* sp.	Device-associated, wound, respiratory opportunistic infections.
		Environmental/Aquatic	3	*Flavobacterium* sp*.; Flavobacterium columnare; Flavobacterium psychrophilum*	Environmental/fish pathogens; occasional rare human infections.
		Enteric	4	*Aeromonas hydrophila; Aeromonas veronii; Chitinimonas taiwanensis; Bacillus* sp.	Gastroenteritis, wound infections.
		Opportunistic	9	*Comamonas* sp*.; Comamonas aquatica; Comamonas testosteroni; Pseudomonas putida; Pseudomonas* sp*.; Chryseobacterium* sp*.; Sphingomonas* sp*.; Bacillus* sp*.; Alcaligenes* sp.	Opportunistic infections‚ Respiratory, wound, bloodstream.
	Low	Healthcare-associated	1	*Brevundimonas* sp.	Rare opportunistic infections; generally low risk.
		Environmental/Aquatic	2	*Acidovorax* sp*.; Enhydrobacter aerosaccus*	Environmental bacteria; low human disease risk.
		Enteric	0		
		Opportunistic	1	*Rickettsia endosymbiont*	Very low pathogenicity.
BATTOR	High	Healthcare-associated	7	*Acinetobacter baumannii; Klebsiella pneumoniae; Pseudomonas aeruginosa; Stenotrophomonas maltophilia; Klebsiella* sp.; *Serratia* sp.; *Mycobacterium* sp.;	Major nosocomial pathogens; Multi-Drug Resistance; pneumonia, Urinary Tract Infections, bloodstream infections.
		Environmental/Aquatic	1	*Legionella* sp.	Legionnaires’ disease.
		Enteric	3	*Escherichia coli; Salmonella enterica; Shigella* sp.	Enteric infections; diarrhea; neonatal infection risk.
		Opportunistic	2	*Burkholderia cepacia; Stenotrophomonas* sp.	Multi-Drug Resistance respiratory opportunistic pathogens.
	Medium	Healthcare-associated	8	*Acinetobacter calcoaceticus; Acinetobacter johnsonii; Acinetobacter junii; Acinetobacter* sp*.; Citrobacter freundii; Propionibacterium acnes; Staphylococcus* sp*.; Streptococcus* sp.	Opportunistic Healthcare-associated Infections: wound, device-related, skin/soft tissue infections.
		Environmental/Aquatic	6	*Pseudomonas* sp*.; Rhodococcus* sp*.; Flavobacterium* sp*.; Thermomonas haemolytica; Porphyromonas* sp*.; Chryseobacterium* sp.	Opportunistic and environmental microbes; occasional infections.
		Enteric	4	*Aeromonas hydrophila; Aeromonas veronii; Aeromonas* sp*.; Bacillus cereus*	Gastroenteritis, wound infections.
		Opportunistic	12	*Burkholderia* sp*.; Chryseobacterium anthropi; Chryseobacterium hominis; Pseudomonas putida; Pseudomonas alcaligenes; Pseudomonas stutzeri; Bacillus* sp*.; Alcaligenes faecalis; Mycobacterium* sp*.; Bacteroides* sp*.; Propionibacterium* sp*.; Porphyromonas* sp.	Opportunistic infections including respiratory, wound, bloodstream.
	Low	Healthcare-associated	3	*Acinetobacter beijerinckii; Acinetobacter lwoffii; Acinetobacter radioresistens*	Low-virulence opportunists.
		Environmental/Aquatic	4	*Flavobacterium* sp*.; Flavobacterium chungnamense; Flavobacterium macrobrachii; Bdellovibrio bacteriovorus*	Environmental species; low human disease risk.
		Enteric	0		
		Opportunistic	1	*Sphingobacterium* sp.	Rare opportunistic infections.
MEPE	High	Healthcare-associated	6	*Acinetobacter baumannii; Pseudomonas aeruginosa; Burkholderia cepacia; Klebsiella oxytoca; Serratia marcescens; Pseudomonas sp.*	Nosocomial high-risk pathogens; Multi-Drug Resistance; pneumonia, sepsis, Urinary Tract Infections.
		Environmental/Aquatic	1	*Legionella* sp.	Legionnaires’ disease.
		Enteric	5	*Escherichia coli; Salmonella enterica; Shigella boydii; Shigella flexneri; Cronobacter sakazakii*	Severe gastroenteritis; neonatal *Cronobacter* infections.
		Opportunistic	2	*Stenotrophomonas maltophilia; Burkholderia cepacia*	Multi-Drug Resistance respiratory opportunists.
	Medium	Healthcare-associated	10	*Enterobacter aerogenes; Enterobacter cloacae; Klebsiella* sp*.; Acinetobacter johnsonii; Acinetobacter calcoaceticus; Acinetobacter beijerinckii; Acinetobacter lwoffii; Acinetobacter radioresistens; Acinetobacter tjernbergiae; Acinetobacter* sp.	Healthcare-associated opportunists.
		Environmental/Aquatic	6	*Flavobacterium* sp*.; Rhodococcus* sp*.; Iodobacter* sp*.; Flavisolibacter* sp*.; Flavobacterium chungnamense; Flavobacterium macrobrachii*	Environmental/fish/soil organisms.
		Enteric	2	*Aeromonas hydrophila; Aeromonas veronii*	Gastroenteritis, wound infections.
		Opportunistic	13	*Pseudomonas putida; Pseudomonas* sp*.; Rhodococcus erythropolis; Rhodococcus* sp*.; Chryseobacterium* sp*.; Serratia* sp*.; Enterobacter asburiae; Enterobacter ludwigii; Pantoea agglomerans; Bacillus* sp*.; Acinetobacter* sp*.; Xanthomonas* sp*.; Sphingobacterium* sp.	Opportunistic infections; moderate risk.
	Low	Healthcare-associated	4	*Acinetobacter* sp*.; Pseudomonas putida; Propionibacterium acnes; Mycobacterium* sp.	Low-virulence Healthcare-associated Infections or environmental species.
		Environmental/Aquatic	3	*Pectobacterium carotovorum; Bdellovibrio bacteriovorus; Thermomonas haemolytica*	Plant/soil-associated species; low human risk.
		Enteric	0		
		Opportunistic	0		

**Fig 3:**
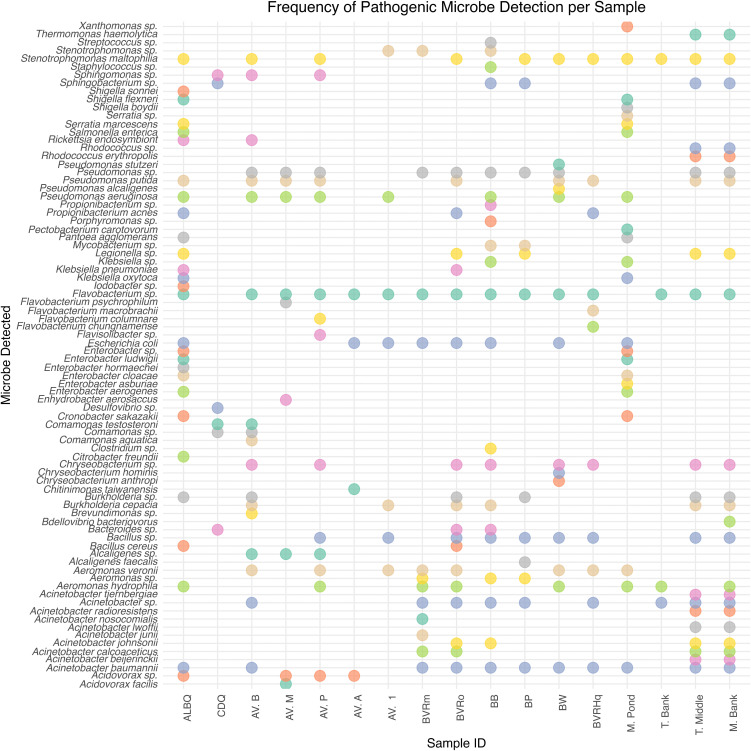
Bubble plot providing an overview of the different pathogenic microbes found at specific sampling sites, as well as the overall microbial prevalence. The figure illustrates the detection patterns of clinically relevant microorganisms classified as high- or medium-risk across sampling sites. Each point represents the presence of a pathogenic or potentially pathogenic taxon at a given site. The distribution highlights site-specific and repeated detection patterns of organisms of public health importance across towns and hydrological settings.

The spatial frequency and site-level distribution of pathogenic and potentially pathogenic taxa are illustrated in Fig 3 providing spatial context to the observed risk profiles. The figure demonstrates that clinically relevant microorganisms were not uniformly distributed across sites but instead showed clear site-specific and hydrology-linked patterns. Bank, mid-river, and pond environments exhibited higher frequencies of detection of pathogenic species compared with canal drain and puddle sites, Multiple pathogenic taxa were detected at more than one site within the same town, while other taxa were restricted to single sampling locations.

Variation in detection frequency was observed across towns, with differences in the number of sites at which pathogenic taxa were detected. Sites exhibiting higher clustering of pathogenic taxa corresponded to towns with greater representation of high- and medium-risk organisms in the risk-based classification (Table 2).

### Town-level distribution of risk-classified microorganisms

#### Asutsuare.

Microbes were found across all risk levels and spanned most of the ecological categories. High-risk microbes found included healthcare-associated pathogens such as *Acinetobacter baumanii*, *Klebsiella oxytoca*, *Klebsiella pneumoniae*, and *Serratia marcescens*, enteric pathogens such as *Escherichia coli*, *Salmonella enterica*, *Shigella flexneri*, and *Cronobacter sakazakii*; and *Legionella* sp*.* as the only environmental/aquatic organism. No high-risk opportunistic microbes were detected. Medium-risk organisms included healthcare-associated pathogens such as *Citrobacter freundii*, and multiple *Enterobacter* species; and enteric pathogens including *Aeromonas hydrophila*, and *Bacillus cereus*. Opportunistic pathogens included *Pseudomonas putida*, *Pantoea agglomerans*, *Burkholderia* sp*.*, *Shingomonas* sp*.* and *Comamonas testosteroni and* no medium-risk environmental/aquatic microbes were found. Low-risk organisms included *Propionibacterium acnes* as the single healthcare-associated pathogen; *Flavobacterium* sp*.*, *Iodobacter* sp*.*, *Acidovorax* sp*.*, and *Desulfovibrio* sp*.* as environmental/aquatic microbes; and *Rickettsia endosymbiont* as the single opportunistic pathogen. No low-risk enteric microbes were detected.

#### Aveyime.

Samples from the town harboured a high burden of medium-risk opportunistic taxa. The high-risk microbes detected included healthcare-associated pathogens: *Acinetobacter baumannii*, *Pseudomonas aeruginosa*, *Stenotrophomonas maltophilia*, and *Burkholderia cepacia*. *Escherichia coli* and *S. maltophilia* were the sole enteric and opportunistic pathogens identified respectively. No high-risk environmental/aquatic microbes were detected. Medium-risk organisms detected include the healthcare-associated pathogens: *Acinetobacter* sp*.*, *Alcaligenes* sp*.*, and *Stenotrophomonas* sp*.* while multiple *Flavobacterium* species were identified as environmental/aquatic microbes. The Enteric pathogens detected were *Aeromonas hydrophila*, *Aeromonas veronii*, *Bacillus* sp*.*, and *Chitinimonas taiwanensis* and opportunistic pathogens included *Alcaligenes* sp*.,* *Bacillus* sp*.*, and multiple *Comamonas* *and* *Pseudomonas* species. In the low-risk category, *Brevundimonas* sp*.* and *Rickettsia endosymbiont* were the sole low-risk healthcare-associated and opportunistic pathogens identified respectively. *Acidovorax* sp*.*, and *Enhydrobacter aerosaccus* were detected as low-risk environmental/aquatic microbes and no low-risk enteric pathogens were identified.

#### Battor.

Battor showed the widest distribution of microorganisms across all risk levels and ecological categories. High-risk healthcare-associated pathogens such as *Acinetobacter baumanii*, *Burkholderia cepacia*, *Klebsiella oxytoca*, and *Serratia marcescens* were identified whiles *Legionella* sp. was the only high-risk environmental/aquatic organism detected. The Enteric pathogens detected included *Escherichia coli*, *Shigella* sp. and *Salmonella enterica,* while opportunistic pathogens identified were *Burkholderia cepacia* and *Stenotrophomonas* sp. Medium-risk organisms included healthcare-associated pathogens, such as multiple *Acinetobacter* species, *Citrobacter freundii*, and *Propionibacterium acnes*, as well as environmental/aquatic taxa including *Pseudomonas* sp., *Rhodococcus* sp., and *Thermomonas haemolytica*. This risk category also comprised potentially pathogenic enteric organisms, such as multiple *Aeromonas* species and *Bacillus cereus*, together with opportunistic pathogens including *Burkholderia* sp., *Chryseobacterium anthropi*, *Chryseobacterium hominis*, *Alcaligenes faecalis*, and multiple *Pseudomonas* species. Low-risk healthcare-associated pathogens detected included *Acinetobacter beijerinckii*, *Acinetobacter lwoffii*, and *Acinetobacter radioresistens*. Low-risk environmental/aquatic organisms comprised multiple *Flavobacterium* species and *Bdellovibrio bacteriovorus*, while *Sphingobacterium* sp. was the only low-risk opportunistic pathogen identified. No enteric pathogens were detected within this risk category.

#### Mepe.

Microorganisms detected in Mepe spanned all three risk levels and several ecological categories. High-risk organisms included healthcare-associated pathogens such as *Acinetobacter baumannii*, *Pseudomonas aeruginosa*, and *Burkholderia cepacia*. *Legionella* sp. was detected as the sole high-risk environmental/aquatic organism. High-risk enteric pathogens comprised *Escherichia coli*, *Salmonella enterica*, and multiple *Shigella* species, while *Stenotrophomonas maltophilia* and *Burkholderia cepacia* were the only high-risk opportunistic pathogens identified. Medium-risk organisms included healthcare-associated pathogens such as multiple *Acinetobacter* and *Enterobacter* species, as well as *Klebsiella* sp. Environmental/aquatic organisms detected at this risk level included *Flavisolibacter* sp. and multiple *Flavobacterium* species. The enteric pathogens identified were *Aeromonas hydrophila* and *Aeromonas veronii*, while opportunistic pathogens comprised multiple *Pseudomonas*, *Rhodococcus*, and *Enterobacter* species. Low-risk organisms included healthcare-associated taxa such as *Acinetobacter* sp. and *Pseudomonas putida*. Environmental/aquatic organisms detected at this risk level included *Pectobacterium carotovorum*, *Bdellovibrio bacteriovorus*, and *Thermomonas haemolytica*. No low-risk enteric or opportunistic pathogens were detected.

## Discussion

This study provides molecular evidence of microbial contamination and potential public health risk in downstream communities along the Volta River following the 2023 controlled spillage of the Akosombo and Kpong dams. Using 16S rRNA gene sequencing, we identified diverse microbial communities across multiple water sources in Asutsuare, Aveyime, Battor, and Mepe, including enteric pathogens, opportunistic organisms, and healthcare-associated bacteria of established public health relevance. Recent evidence from a longitudinal study has shown that flood-impacted aquatic environments can remain microbiologically compromised for extended periods after floodwaters recede, particularly in low and middle-income settings where sanitation and water infrastructure are disrupted [4]. The findings from this study are consistent with these observations and highlight the persistence of microbial hazards in downstream environments months after flooding events.

Across all the towns, microbial communities were dominated by Proteobacteria, with frequent co-occurrence of Bacteroidetes and Firmicutes. Similar taxonomic patterns have been reported in flood-affected rivers and disturbed freshwater systems, where increased nutrient loading and hydrological disturbance favour Proteobacteria-rich assemblages [32,33]. In this study, bank and mid-river samples generally exhibited higher microbial richness than puddle and canal drain samples. Comparable richness patterns have been documented in recent flood and riverine microbiome studies, where hydrologically connected environments support more diverse microbial communities than isolated or stagnant waters [34,35]. For downstream communities that rely on riverbanks and mid-channel waters for domestic activities, this increased diversity includes a broader spectrum of organisms with potential public health implications [36].

Beta diversity analysis further demonstrated pronounced spatial heterogeneity in microbial community composition across sampling sites and towns. Partial clustering by town was observed alongside substantial within-town dispersion, particularly in Asutsuare and Mepe. Similar patterns of spatial heterogeneity have been described in aquatic microbial ecology studies, where local environmental conditions, water source type, and contamination inputs shape microbial communities over short spatial scales [37]. The overlap observed between selected sites across different towns likely reflects shared hydrological connectivity within the Volta River system, a feature that has been noted in large river basins affected by dam operations and controlled spillages [38].

A key public health signal in this study is the repeated detection of enteric-associated organisms, including *Escherichia coli*, *Salmonella enterica*, *Shigella* sp., and *Cronobacter sakazakii*, across multiple towns and water source types. Floodwaters are known to mobilize faecal contaminants from sanitation facilities, agricultural areas, and animal waste into surface and groundwater sources, thereby increasing the risk of waterborne disease transmission [4,39]. Recent studies in flood-affected settings have consistently linked elevated levels of enteric bacteria with increased incidence of diarrhoeal and gastrointestinal diseases, particularly among children and other vulnerable populations [40]. The strong enteric signal observed in Mepe’s pond sample is therefore of particular concern given the documented severity of flooding and displacement in this community.

In addition to enteric pathogens, this study identified multiple opportunistic and healthcare-associated organisms of clinical relevance, including *Acinetobacter baumannii*, *Klebsiella pneumoniae*, *Pseudomonas aeruginosa*, and *Stenotrophomonas maltophilia*. Recent environmental and public health research increasingly recognizes natural and engineered water systems as reservoirs for such organisms, especially in post-flood contexts where infrastructure damage and altered water flow facilitate environmental dissemination [4,41]. The detection of these taxa in groundwater-associated sources such as wells and boreholes, particularly in Battor, aligns with reports that shallow or poorly protected groundwater sources are vulnerable to contamination following flooding events [42].

The integration of ecological classification with risk-based stratification provides a structured framework for interpreting microbial findings in a public health context. Similar risk-oriented approaches have been recommended in recent environmental health literature which advocates adapting Quantitative Microbial Risk Assessment (QMRA) frameworks for rural and remote community water systems by incorporating source-specific ecological heterogeneity to enable tiered risk prioritization beyond presence-absence reporting [43]. The pathogen-focused spatial analysis in this study demonstrates that pathogenic and potentially pathogenic taxa are not uniformly distributed across sampling sites, with repeated detection at certain locations and restriction to single sites at others. Such spatial heterogeneity has been widely reported in flood-affected water systems and underscores the importance of site-specific surveillance and intervention rather than uniform community-wide risk assumptions [44–47].

Our study also presents with a few limitations. Detection of bacterial DNA using 16S rRNA sequencing does not confirm organism viability or infectivity, and species-level resolution may be limited for some taxa. The cross-sectional design, conducted after floodwaters had receded, does not capture temporal dynamics during the acute flooding phase. In addition, antimicrobial resistance profiles were not assessed, despite the detection of organisms commonly associated with multidrug resistance. Nonetheless, the combined taxonomic, beta diversity, and risk-based analyses provide a comprehensive snapshot of microbial hazards in downstream communities following dam-related flooding. Future studies should prioritize longitudinal sampling across seasons, quantitative microbial assays, culture-based confirmation, and resistance gene profiling, to better characterize ongoing public health risks in downstream flood-affected settings.

Overall, our study highlights that downstream flood-affected communities along the Volta River system are exposed to spatially heterogeneous microbial risks that vary by water source type and location. The persistence of enteric, opportunistic, and healthcare-associated bacteria months after flooding underscores the need for targeted water safety interventions, strengthened environmental surveillance, and context-specific public health responses in communities impacted by dam spillages and extreme hydrological events.

## Supporting information

S1 Fig Microbial diversity, abundance and community composition charts. Graphical summaries of microbial relative abundance and community composition across sampling sites, including Krona plots illustrating the taxonomic distribution of microbial communities detected in each sample.
(DOCX)

S2 Fig Alpha diversity indices and plots. Tabulated alpha diversity indices and associated graphical representations of within-sample microbial diversity across all sampling sites and townships.
(DOCX)

S1 Table GPS coordinates of all 22 sampling sites. Geographic coordinates recorded for each sampling site across the four study townships (Asutsuare, Aveyime, Battor, and Mepe) using a handheld GPS device.
(DOCX)

S2 Table Microbial risk classification data. Tabulated data detailing the township, sampling site, identified microorganism, risk level, ecological category, group classification, and associated disease for all microbial taxa detected across sampling sites.
(XLSX)
