# Supplementary material for: Downstream community risks post dam-spillage flooding along the Volta River in Ghana: potential pathogens and public health implications
Source: PLoS One. 2026 Jul 2;21(7):e0346766. doi: 10.1371/journal.pone.0346766 (PMC13327315; doi:10.1371/journal.pone.0346766)

# Microbial Diversity / Abundance charts

ASUTSUARE

### **ALB Q**: Phyla, Families, Genera


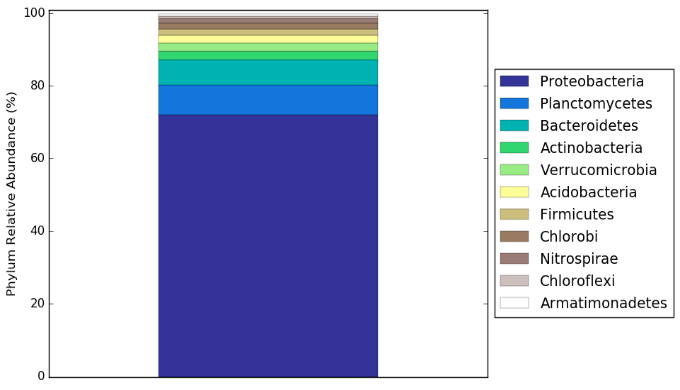


***Phyla***


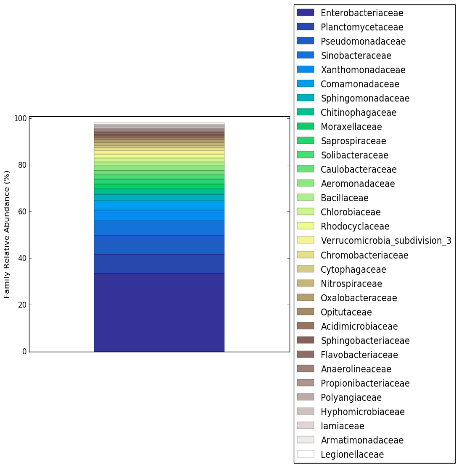

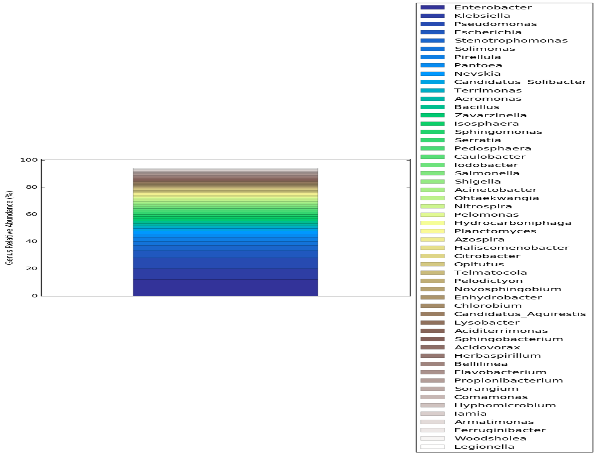


***Families Genera***

CD Q: Phyla, Families, Genera

**
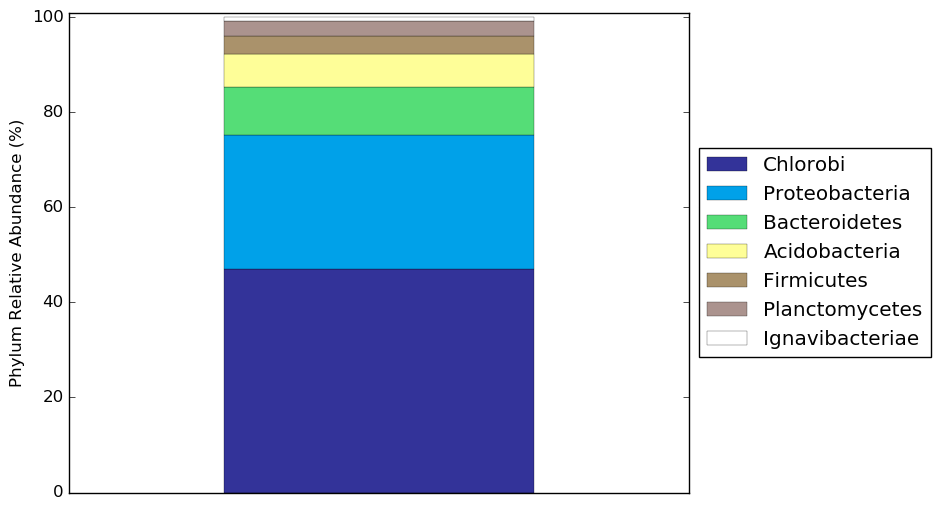
** **
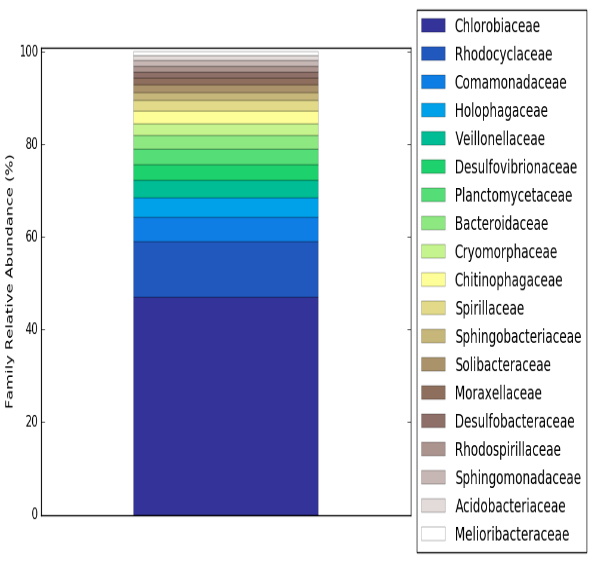
**

***Phyla Families***

**
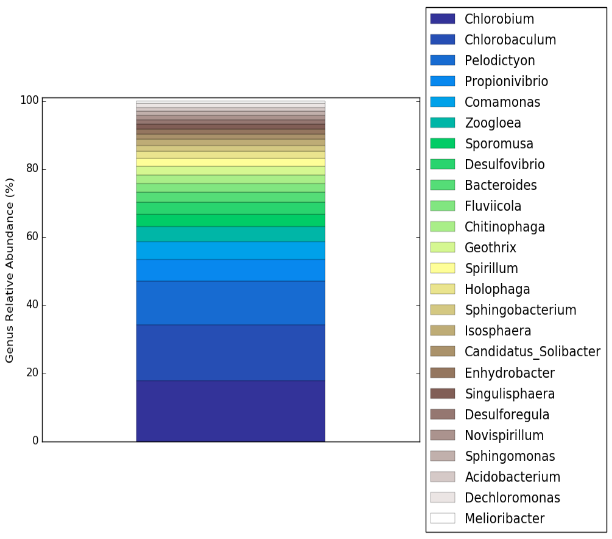
**

***Genera***

## AVEYIME

### **AV. B:** Phyla, Families, Genera


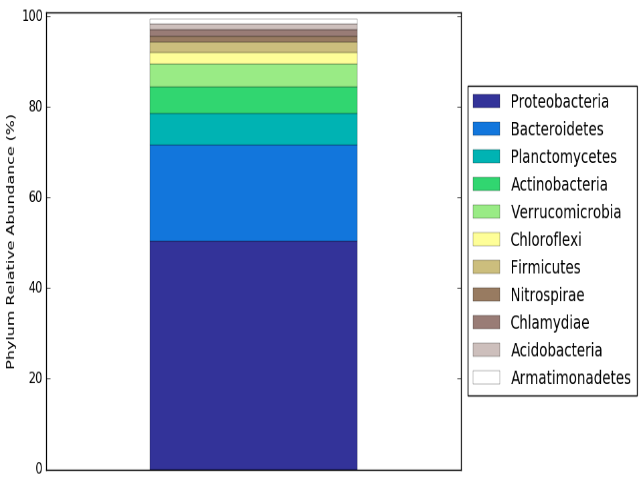

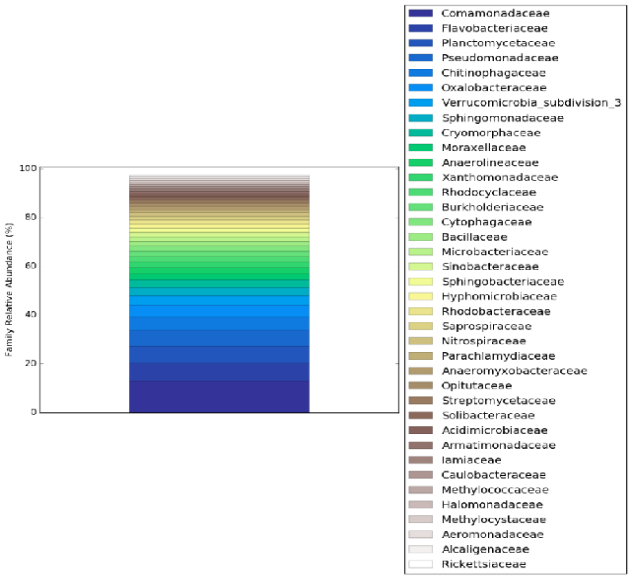


***Phyla Families***


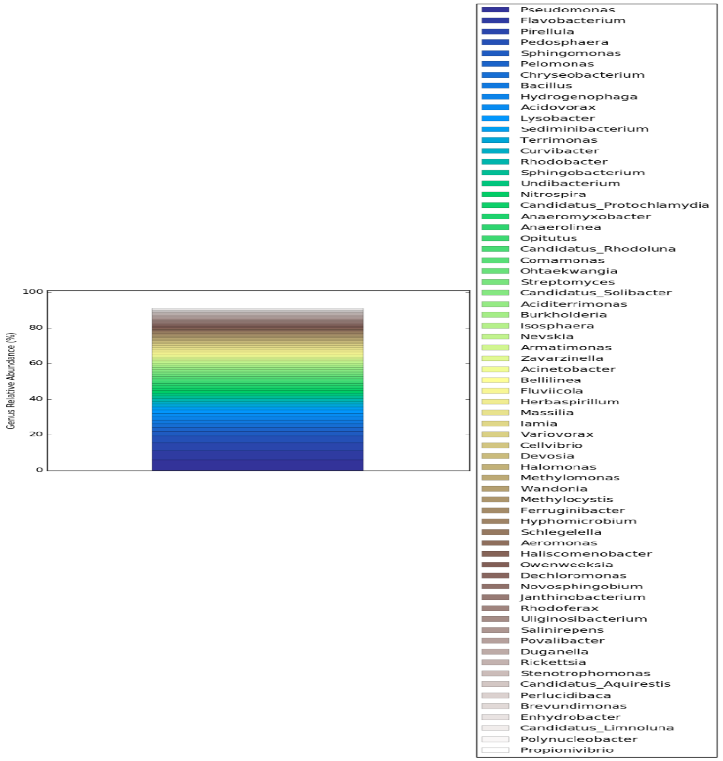


Genera

### **AV. M:** Phyla, Families, Genera


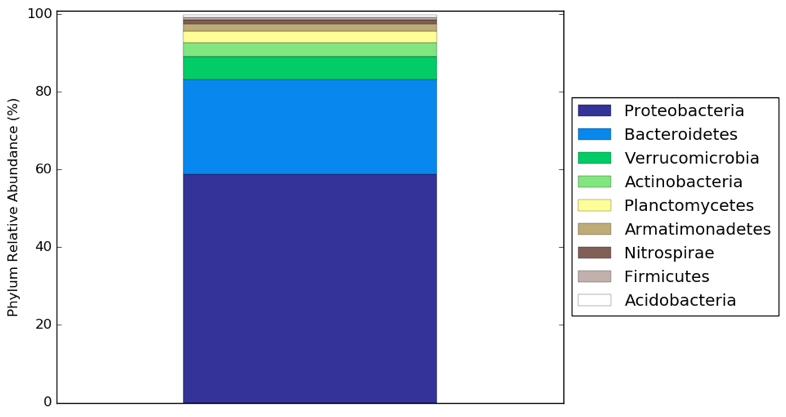

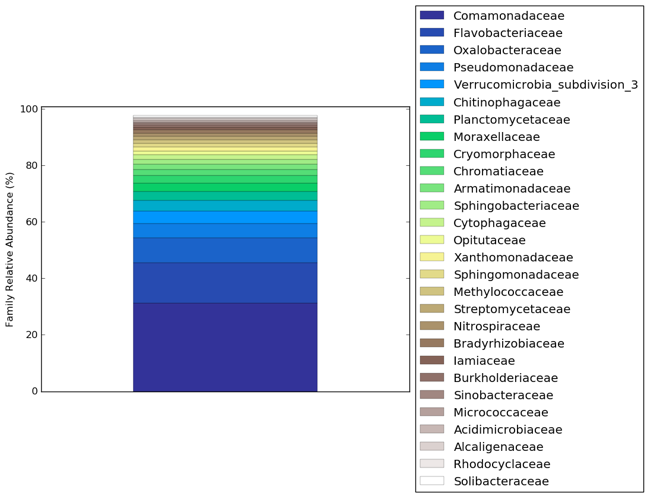


***Phyla Families***


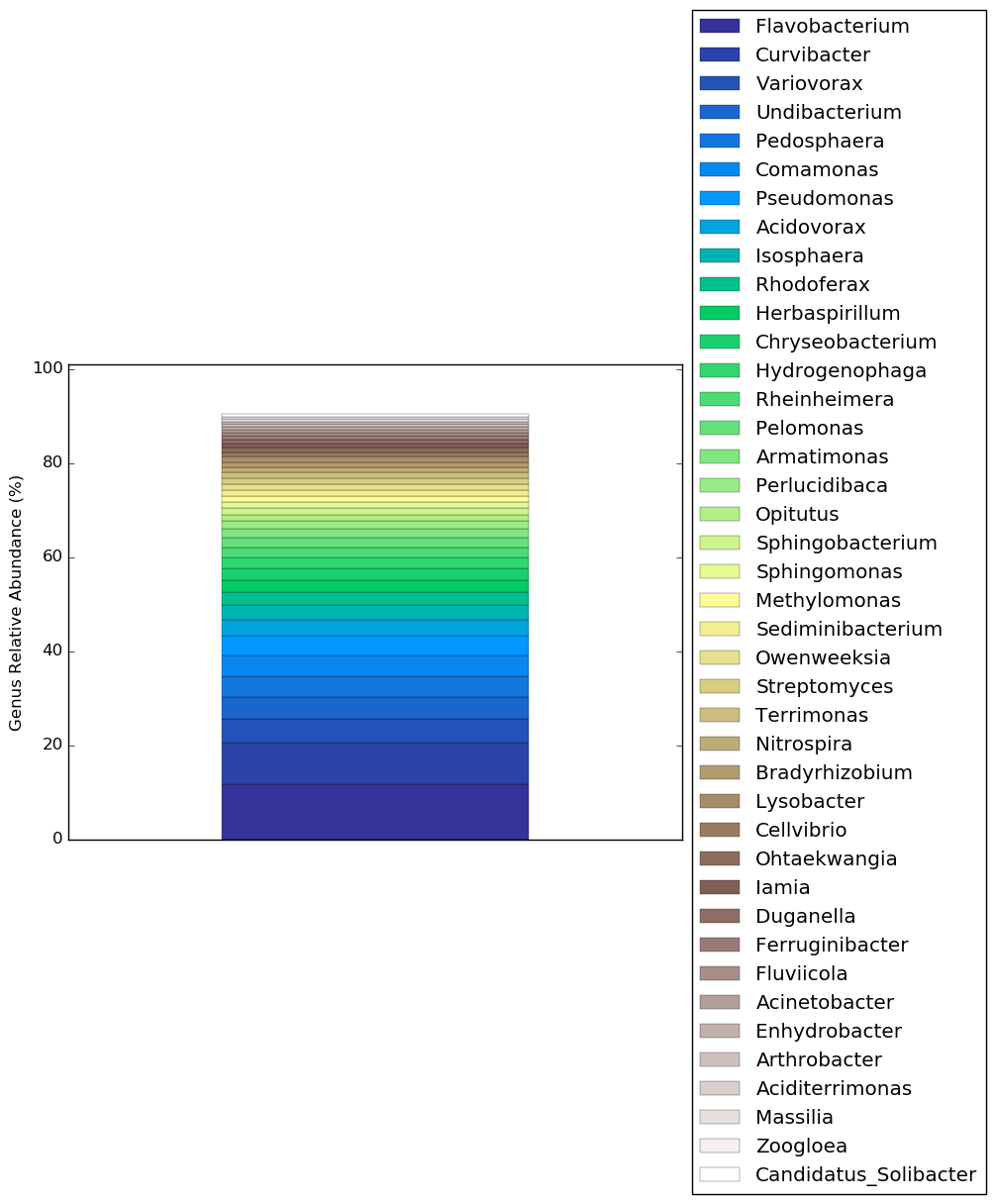


***Genera***

### **AV. P:** Phyla, Families, Genera


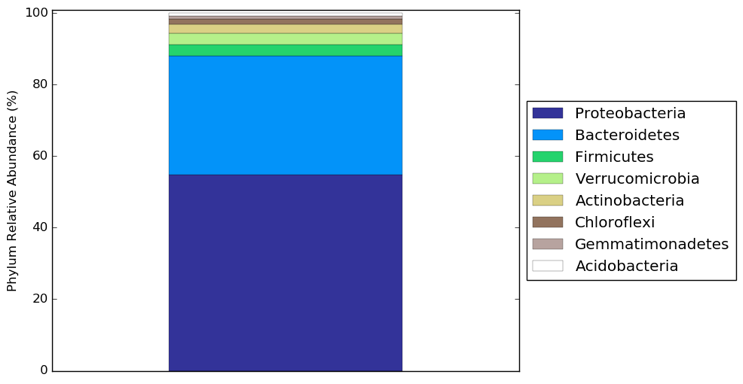

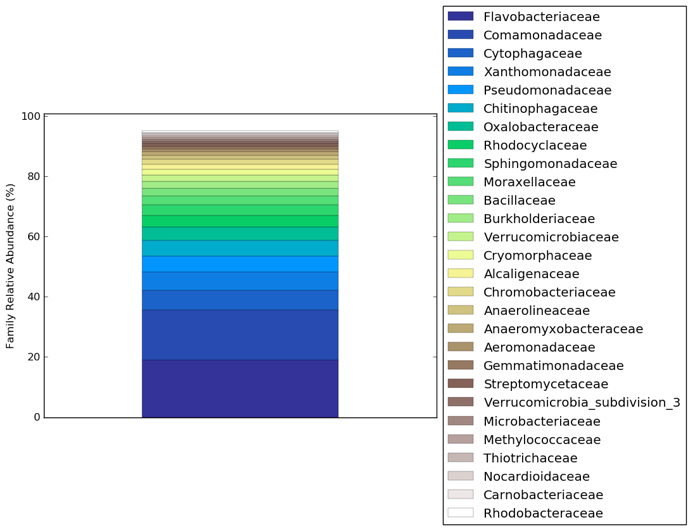


***Phyla Families***


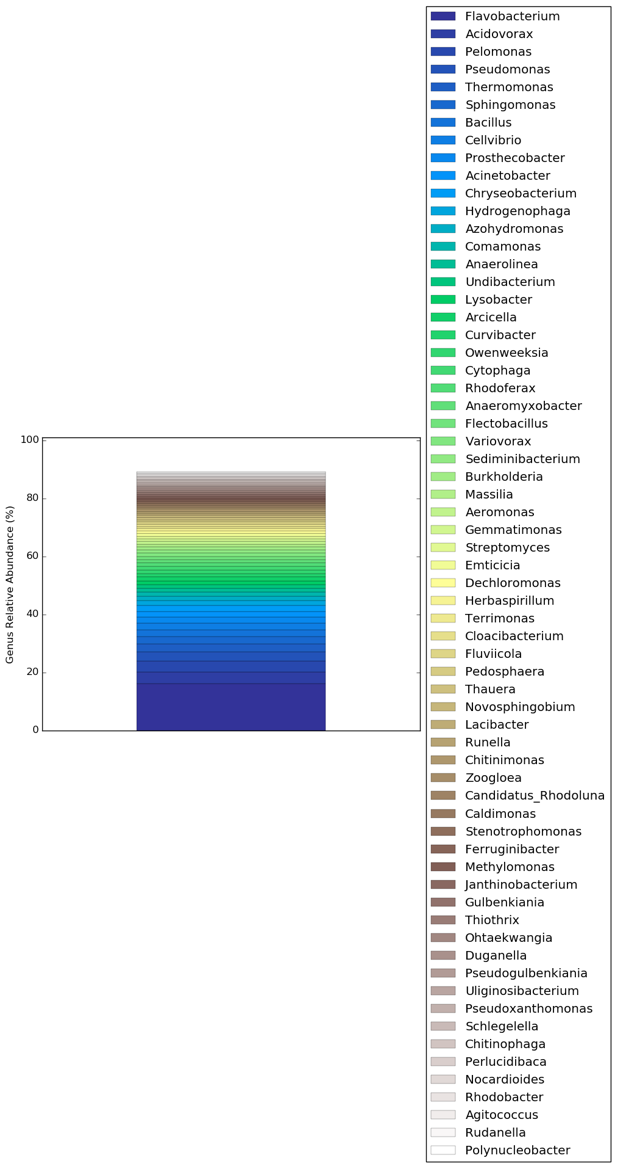


***Genera***

### **AV. A:** Phyla, Families, Genera


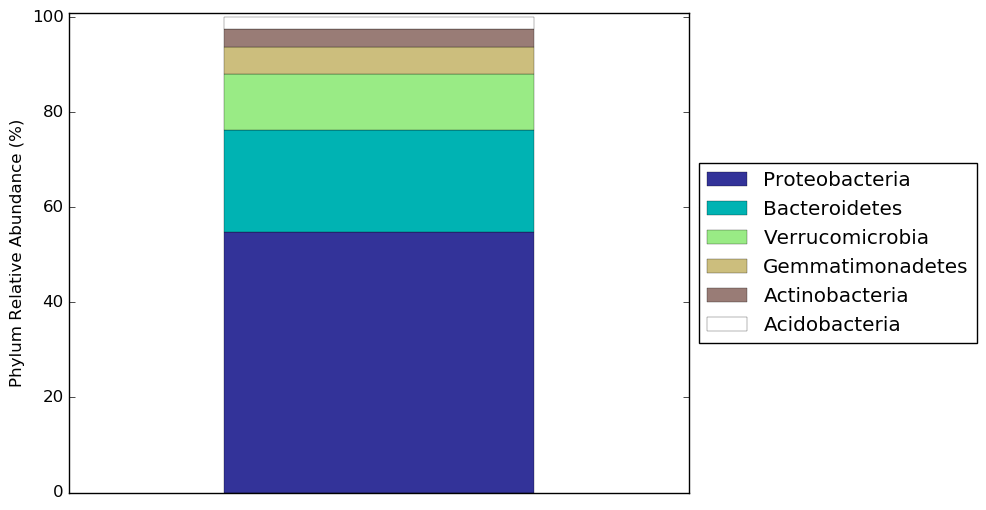


***Phyla***


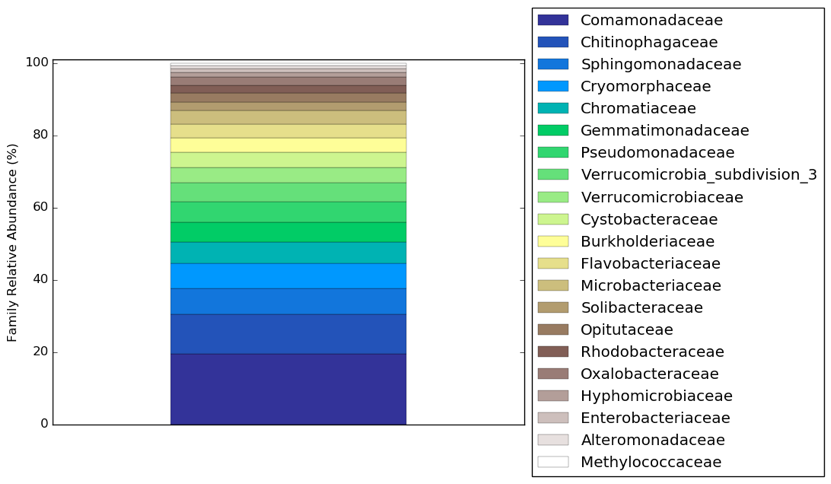


***Families***


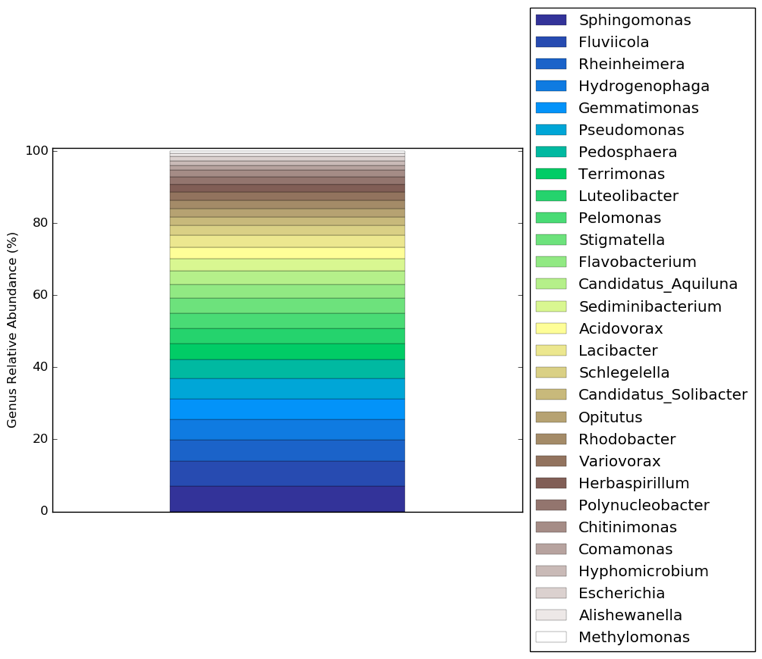


***Genera***

### **AV. 1:** Phyla, Families, Genera


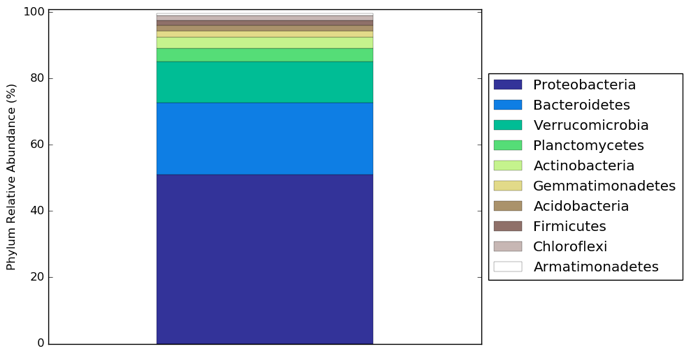

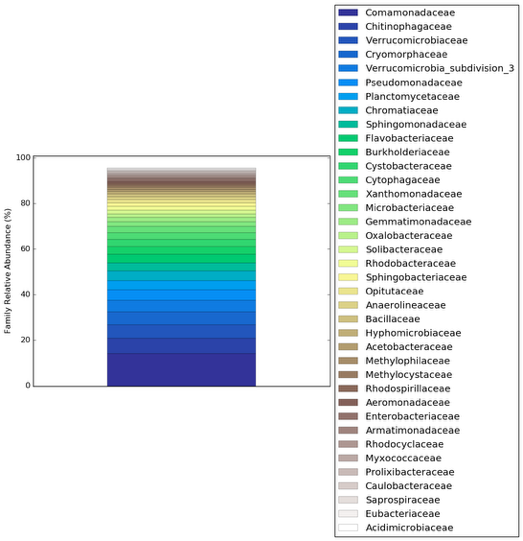


***Phyla Families***


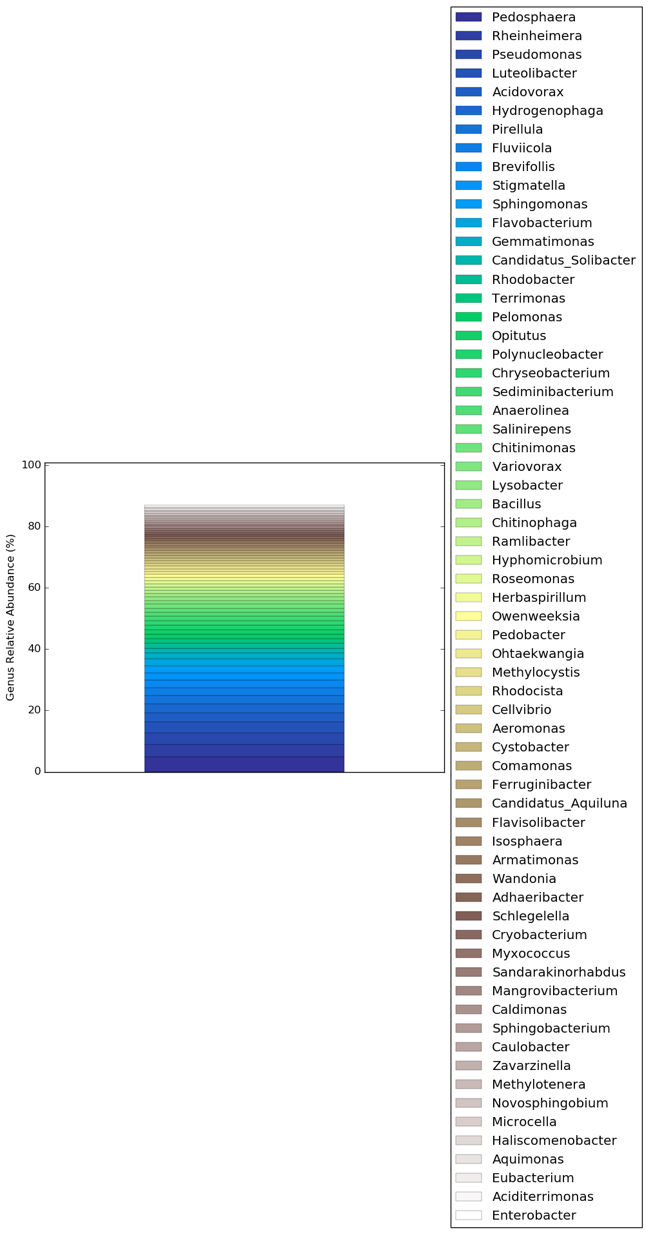


***Genera***

## BATTOR

### **BVRm:** Phyla, Families, Genera


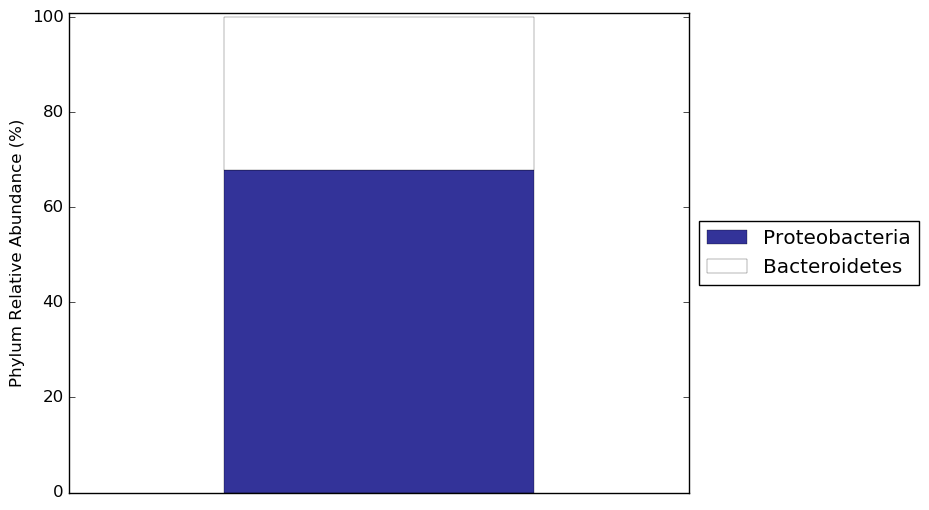


***Phyla***


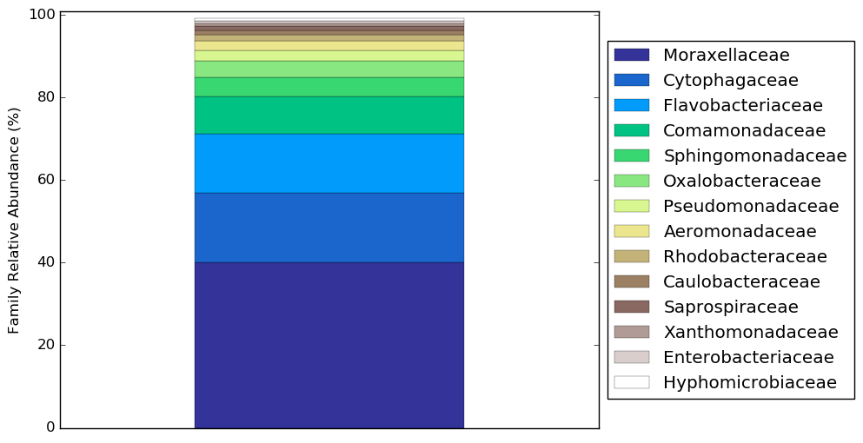


***Families***


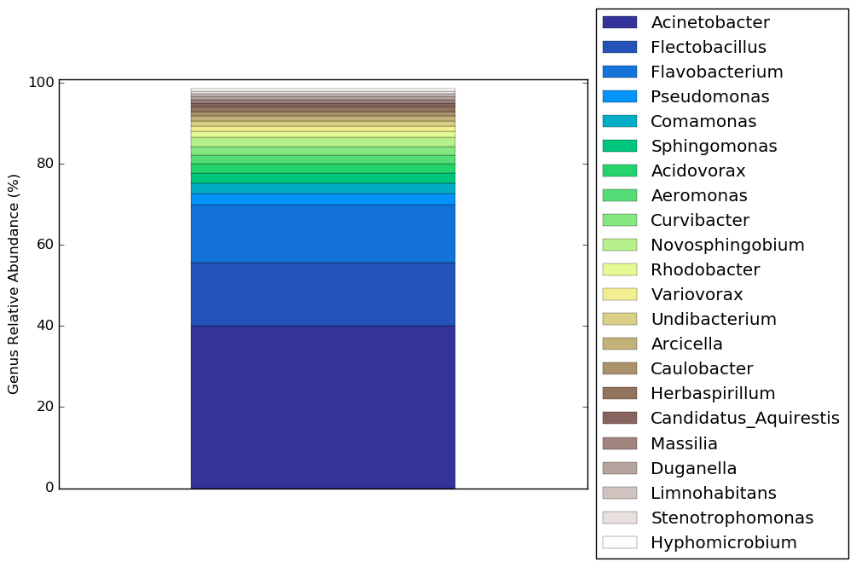


***Genera***

### **BVR_0_** Phyla, Families, Genera


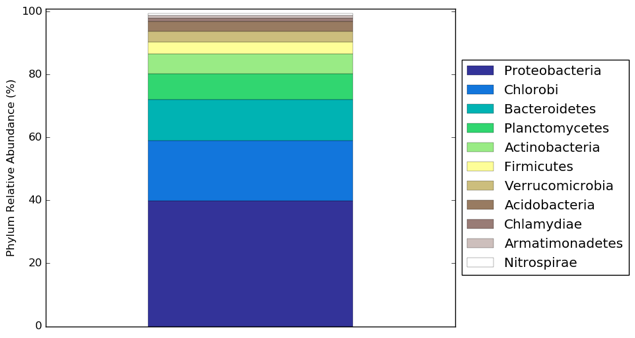


***Phyla***


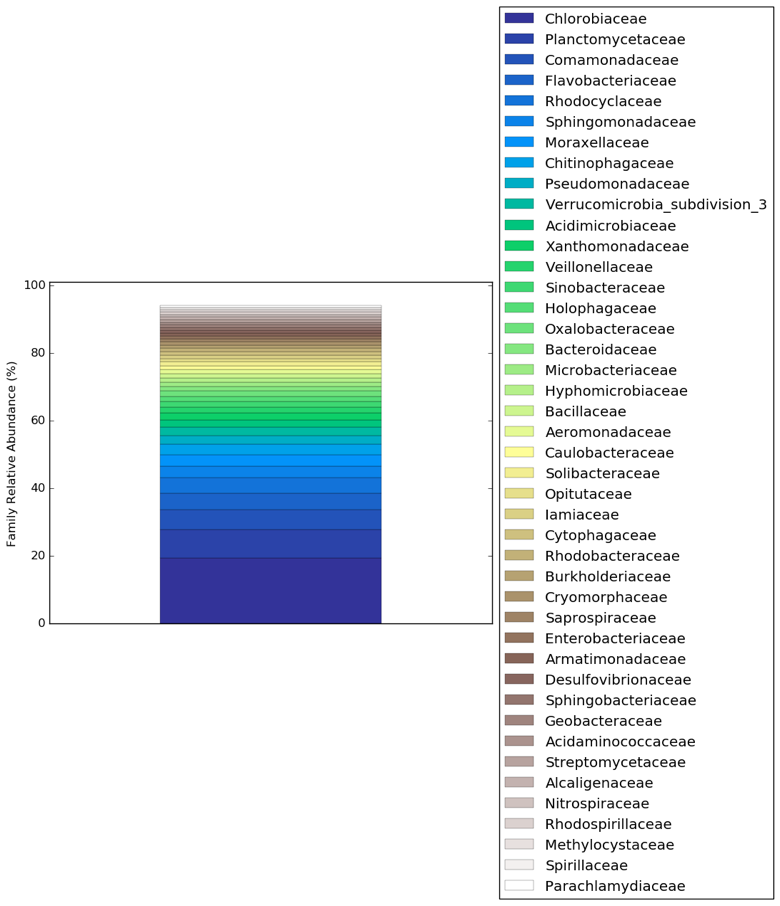

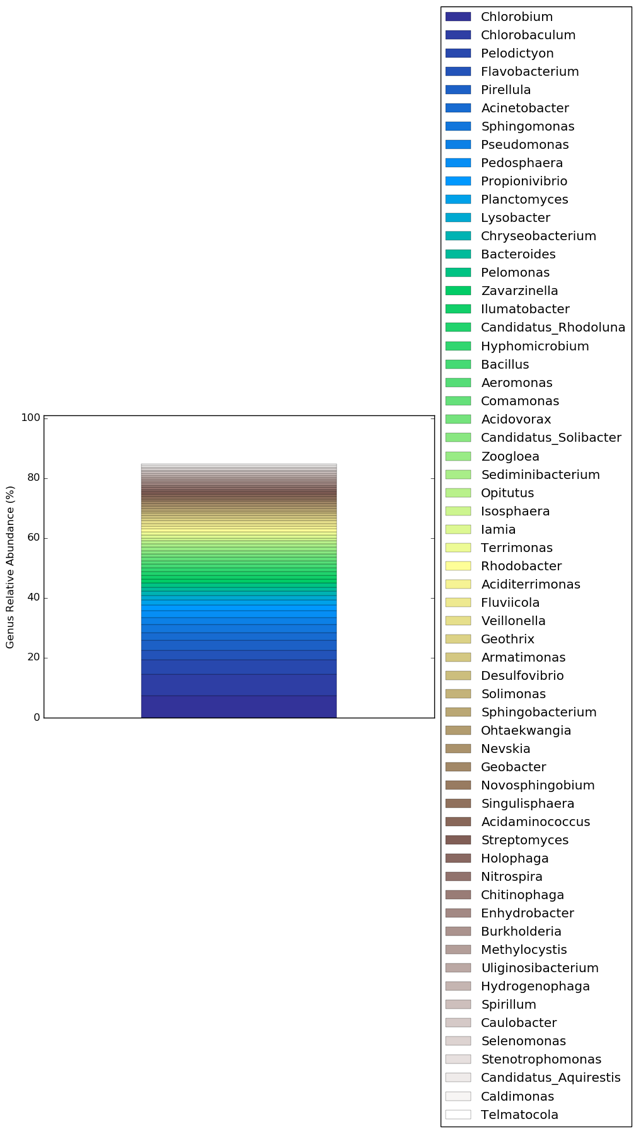


***Families Genera***

### **BB:** Phyla, Families, Genera


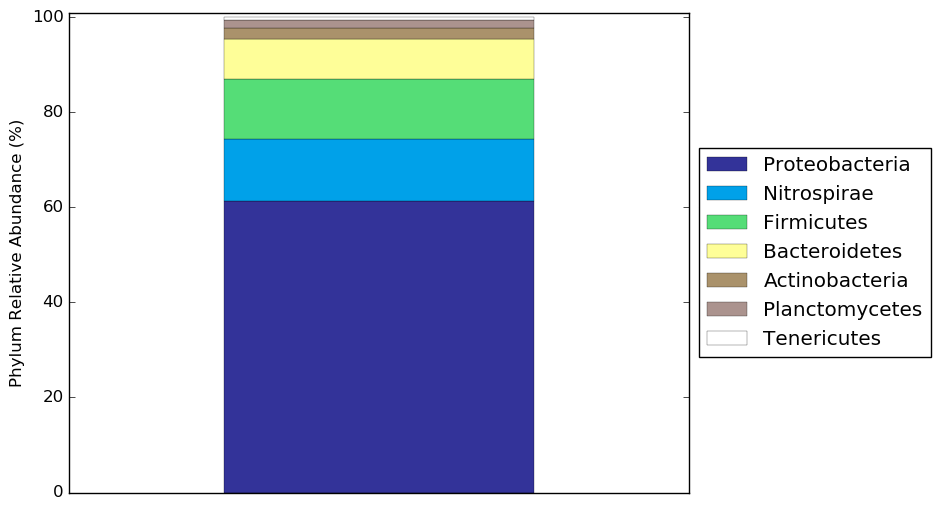


***Phyla***


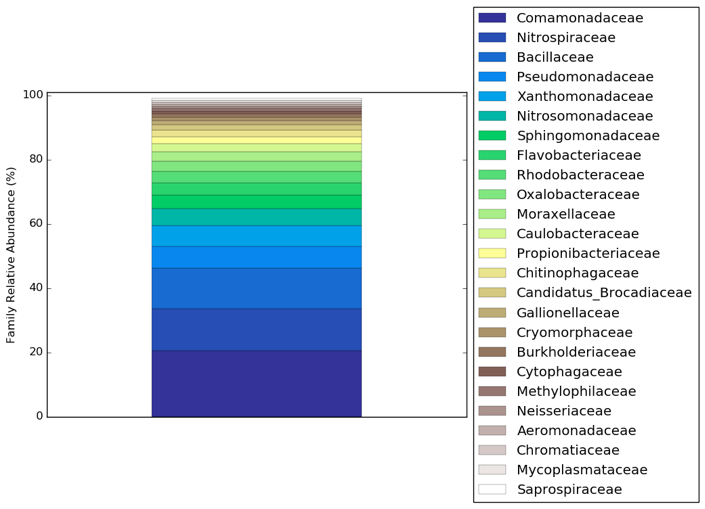

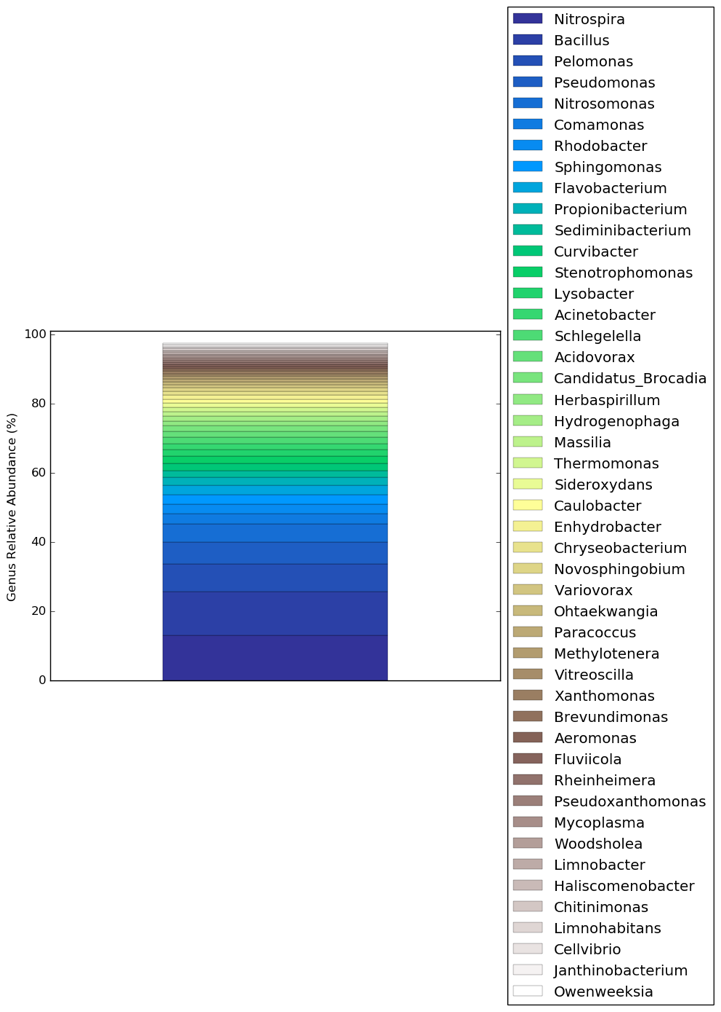


***Families Genera***

### **BP:** Phyla, Families, Genera


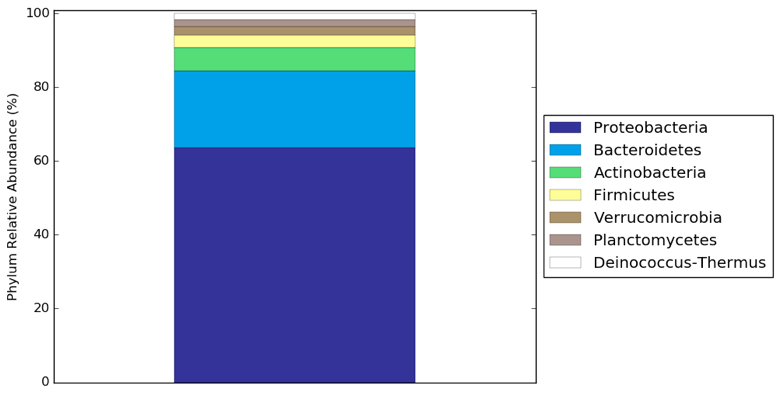

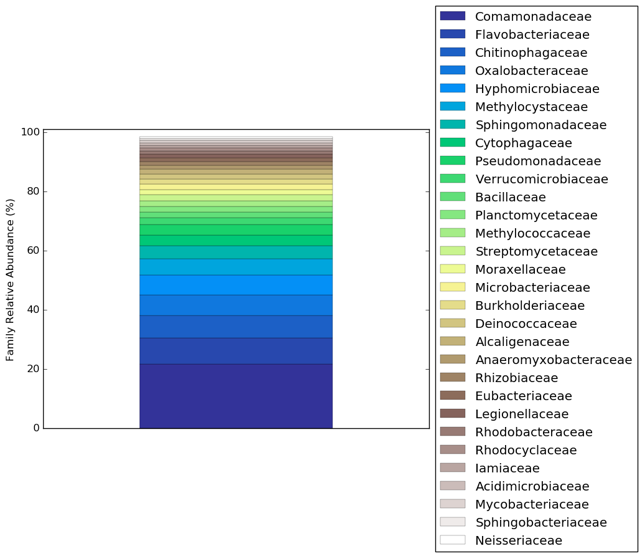


***Phyla Families***


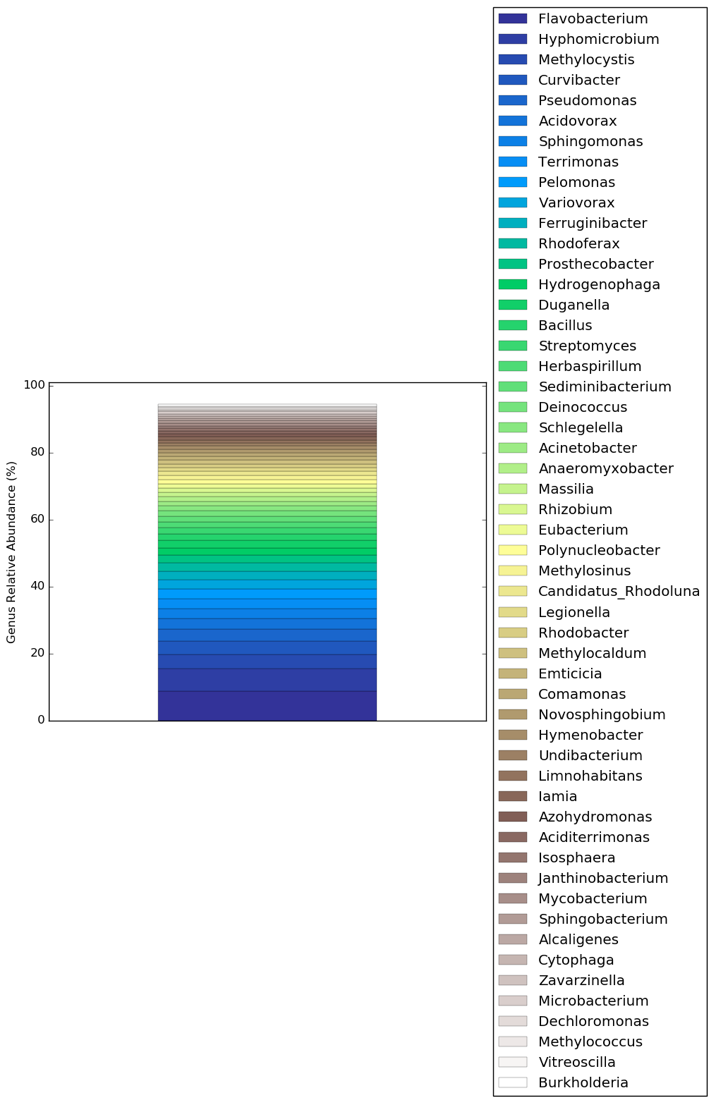


***Genera***

### **BW:** Phyla, Families, Genera


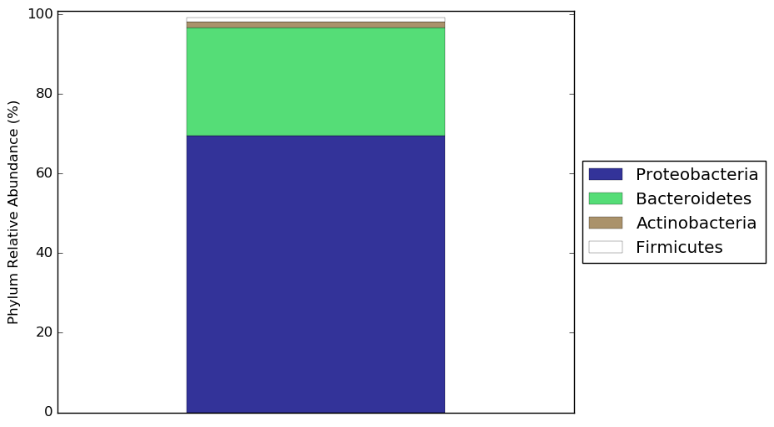

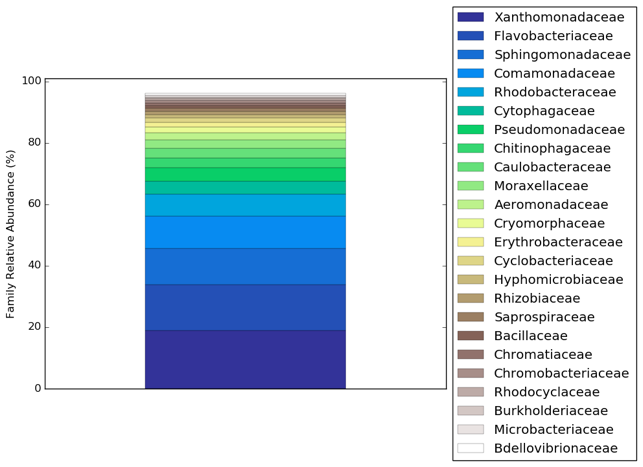


***Phyla Families***


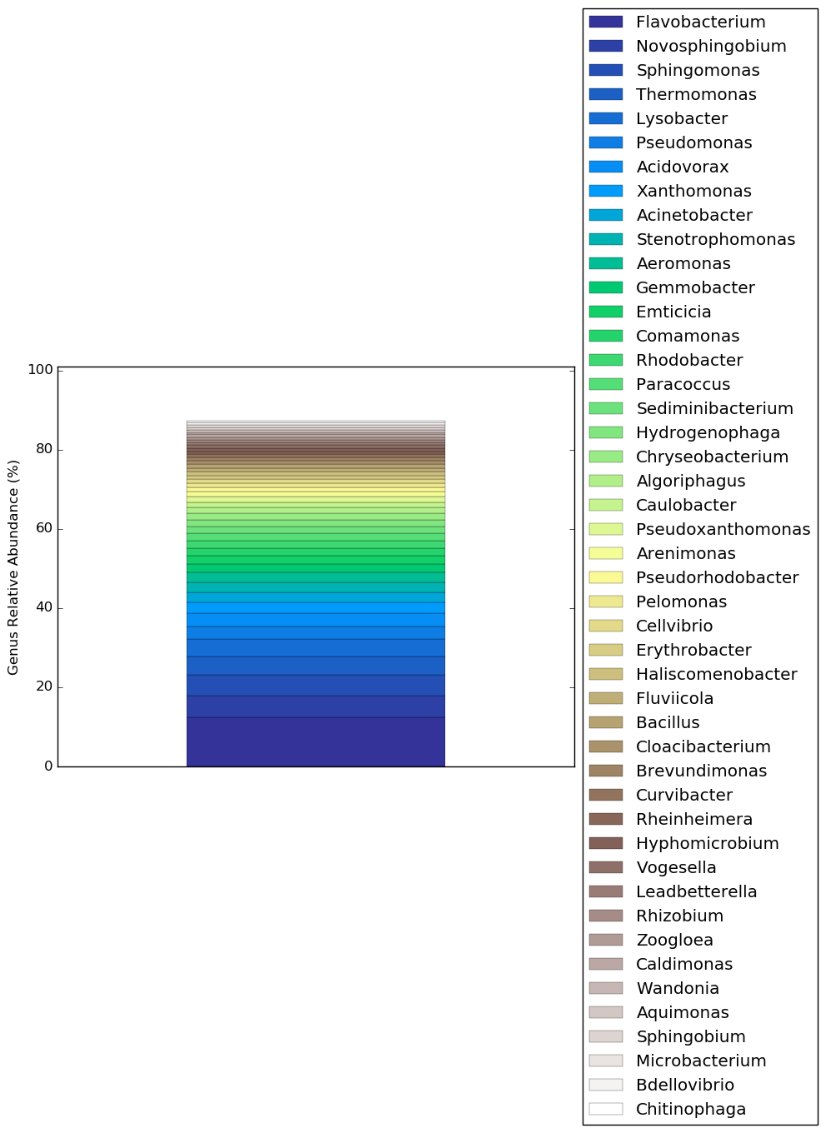


***Genera***

### **BVRHq:** Phyla, Families, Genera


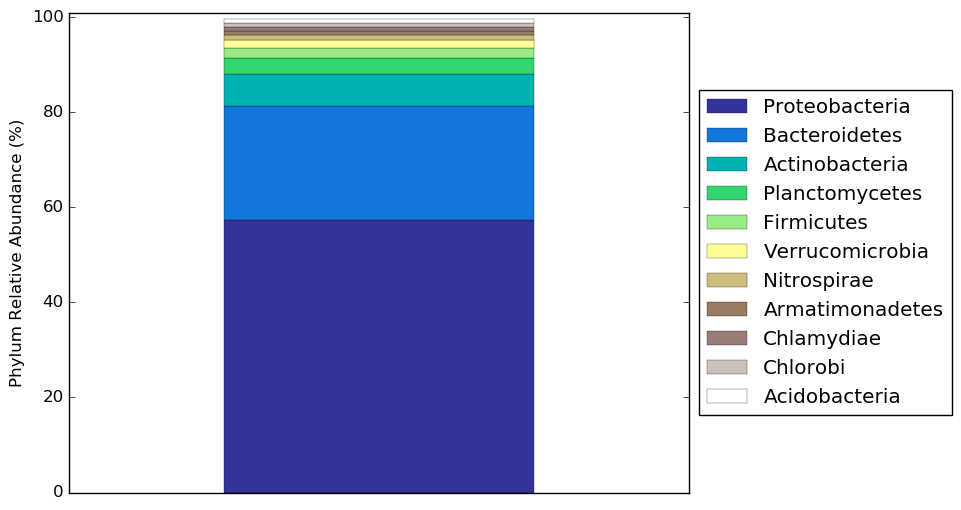


***Phyla***


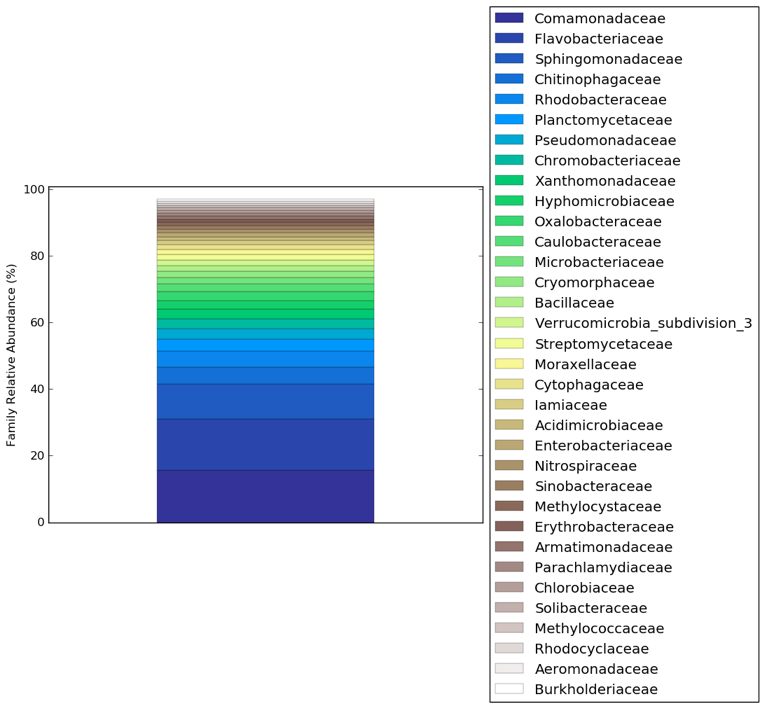

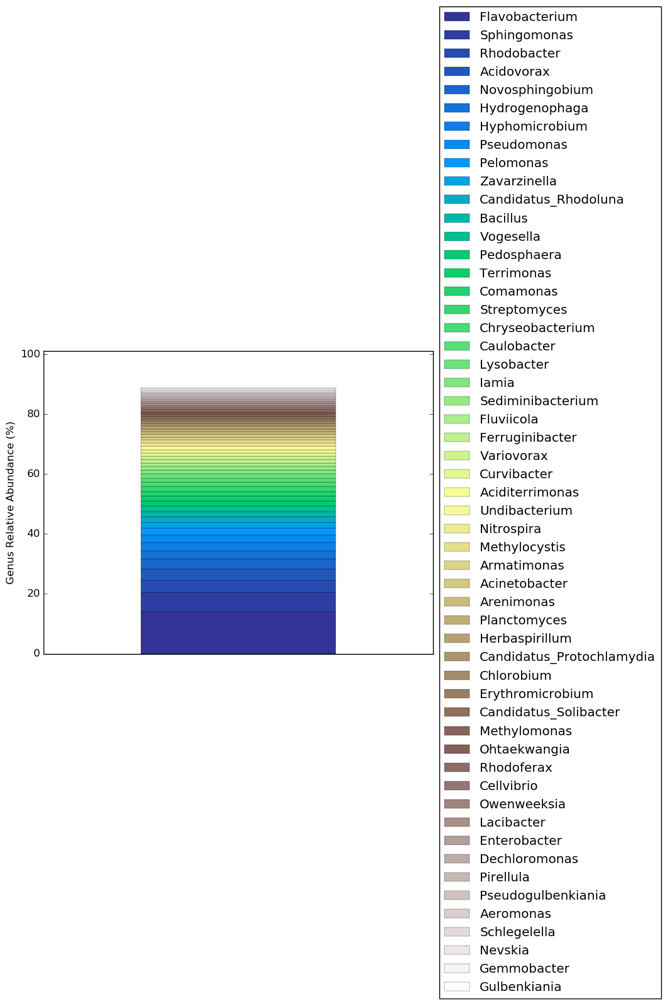


***Families Genera***

## MEPE

### **M. Pond:** Phyla, Families, Genera


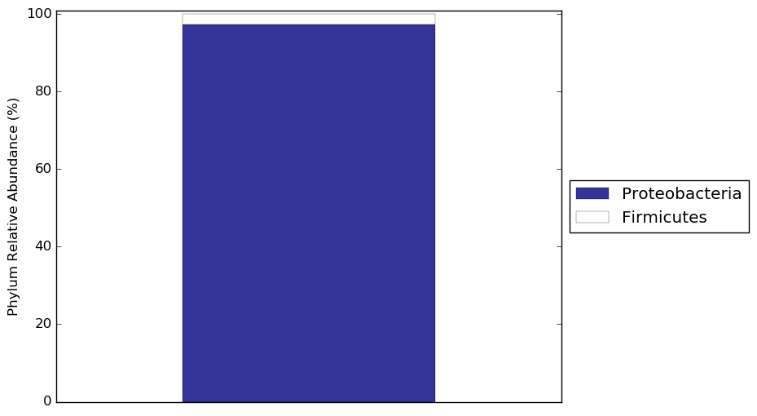


***Phyla***


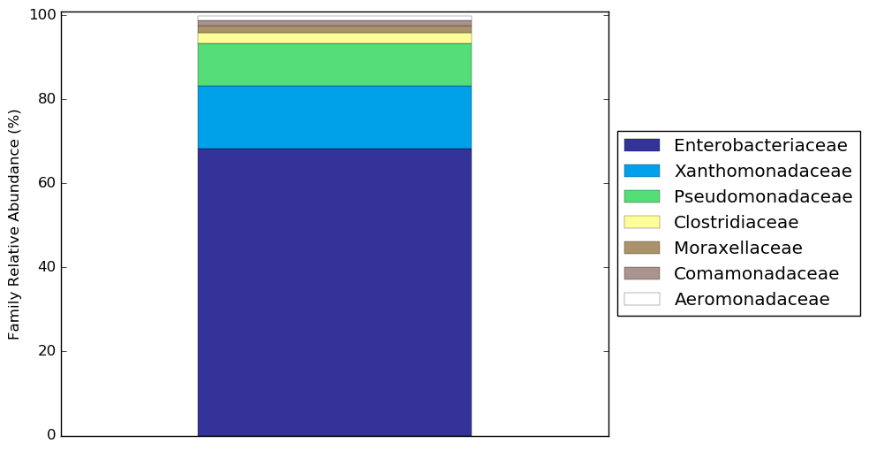


***Families***


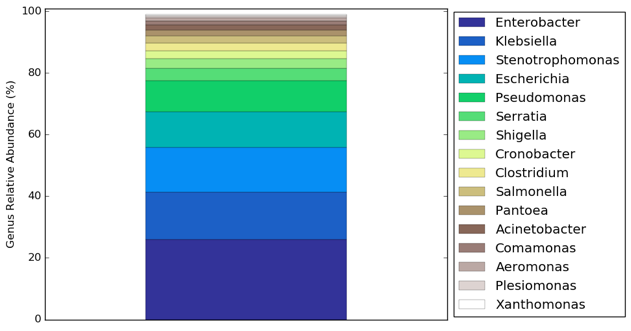


***Genera***

### **T. Bank:** Phyla, Families, Genera


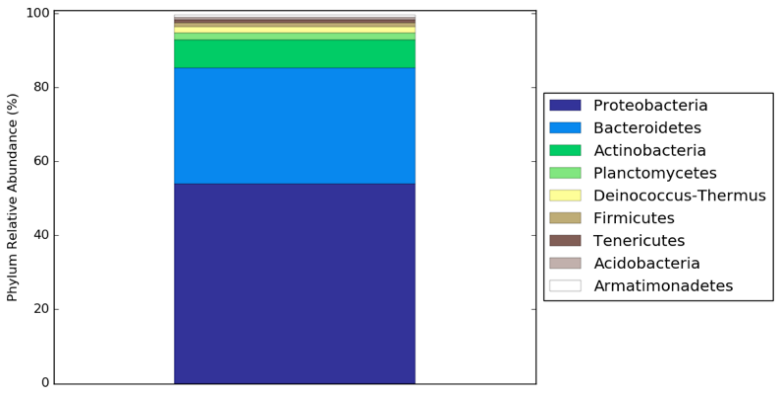


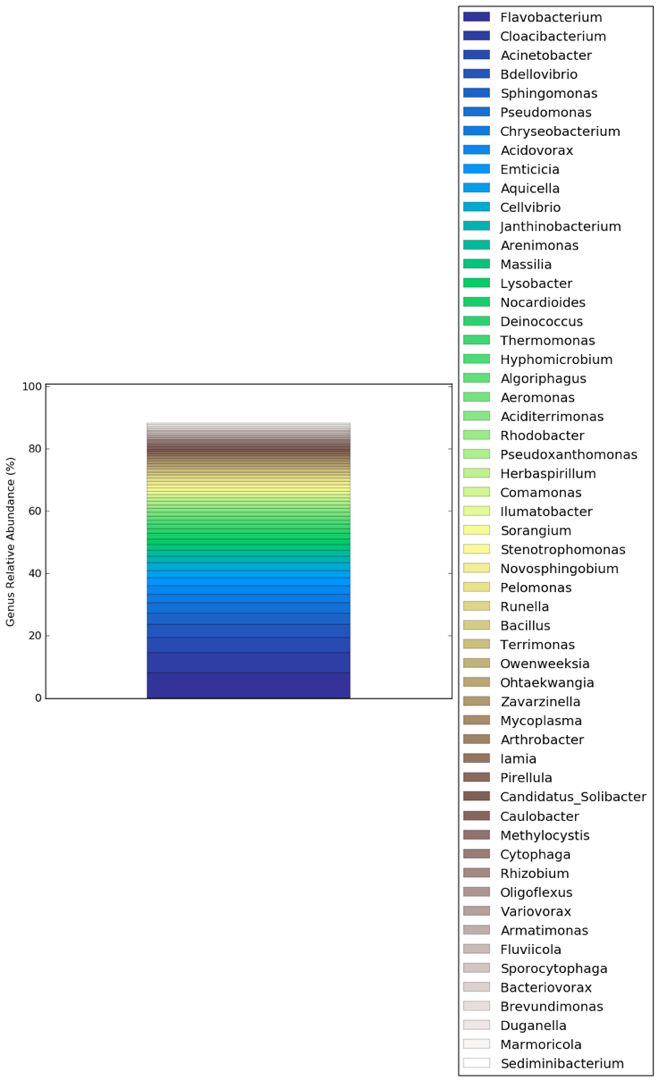


Phyla


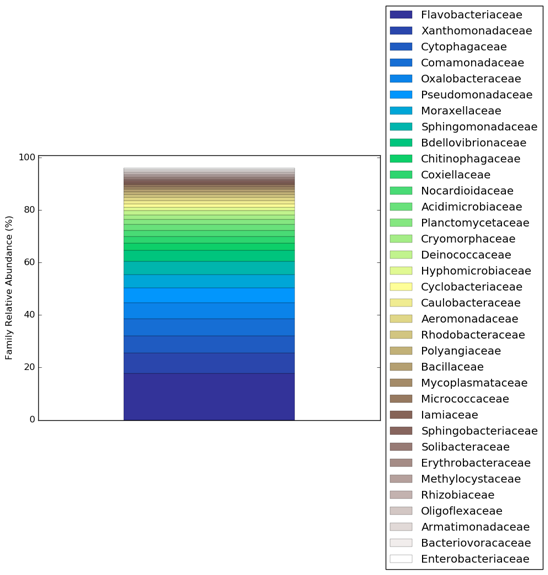


Families Genera

### **T. Middle:** Phyla, Families, Genera


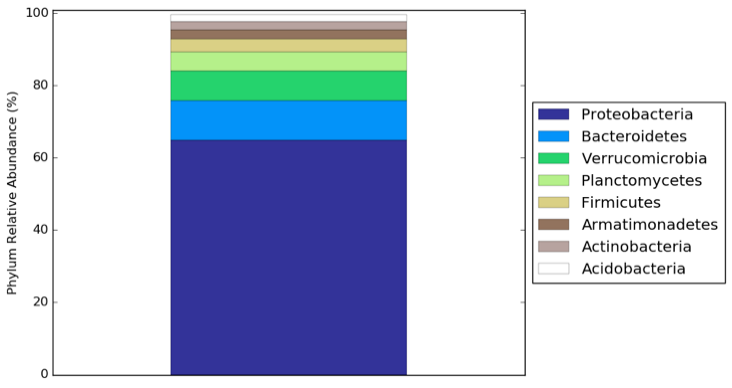


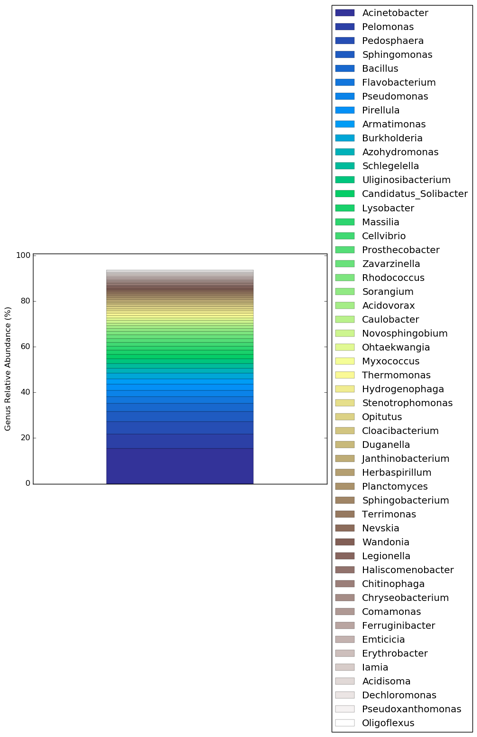


Phyla


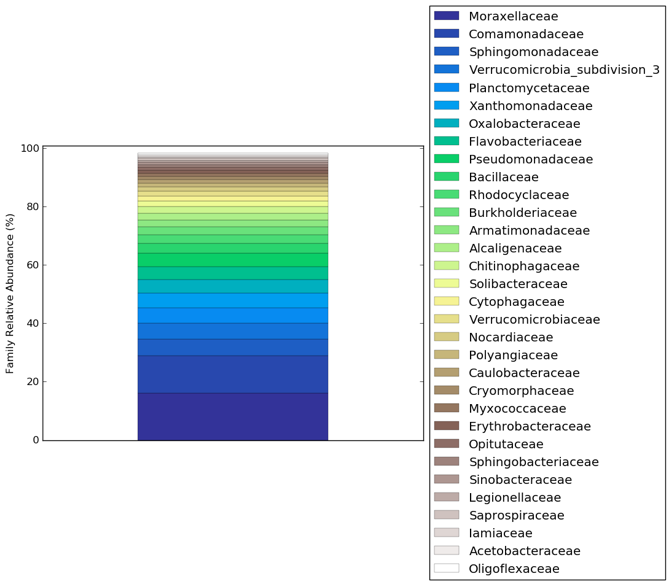


***Families Genera***

### **M. Bank:** Phyla, Families, Genera


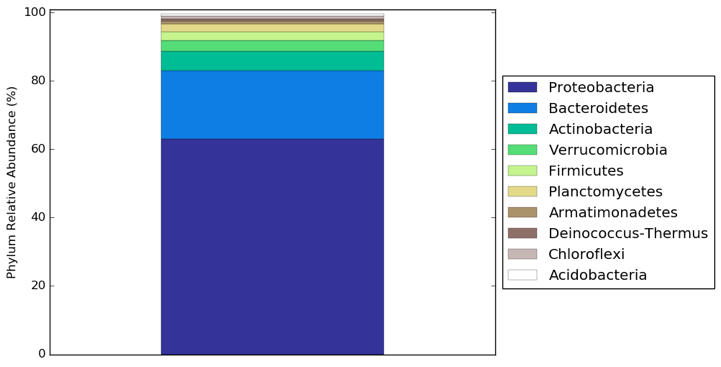


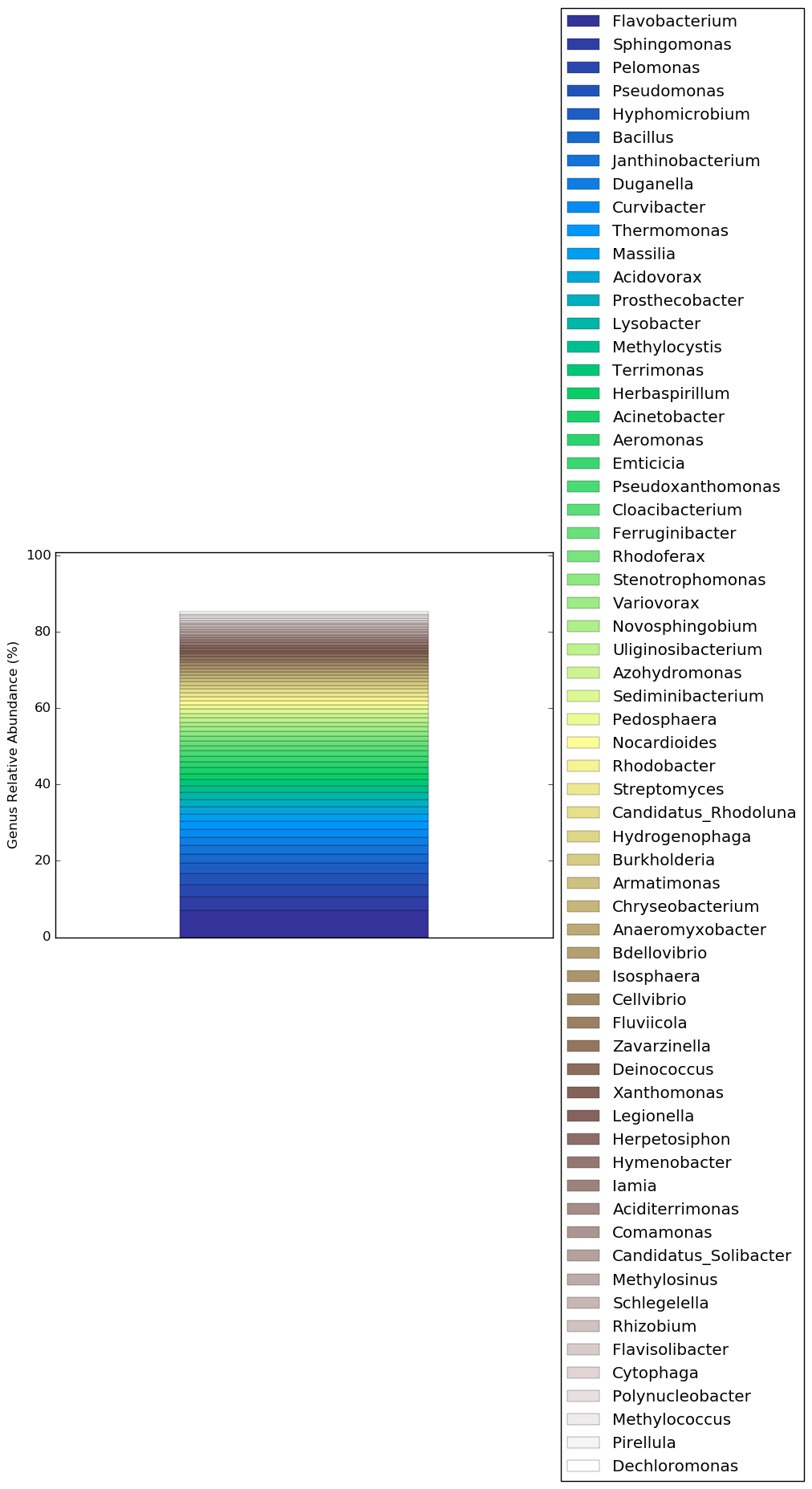


Phyla


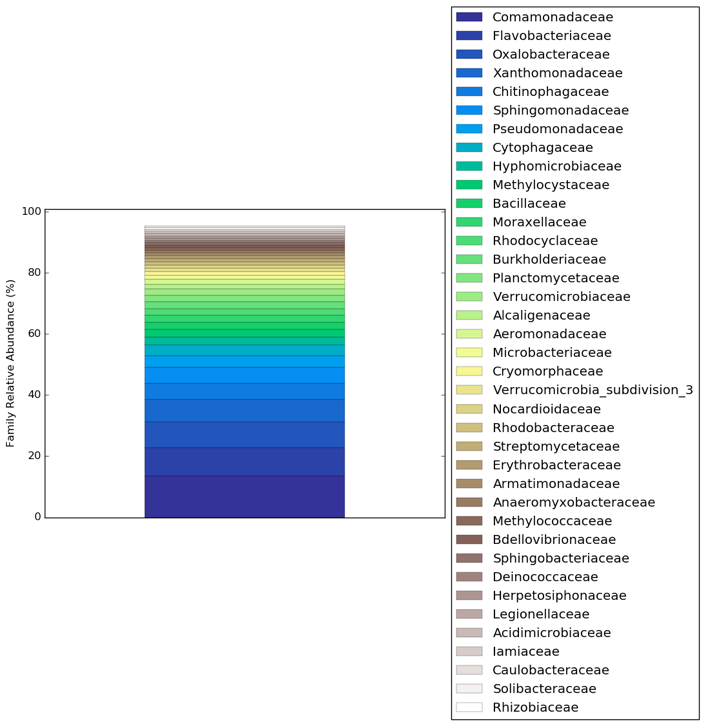


Families Genera

# KRONA PLOTS

## ASUTSUARE

### **ALB**


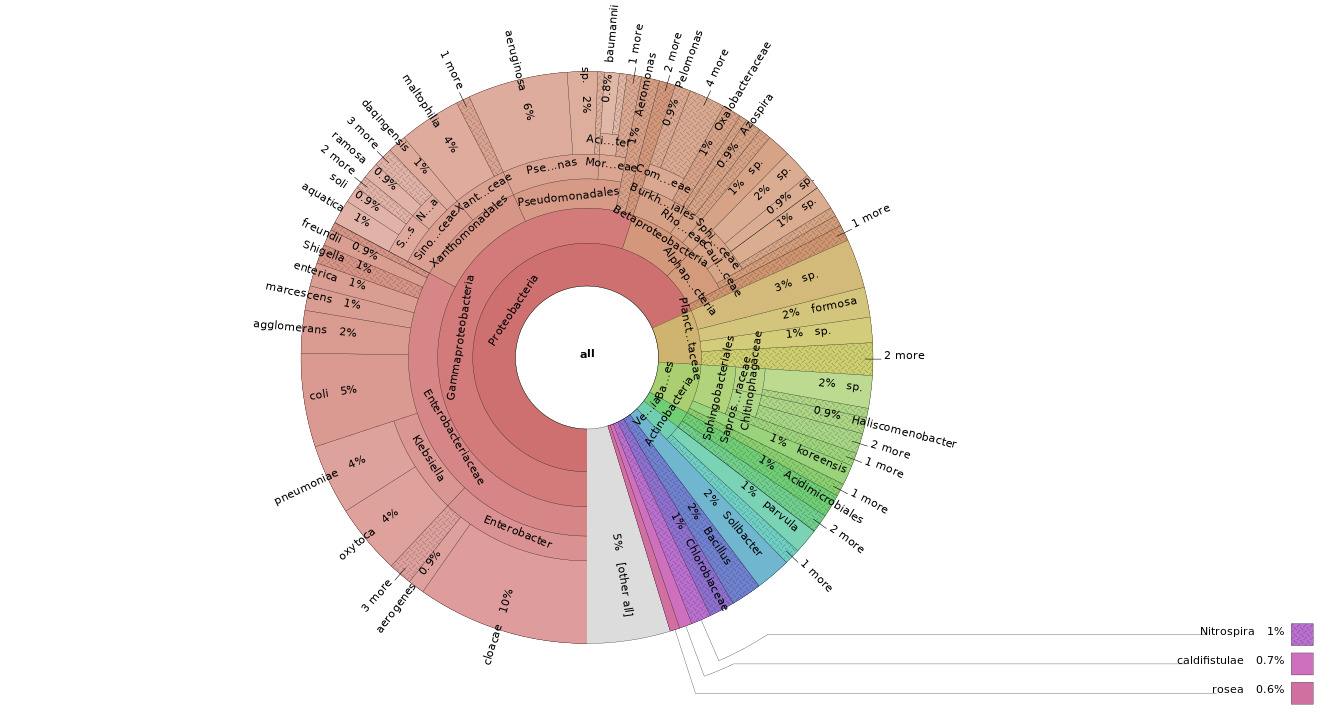


### **CD
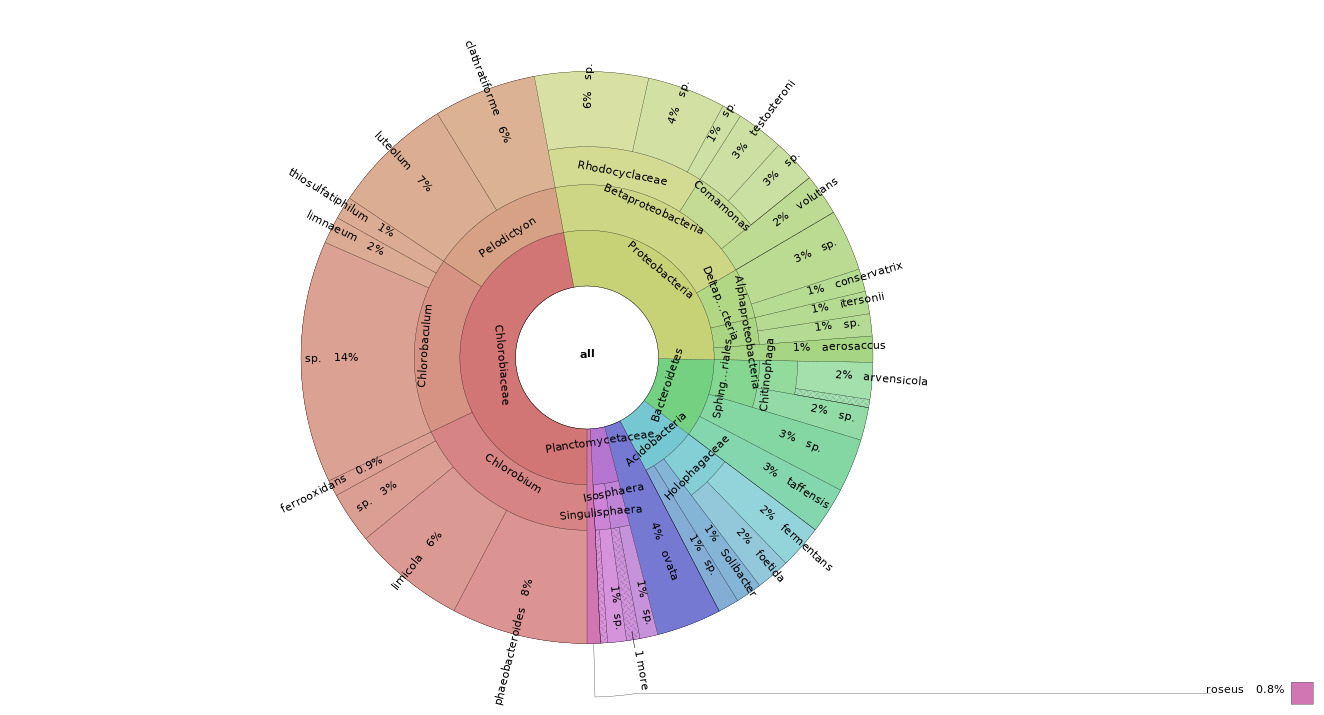
**

BATTOR

### **BVRm
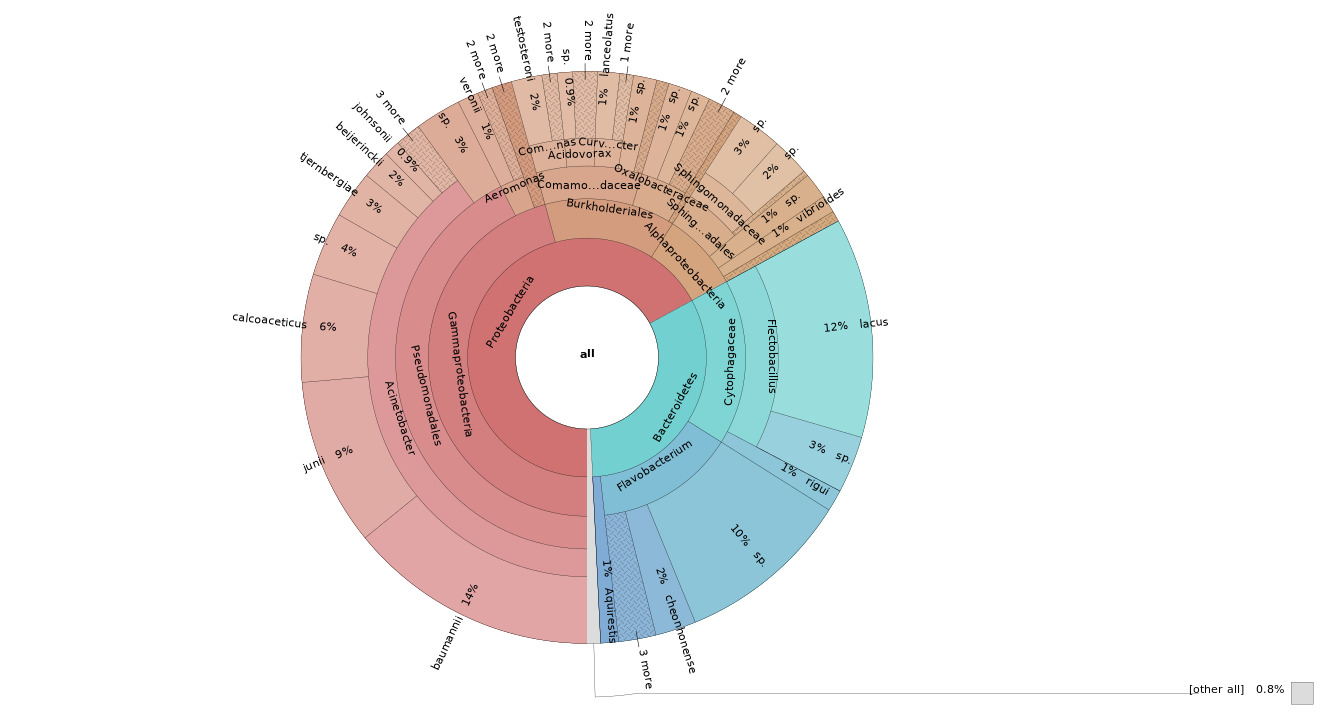
**

**BVR_0_**

*
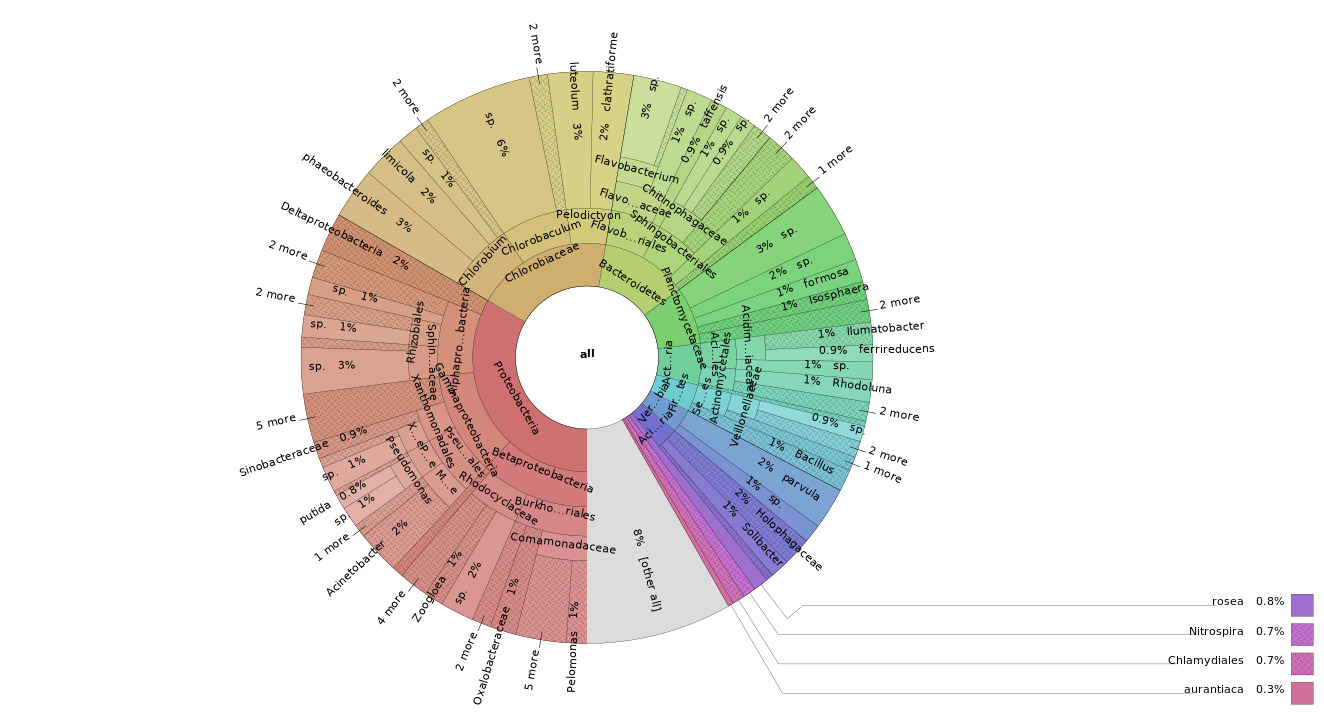
*

**BB**


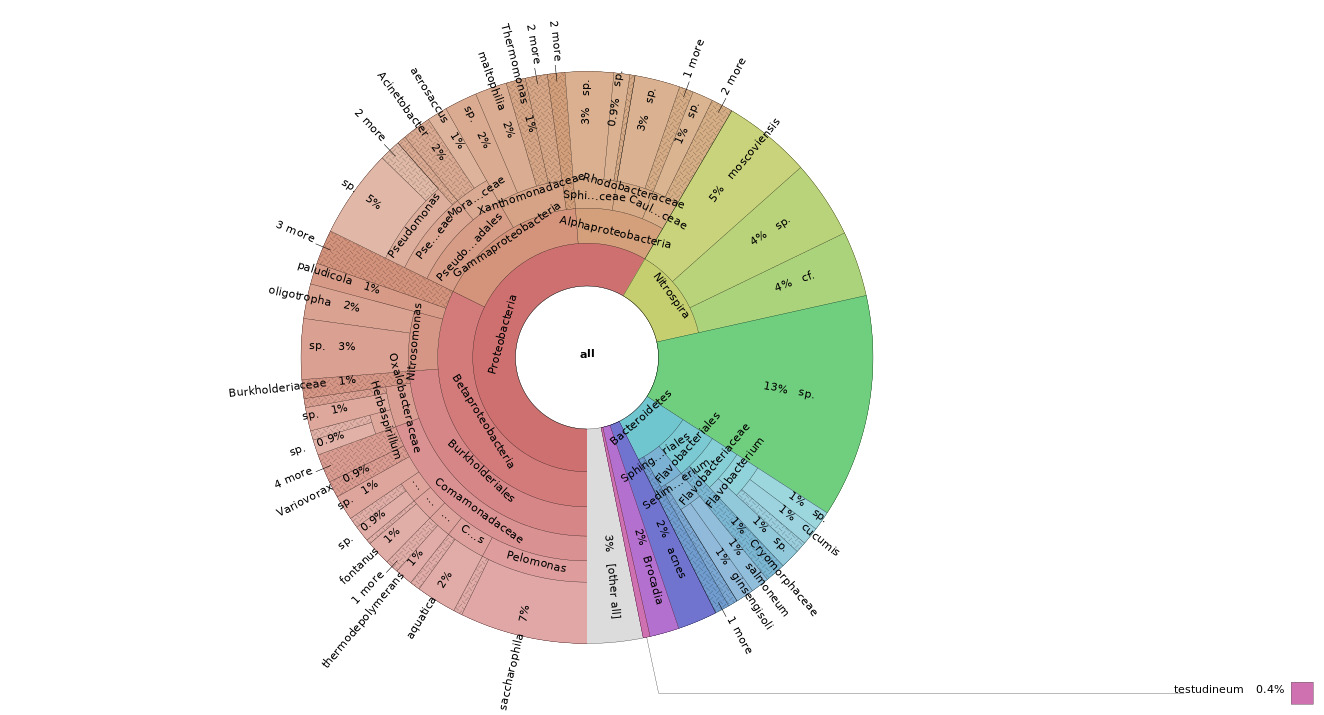


**B****P**


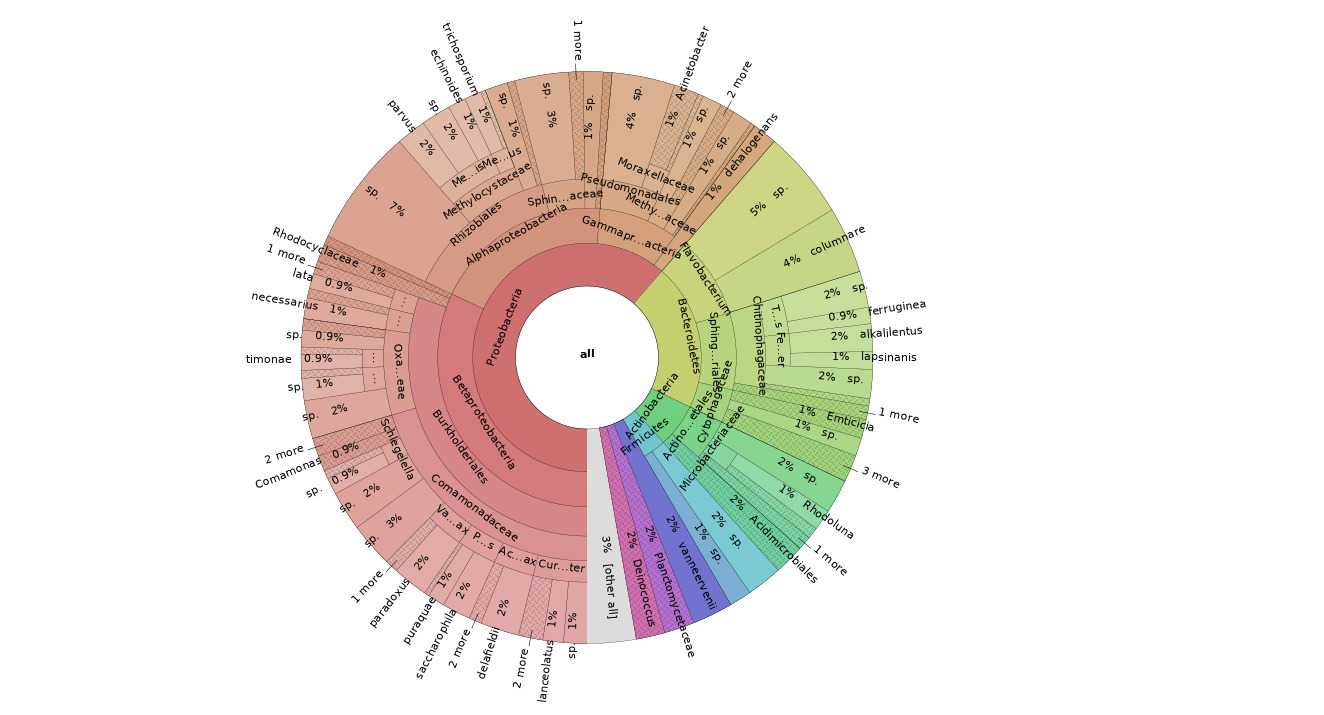


### **BW***
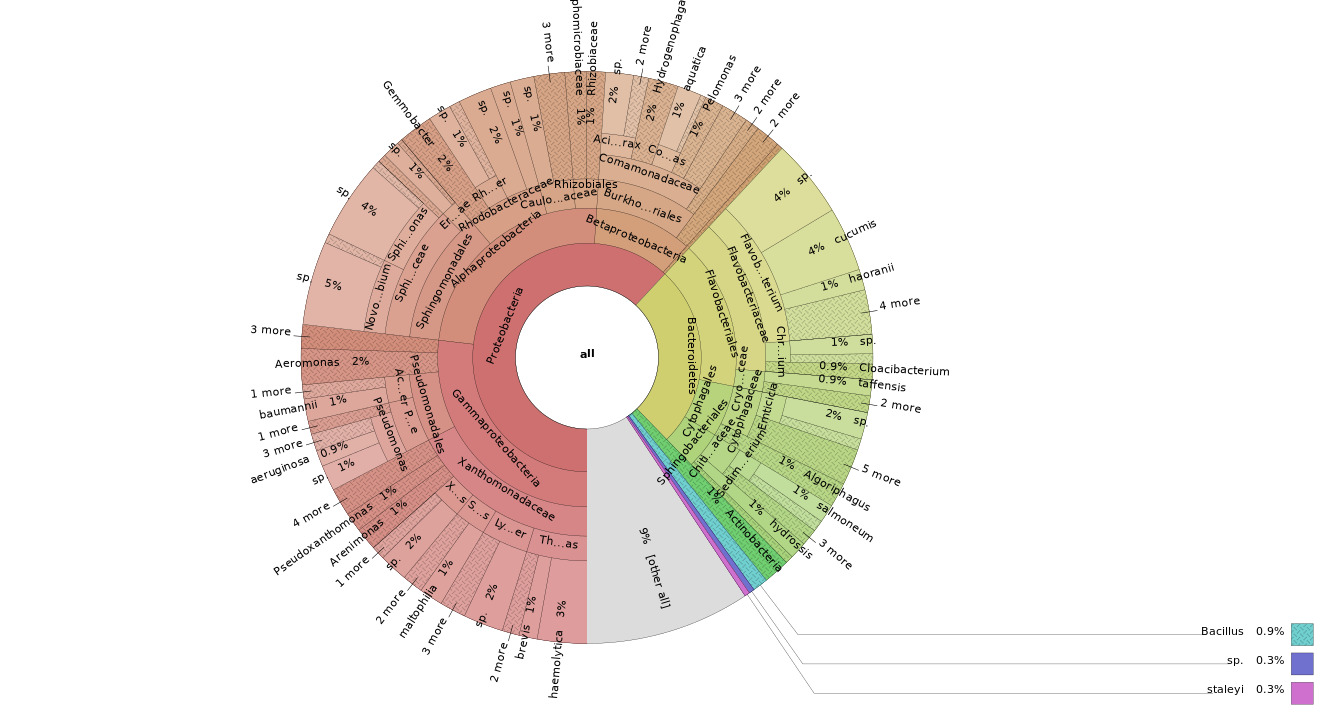
*

### **BVRHq**
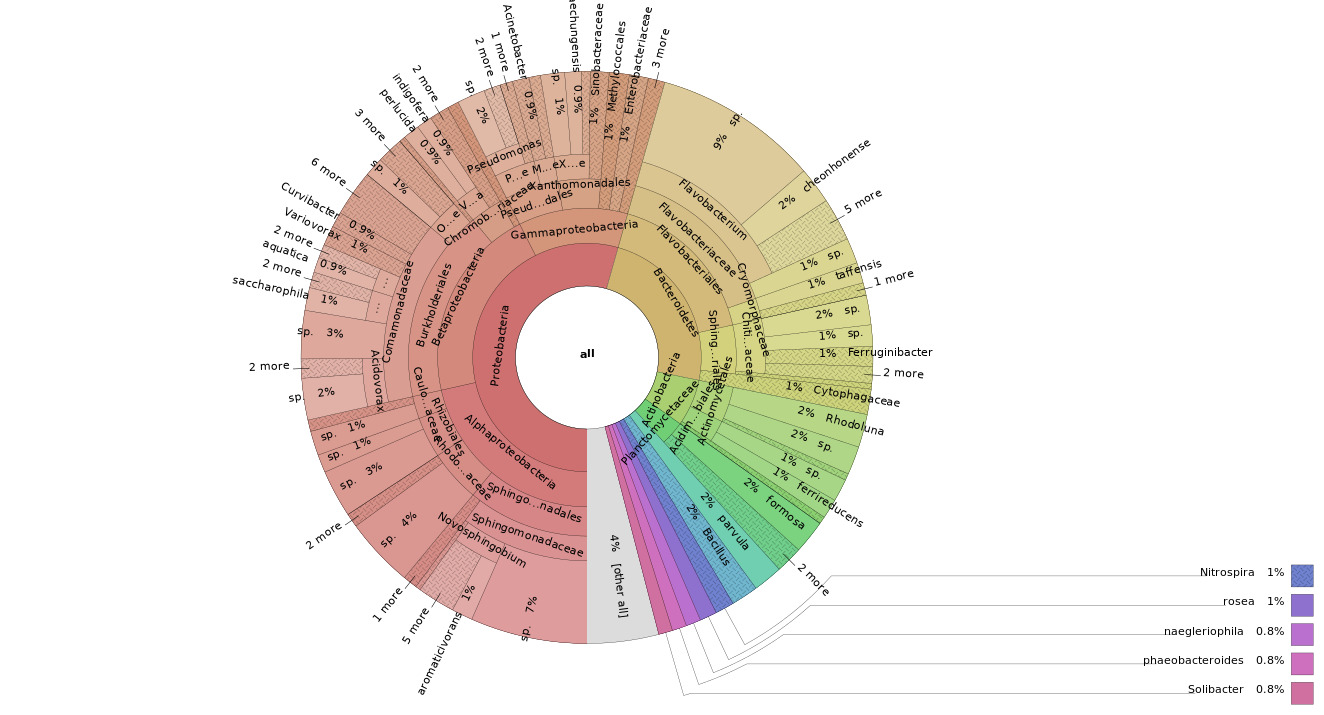


## MEPE

### **M. Pond**


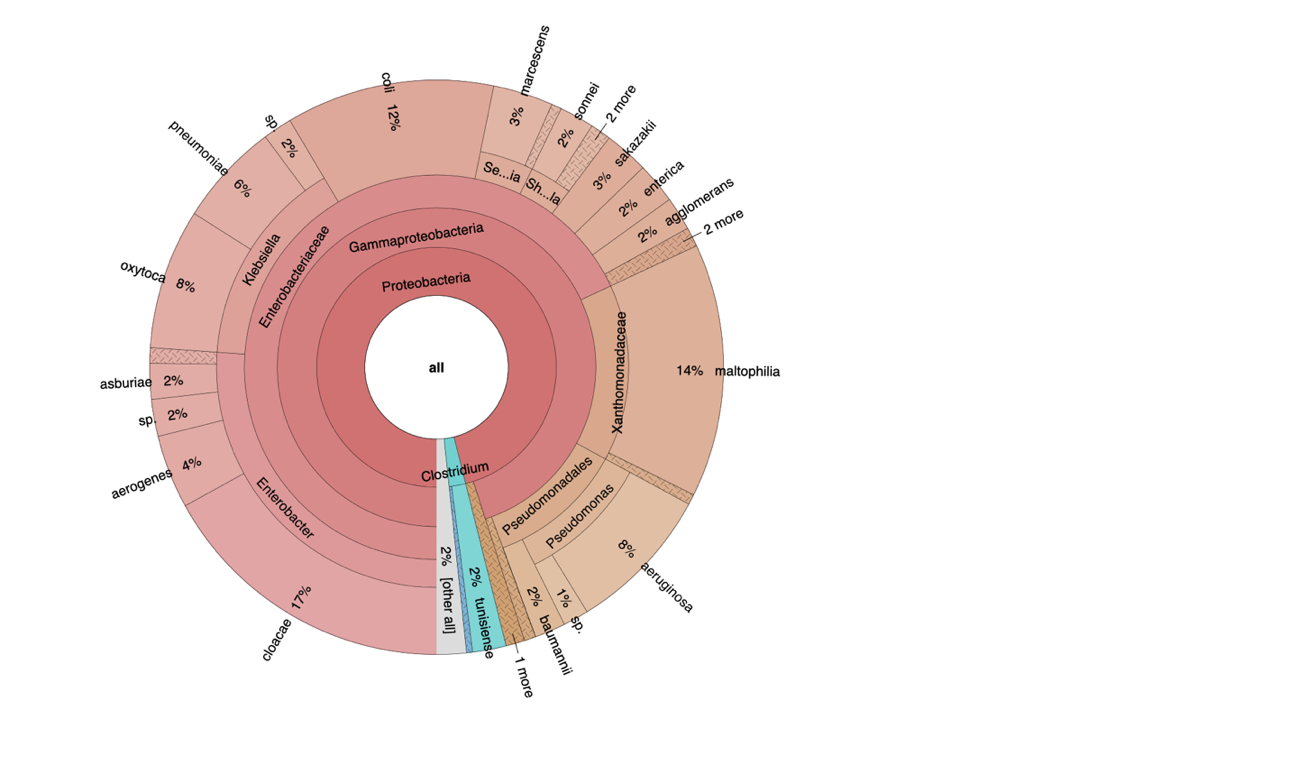


### **T. Bank**


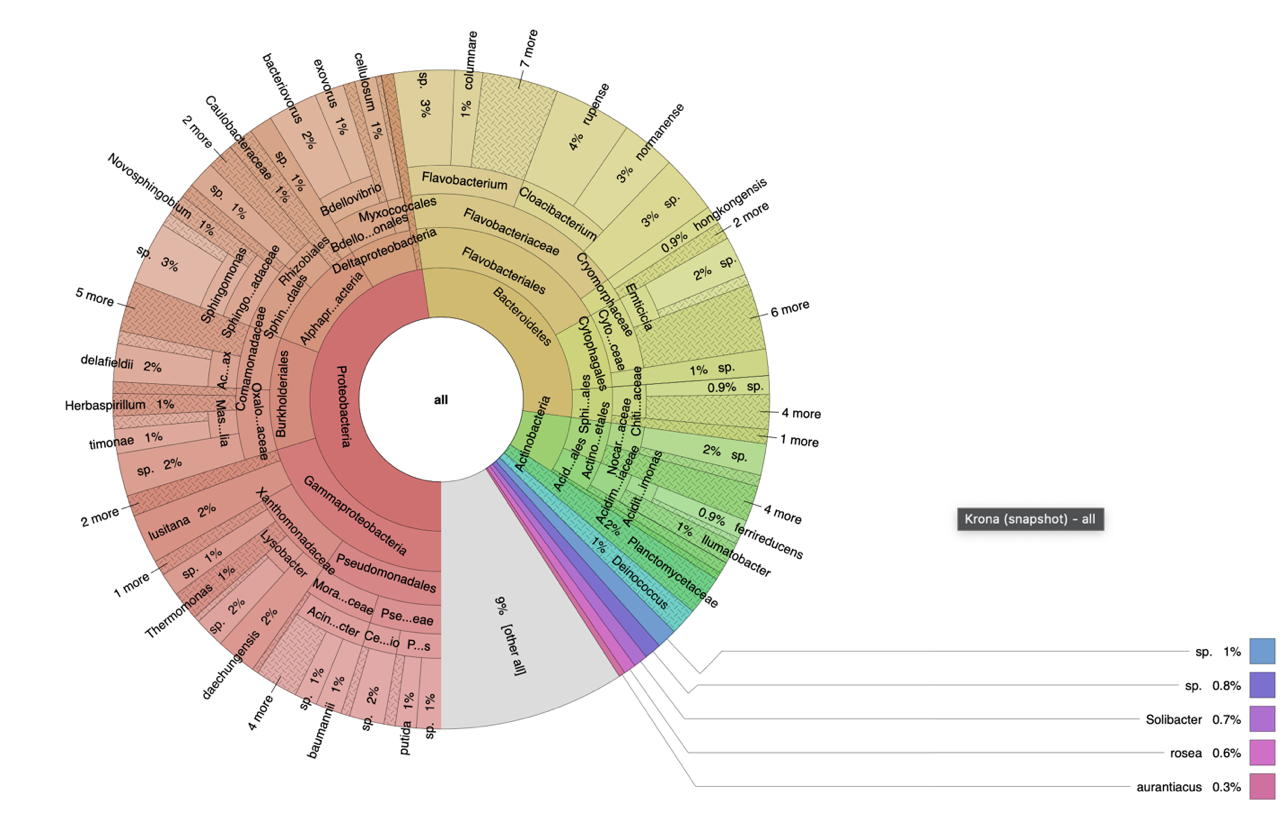


### **T. Middle**


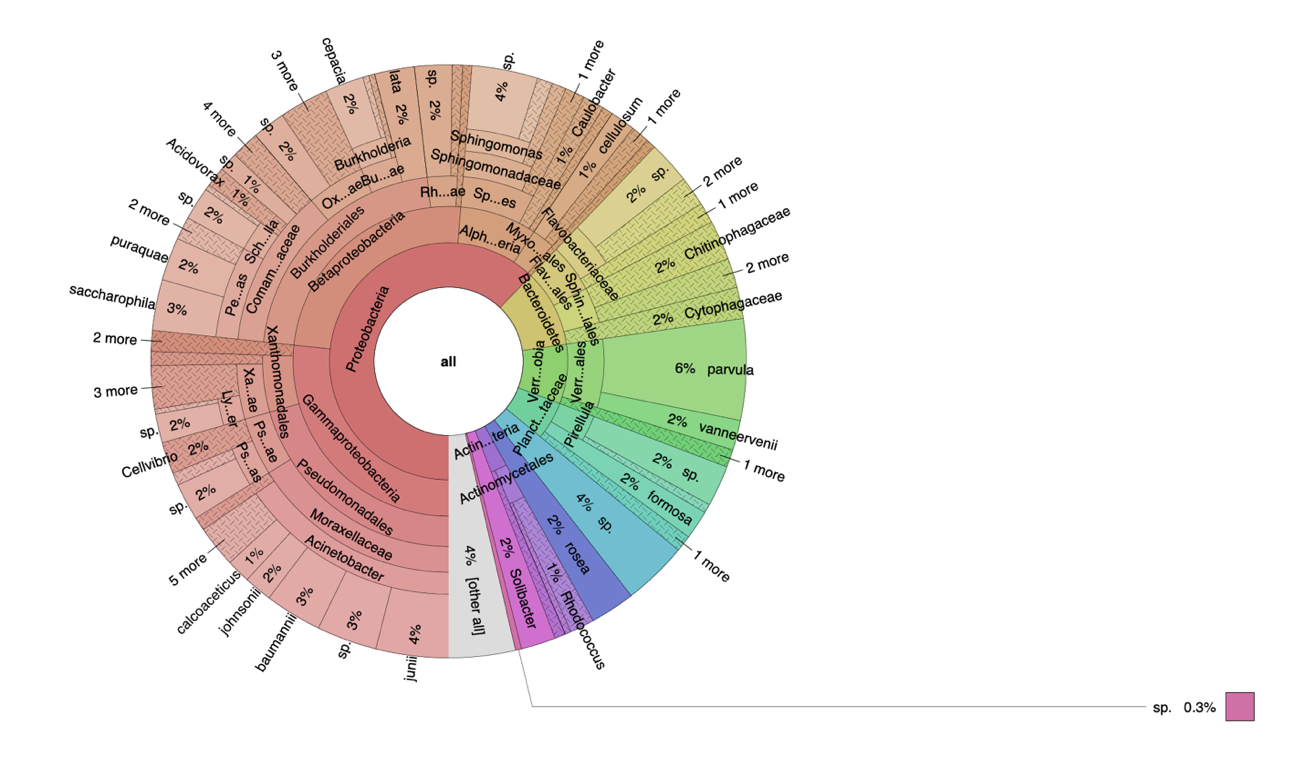


### **M. Bank**


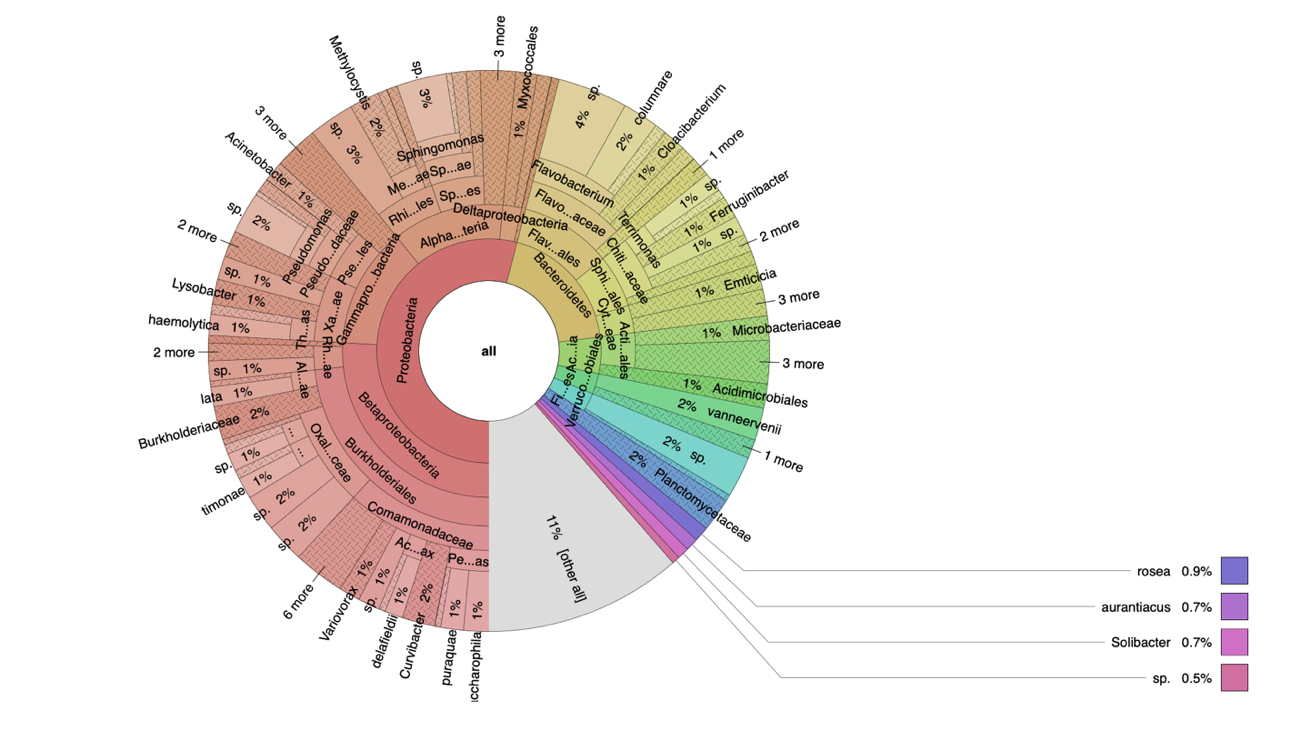

Supplement: S1 Fig — Graphical summaries of microbial relative abundance and community composition across sampling sites, including Krona plots illustrating the taxonomic distribution of microbial communities detected in each sample. (DOCX) [file pone.0346766.s001.docx]
