# Supplementary material for: Downstream community risks post dam-spillage flooding along the Volta River in Ghana: potential pathogens and public health implications
Source: PLoS One. 2026 Jul 2;21(7):e0346766. doi: 10.1371/journal.pone.0346766 (PMC13327315; doi:10.1371/journal.pone.0346766)

# Diversity Indices

## Alpha diversity indices

|  | Sample_ID | Chao1_Average | Shannon_Average | Simpson_Average |
| --- | --- | --- | --- | --- |
| Asutsuare | CD | 25.0 | 3.979 | 0.908 |
|  | CANAL | NA | NA | NA |
|  | ASB | NA | NA | NA |
|  | ASM | NA | NA | NA |
|  | ALB | 54.0 | 5.137 | 0.955 |
|  | | | | |
| Aveyime | AV. B | 69.0 | 5.814 | 0.977 |
|  | AV. M | 41.0 | 4.826 | 0.95 |
|  | AV. P | 65.0 | 5.381 | 0.952 |
|  | AV. A | 29.0 | 4.655 | 0.956 |
|  | AV. 1 | 65.0 | 5.729 | 0.977 |
|  | | | | |
| Battor | BVRf | NA | NA | NA |
|  | BVRm | 22.0 | 3.055 | 0.783 |
|  | BVRo | 61.0 | 5.510 | 0.969 |
|  | BB | 47.0 | 4.799 | 0.942 |
|  | BP | 53.0 | 5.350 | 0.968 |
|  | BW | 46.0 | 5.005 | 0.954 |
|  | BVRHq | 54.0 | 5.196 | 0.954 |
|  | | | | |
| Mepe | M. Pond | 16.0 | 3.212 | 0.856 |
|  | T. Bank | 56.0 | 5.370 | 0.967 |
|  | T. Middle | 52.0 | 5.094 | 0.951 |
|  | M. Bank | 63.0 | 5.701 | 0.976 |
|  | M. Well | NA | NA | NA |

# Alpha Diversity Plots:

ASUTSUARE

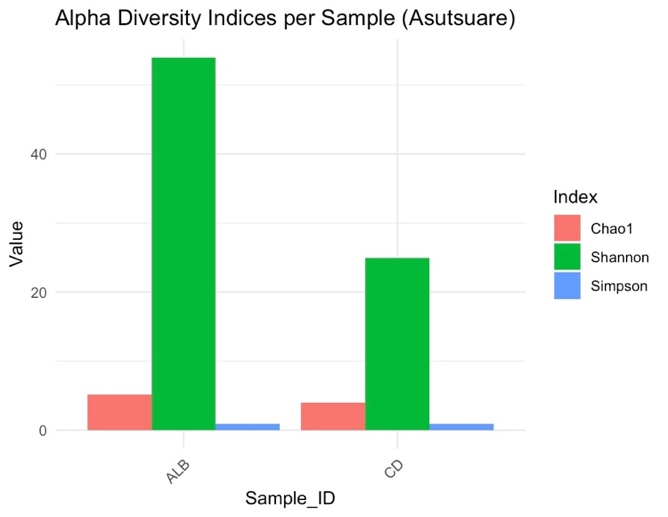


BATTOR


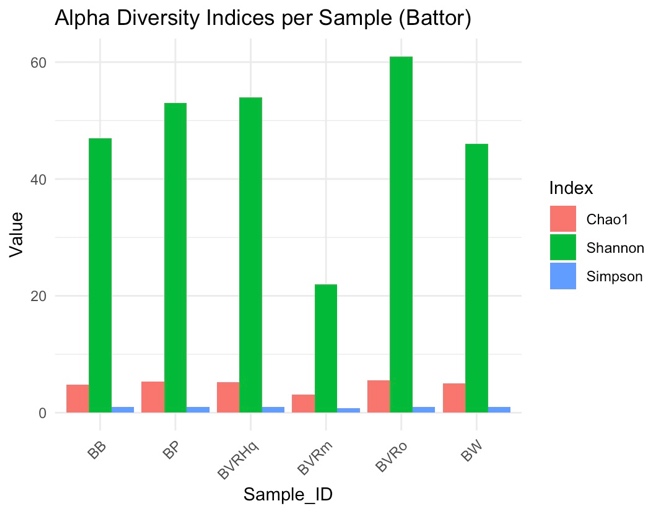


## AVEYIME


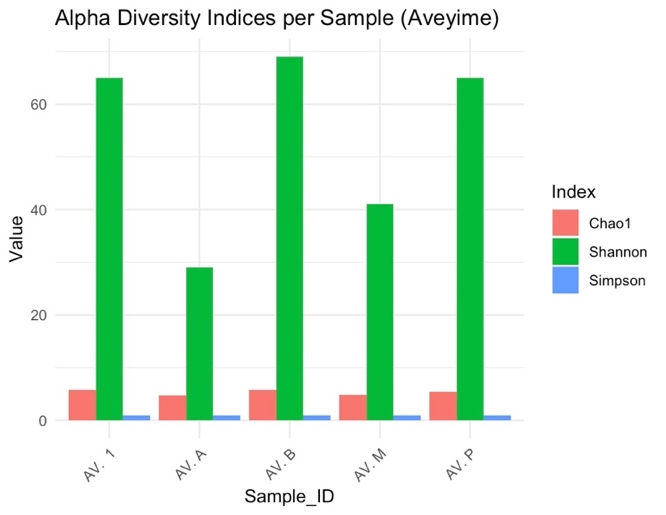


## MEPE


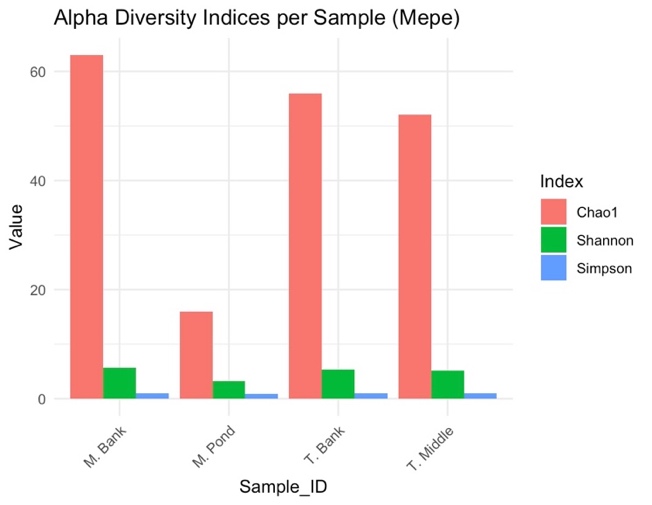

Supplement: S2 Fig — Tabulated alpha diversity indices and associated graphical representations of within-sample microbial diversity across all sampling sites and townships. (DOCX) [file pone.0346766.s002.docx]
