# Supplementary material for: Downstream community risks post dam-spillage flooding along the Volta River in Ghana: potential pathogens and public health implications
Source: PLoS One. 2026 Jul 2;21(7):e0346766. doi: 10.1371/journal.pone.0346766 (PMC13327315; doi:10.1371/journal.pone.0346766)
Supplement: S1 Table — Geographic coordinates recorded for each sampling site across the four study townships (Asutsuare, Aveyime, Battor, and Mepe) using a handheld GPS device. (DOCX) [file pone.0346766.s003.docx]

# GPS Co-ordinates of Sampling Sites

| **Township** | **Sample** | **Coordinates (Lat; Long)** |
| --- | --- | --- |
| Asutsuare | CD | (6.0925375; 0.1953055) |
|  | CANAL | (6.094400; 0.200230) |
|  | ASB | (6.095900; 0.198970) |
|  | ASM | (6.099601; 0.199400) |
|  | ALB | (6.101300; 0.202100) |
|  | | |
| Aveyime | AV. B | (6.040906; 0.383660) |
|  | AV. M | (6.043314; 0.382985) |
|  | AV. P | (6.0412569; 0.4066138) |
|  | AV. A | (6.0759272; 0.4011928) |
|  | AV. 1 | (6.0751534; 0.4025406) |
|  | | |
| Battor | BVRf | (6.0742832; 0.3993068) |
|  | BVRm | (6.0768473; 0.3991177) |
|  | BVRo | (6.0787410; 0.3987097) |
|  | BB | (6.0751812; 0.4096732) |
|  | BP | (6.0403; 0.40829) |
|  | BW | (6.0731143; 0.4092085) |
|  | BVRHq | (6.0785776; 0.4085571) |
|  | | |
| Mepe | M. Pond | (6.0796; 0.45879) |
|  | T. Bank | (6.083812; 0.4400244) |
|  | T. Middle | (6.0828; 0.43762) |
|  | M. Bank | (6.0808337; 0.4356280) |
|  | M. Well | (6.07741; 0.43196) |
