# Supplementary material for: Downstream community risks post dam-spillage flooding along the Volta River in Ghana: potential pathogens and public health implications
Source: PLoS One. 2026 Jul 2;21(7):e0346766. doi: 10.1371/journal.pone.0346766 (PMC13327315; doi:10.1371/journal.pone.0346766)

PLOS One

Downstream community risks post dam-spillage flooding along the Volta River in Ghana: potential pathogens and public health implications

--Manuscript Draft--

**Manuscript Number:**	PONE-D-26-14434
**Article Type:**	Research Article
**Full Title:**	Downstream community risks post dam-spillage flooding along the Volta River in Ghana: potential pathogens and public health implications
**Short Title:**	Dam-Spillage Flooding and Pathogen Risks in Ghana
**Corresponding Author:**	GRACE SEMABIA KPELI, PhD<br>University of Health and Allied Sciences<br>Ho, GHANA
**Keywords:**	flooding, dam spillage, bacteria, 16S rRNA sequencing, Volta River, Escherichia coli, Salmonella enterica, Acinetobacter baumannii, Klebsiella pneumoniae, Pseudomonas aeruginosa, Stenotrophomonas maltophilia, microbial diversity, taxonomy, healthcare-assoc
**Abstract:**	Flooding compromises the microbiological quality of domestic and recreational water sources, increasing exposure to waterborne and opportunistic pathogens in affected communities. Following widespread flooding triggered by the September 2023 controlled spillage of the Akosombo and Kpong dams in Ghana, which led to massive flooding, we assessed the microbial quality of water bodies in communities downstream of the Volta River and associated public and ecosystem health implications. In July 2024, water was collected from multiple source types across four downstream townships (Asutsuare, Aveyime, Battor, and Mepe), including river sections, ponds, puddles, wells, boreholes, and canal-associated sites. Samples were filtered, microbial DNA was extracted, and bacterial community composition was profiled using 16S rRNA gene sequencing, with downstream bioinformatics analyses used to characterize microbial diversity, assess spatial variation across sites, and stratify detected taxa according to ecological origin and public health risk. Sequence data obtained from 17 out of 22 samples revealed communities dominated by Proteobacteria with frequent co-occurrence of Bacteroidetes and Firmicutes. Across all sites, 56 microbial taxa and 118 microorganisms were detected. Community composition varied by location and water source type, with bank and mid-river sites generally exhibiting higher richness than puddles and a canal drain. Thirteen samples contained site-specific taxa, indicating marked spatial heterogeneity within and between communities. Microorganisms of public health relevance were frequently detected, including enteric-associated bacteria such as Escherichia coli, Salmonella enterica, Shigella species, and Cronobacter sakazakii, as well as opportunistic and healthcare-associated bacteria including Acinetobacter baumannii, Klebsiella pneumoniae, Pseudomonas aeruginosa, and Stenotrophomonas maltophilia. Higher-risk organisms were most widely distributed in Mepe and Battor, including in groundwater-associated sources. These findings provide molecular evidence of persistent, spatially heterogeneous microbial hazards months after flooding, underscoring continued exposure risks in downstream communities and the need for targeted water safety interventions and risk-informed public health responses.
**Order of Authors:**	GRACE SEMABIA KPELI, PhD
	Prince Agyirey-Kwakye
	Counseller Nutifafa Livingstone
	Lillian Teye Cudjoe
	Emmanuel Edem Dotse
	Ebenezer Nyarko
	Ebenezer Zar
	Obed Nyarko-Otu

Powered by Editorial Manager® and ProduXion Manager® from Aries Systems Corporation

	Hubert Kwame Agbogli
	Godwin Glilekpe
	Solomon Korankye
	Priscilla Essandoh
	Daniel Elorm Kabotso
**Additional Information:**	
**Question**	**Response**
**Financial Disclosure**<br><br>Enter a financial disclosure statement that describes the sources of funding for the work included in this submission. Review the submission guidelines for detailed requirements. View published research articles from *PLOS ONE* for specific examples.<br><br>This statement is required for submission and **will appear in the published article** if the submission is accepted. Please make sure it is accurate.<br><br>**Funded studies**<br>Enter a statement with the following details:<br>• Initials of the authors who received each award<br>• Grant numbers awarded to each author<br>• The full name of each funder<br>• URL of each funder website<br>• Did the sponsors or funders play any role in the study design, data collection and analysis, decision to publish, or preparation of the manuscript?<br><br>Did you receive funding for this work?	The author(s) received no specific funding for this work.
**Competing Interests**<br><br>Use the instructions below to enter a competing interest statement for this submission. On behalf of all authors, disclose any competing interests that could be perceived to bias this work—acknowledging all financial support and any other relevant financial or non-financial competing interests.	The authors have declared that no competing interests exist.

This statement is required for submission and **will appear in the published article** if the submission is accepted. Please make sure it is accurate and that any funding sources listed in your Funding Information later in the submission form are also declared in your Financial Disclosure statement.<br><br>View published research articles from *PLOS ONE* for specific examples.<br><br>**NO authors have competing interests**<br><br>Enter: *The authors have declared that no competing interests exist.*<br><br>**Authors with competing interests**<br><br>Enter competing interest details beginning with this statement:<br><br>*I have read the journal's policy and the authors of this manuscript have the following competing interests: [insert competing interests here]*<br><br>* typeset	
**Ethics Statement**<br><br>Enter an ethics statement for this submission. This statement is required if the study involved:<br><br>• Human participants<br>• Human specimens or tissue<br>• Vertebrate animals or cephalopods<br>• Vertebrate embryos or tissues<br>• Field research<br><br>Write "N/A" if the submission does not require an ethics statement.<br><br>General guidance is provided below. Consult the submission guidelines for detailed instructions. **Make sure that all information entered here is included in the**	N/A

**Methods section of the manuscript.**<br><br>**Format for specific study types**<br><br>**Human Subject Research (involving human participants and/or tissue)**<br>• Give the name of the institutional review board or ethics committee that approved the study<br>• Include the approval number and/or a statement indicating approval of this research<br>• Indicate the form of consent obtained (written/oral) or the reason that consent was not obtained (e.g. the data were analyzed anonymously)<br><br>**Animal Research (involving vertebrate animals, embryos or tissues)**<br>• Provide the name of the Institutional Animal Care and Use Committee (IACUC) or other relevant ethics board that reviewed the study protocol, and indicate whether they approved this research or granted a formal waiver of ethical approval<br>• Include an approval number if one was obtained<br>• If the study involved *non-human primates*, add *additional details* about animal welfare and steps taken to ameliorate suffering<br>• If anesthesia, euthanasia, or any kind of animal sacrifice is part of the study, include briefly which substances and/or methods were applied<br><br>**Field Research**<br><br>Include the following details if this study involves the collection of plant, animal, or other materials from a natural setting:<br>• Field permit number<br>• Name of the institution or relevant body that granted permission	
**Data Availability**<br><br>Authors are required to make all data underlying the findings described fully available, without restriction, and from the time of publication. PLOS allows rare exceptions to address legal and ethical concerns. See the PLOS Data Policy and FAQ for detailed information.	Yes - all data are fully available without restriction

A Data Availability Statement describing where the data can be found is required at submission. Your answers to this question constitute the Data Availability Statement and **will be published in the article**, if accepted.<br><br>**Important:** Stating 'data available on request from the author' is not sufficient. If your data are only available upon request, select 'No' for the first question and explain your exceptional situation in the text box.<br><br>Do the authors confirm that all data underlying the findings described in their manuscript are fully available without restriction?	
**Describe where the data may be found in full sentences. If you are copying our sample text, replace any instances of XXX with the appropriate details.**<br><br>• If the data are **held or will be held in a public repository**, include URLs, accession numbers or DOIs. If this information will only be available after acceptance, indicate this by ticking the box below. For example: *All XXX files are available from the XXX database (accession number(s) XXX, XXX.*).<br>• If the data are all contained **within the manuscript and/or Supporting Information files**, enter the following: *All relevant data are within the manuscript and its Supporting Information files.*<br>• If neither of these applies but you are able to provide **details of access elsewhere**, with or without limitations, please do so. For example:<br><br>*Data cannot be shared publicly because of [XXX]. Data are available from the XXX Institutional Data Access / Ethics Committee (contact via XXX) for researchers who meet the criteria for access to confidential data.*<br><br>*The data underlying the results*	All relevant data are within the manuscript and its Supporting Information files.

presented in the study are available from (include the name of the third party and contact information or URL).<br>• This text is appropriate if the data are owned by a third party and authors do not have permission to share the data.<br><br>* typeset	
Additional data availability information:	Tick here if your circumstances are not covered by the questions above and you need the journal’s help to make your data available.

Manuscript
Click here to access/download;Manuscript;Downstream community risks post flooding Kpeli et al.docx

Downstream community risks post dam-spillage flooding along the Volta River in Ghana: potential pathogens and public health implications

Grace Semabia Kpeli[1*], Prince Agyirey-Kwakye[2], Counseller Nutifafa Livingstone[1], Lillian Teye Cudjoe[1], Emmanuel Edem Dotse[1], Ebenezer Nyarko[1], Ebenezer Zar[1], Obed Nyarko-Otu[1], Hubert Kwame Agbogli[1], Godwin Glilekpeh[1], Solomon Korankye[1], Priscilla Essandoh[1], Daniel Elorm Kabotso[3]

[1] Department of Biomedical Science, University of Health and Allied Sciences, Sokode Lokoe, Ho, Ghana

[2] AttoDiagnostics- UK

[3] Department of Basic Science, University of Health and Allied Sciences, Sokode Lokoe, Ho, Ghana

***Corresponding author**

E-mail: gkpeli@uhas.edu.gh (GSK)

Abstract

Flooding compromises the microbiological quality of domestic and recreational water sources, increasing exposure to waterborne and opportunistic pathogens in affected communities. Following widespread flooding triggered by the September 2023 controlled spillage of the Akosombo and Kpong dams in Ghana, which led to massive flooding, we assessed the microbial quality of water bodies in communities downstream of the Volta River and associated public and ecosystem health implications. In July 2024, water was collected from multiple source types across four downstream townships (Asutsuare, Aveyime, Battor, and Mepe), including river sections, ponds, puddles, wells, boreholes, and canal-associated sites. Samples were filtered, microbial DNA was extracted, and bacterial community composition was profiled using 16S rRNA gene sequencing, with downstream bioinformatics analyses used to characterize microbial diversity, assess spatial variation across sites, and stratify detected taxa according to ecological origin and public health risk. Sequence data obtained from 17 out of 22 samples revealed communities dominated by Proteobacteria with frequent co-occurrence of Bacteroidetes and Firmicutes. Across all sites, 56 microbial taxa and 118 microorganisms were detected. Community composition varied by location and water source type, with bank and mid-river sites generally exhibiting higher richness than puddles and a canal drain. Thirteen samples contained site-specific taxa, indicating marked spatial heterogeneity within and between communities. Microorganisms of public health relevance were frequently detected, including enteric-associated bacteria such as *Escherichia coli*, *Salmonella enterica*, *Shigella* species, and *Cronobacter sakazakii*, as well as opportunistic and healthcare-associated bacteria including *Acinetobacter baumannii*, *Klebsiella pneumoniae*, *Pseudomonas aeruginosa*, and *Stenotrophomonas maltophilia*. Higher-risk organisms were most widely distributed in Mepe and Battor, including in groundwater-associated sources. These findings provide molecular evidence of persistent, spatially heterogeneous microbial hazards months after flooding, underscoring continued exposure risks in downstream communities and the need for targeted water safety interventions and risk-informed public health responses.

Introduction

Events that occur post-flooding are a major concern for public health, stemming from the fact that there is the drastic deterioration of drinking and recreational water quality due to increased microbial contamination (1,2). Floodwaters act as vectors, transporting a diverse range of microbial pathogens and fecal contaminants from various sources, including agriculture, industrial effluents, human and animal waste, into receiving rivers and streams. This poses dire, long-lasting health challenges to affected communities (3,4). An increase in microbial contamination implies an increase in the risk of acquiring waterborne diseases such as dysentery, typhoid fever, leptospirosis, diarrhea etc. which may be life-threatening especially for the vulnerable (children, elderly individuals, immunocompromised individuals, pregnant women etc.) members of affected communities (5). An additional post-flood concern is that affected individuals are at risk of developing skin, ear, eye and respiratory infections due to the presence of diverse pathogens (6,7).

These infections, depending on their severity and repeated occurrences, may lead to chronic, life-long organ damage, examples being bladder cancer and liver fibrosis caused by Schistosomiasis infection (8,9). In addition to the risk of drowning from rising water levels, infections caused by pathogens found in waterbodies post-flood may lead to increased rates of mortality (10,11). Studies have consistently demonstrated a rise in fecal indicator bacteria in flood-affected rivers, emphasizing the increased likelihood of microbial contamination and subsequent health risks (12). The presence of indicator organisms such as *Escherichia coli*, fecal coliforms, enterococci, *Pseudomonas aeruginosa*, *Clostridium perfringens*, and specific viruses and protozoans, signals potential fecal contamination and the increased risk of pathogenic microorganisms known for causing diseases such as diarrhea, urinary tract infections (UTIs), cholera among many others (13,14). These contaminants pose significant health threats to communities that depend heavily on affected water sources for survival (domestic purposes) frequently leading to outbreaks of waterborne diseases (15–17).

Beyond the immediate public health concerns, the broader impacts of flooding extend to socio-economic consequences, including reduced agricultural yields and harsh effects on the health-

related quality of life for affected populations, manifesting as increased rates of depression, anxiety, and chronic pain that can persist for years (18,19).

The Kpong Dam, a significant hydroelectric facility on Ghana's Volta River, was constructed between 1977 and 1982 and since its construction, has been extremely beneficial to Ghana through the generation of electricity (20–22). In addition to power generation, the dam serves as a water source for irrigation and municipal water supply (23–25). However, its operation poses serious public health challenges for downstream communities, with Bilharzia (Schistosomiasis) endemic in some of these communities since its construction (26–28). Facing critical water levels due to unusually heavy rainfall in late 2023, the Volta River Authority (VRA) as part of regulatory duties initiated a controlled spillage with increased discharge volumes on September 15th 2023, to avert potential structural failure (29). This controlled spillage coinciding with heavy rainfalls at the time, resulted in widespread and devastating flooding across downstream communities along the Volta River highly impacting 7 districts of the Volta, Eastern and Greater Accra Regions. (30,31). Towns including Mepe, Sogakope, Battor, Adidome, Mafi, Tefle, Wume and Sokpoe were severely hit, with Mepe experiencing the worst inundation. The spillage triggered a humanitarian crisis, displacing tens of thousands of individuals, leading to significant losses of homes, livelihoods, and critical public health infrastructure (32,33).

Understanding the microbiological quality of water post-flood particularly in downstream communities is necessary for assessing the extent of contamination, identifying potential health threats, and implementing effective risk mitigation strategies and public health interventions. This paper aimed at assessing the potential emergence of pathogenic and potentially pathogenic bacteria post flooding, with implications for human and ecosystem health. We sought to comprehensively investigate the complex and varying effects of the Kpong Dam spillage on public health in the downstream communities of Asutsuare, Aveyime, Battor and Mepe communities using 16S rRNA gene sequencing of water samples from these towns.

Materials and Methods

The study utilized a cross-sectional approach to assess the presence and prevalence of pathogenic and potentially pathogenic bacteria in the downstream communities with implications for human health post-flooding. Water samples were collected from four townships; Asutsuare, Aveyime, Battor, and Mepe, located downstream of the Kpong dam in the North Tongu District of the Volta Region (Fig 1). These communities except for Asutsuare were affected by the controlled spillage from the dam. Sample collection was done in July 2024, when floodwaters had receded, and it was safe to go into the communities.

Fig 1: Map showing the Kpong dam and downstream communities where sampling was done.

Sampling design and sample collection

Sampling sites were selected in the field to represent various water sources within the community, including sections of the Volta River, taps, wells, boreholes, ponds, and other local water bodies. The exact locations of each sampling site were geo-referenced using a handheld GPS device, and coordinates were recorded to ensure spatial traceability. Water samples were collected using the direct immersion grab sampling method. Sterile bottles were immersed below the surface, and the caps were removed only after the bottles were submerged to minimize surface contamination and ensure representative water collection. At each site, 1.5 L of water was collected in sterile containers in accordance with Environmental Protection Agency (EPA) guidelines (34) for water sample collection. Each sample was given a unique identifier and transported on ice, in tightly sealed sterile styrofoam boxes. For quality control, samples were taken in duplicate from each site. A total of 22 samples were obtained from the Volta River, as well as other sources in the townships including wells, domestic taps, ponds, and puddles. Except for the Battor township where 7 samples were collected, 5 samples each were collected in the Asutsuare, Aveyime, and Mepe townships. In the Asutsuare township: 1

sample was obtained from a canal, 1 from the canal drain and 3 from different sections across the river. From Aveyime: 2 samples were obtained from the river, 1 from a pond, and 2 from two separate puddles, on the riverbank. From Battor: 4 samples were obtained from the river, and one each from a pond, borehole, and a domestic groundwater well. From Mepe: 1 sample was obtained from a pond; 3 obtained directly from the river (banks and middle), while one was collected from a household groundwater well.

Sample processing

Samples underwent vacuum filtration using a Whatman 0.45µm filter paper (Cytvia, UK). The filter papers were subsequently immersed in 1X phosphate-buffered saline solution to resuspend the cells. DNA was extracted using the QIAamp® DNA Mini Kit (Qiagen N.V., Germany), following the manufacturer's instructions. The extracted DNA was stored at -20^{0}C till use. The concentrations and purities of the extracts were assessed using a NanoDrop™ Spectrophotometer (Thermo Fisher Scientific, U.S.) at the Noguchi Memorial Institute for Medical Research, Accra, Ghana; and Qubit™ dsDNA HS quantification (Thermo Fisher Scientific, Waltham, Massachusetts, U.S.) at AttoDiagnostics Limited, Norwich, UK.

16S rRNA gene sequencing and post-sequencing analyses

16S rRNA gene sequencing was performed for 17 samples which were made up of 2 samples from Asutsuare, 5 from Aveyime, 6 from Battor, and 4 from Mepe. Five other samples were not processed due to low DNA quality. DNA sequencing was done at AttoDiagnostics Limited, Norwich, UK. 16S rRNA amplicons covering 8 out of the 9 hypervariable regions (HV2 to HV9) were amplified using the Ion AmpliSeq Microbiome Health Research Kit, followed by targeted sequencing using the Ion 540 Chip (Cat# 46496, ThermoFisher Scientific, USA) on the Ion GeneStudio™ S5 platform (Thermo Fisher Scientific, U.S.) following the manufacturers protocol. Demultiplexing and quality control processes were performed using Ion Reporter™ Software version 5.18 (Thermo Fisher Scientific, U.S.). Taxonomic assignments were made using the Greengenes database (version 13.5), while alpha diversity metrics were calculated and visualized using the RStudio IDE version 2025.05.0+496 ("Mariposa Orchid") (Posit Software, PBC). The minimum read counts for the different taxonomic levels were set at 500 for the genus and family, and 100 for the species level, while normalization thresholds were established at 0.5 for genus and family levels, and 0.25 for the

species. Results obtained after the sequencing and post-sequencing analyses were organized using Microsoft Excel LTSC Standard Version 16.99.1 (25072013).

Ecological and risk-based classification of microorganisms

Following 16S rRNA sequencing, microbial isolates were categorized using an ecological and risk-based classification framework into healthcare-associated, environmental/aquatic, enteric and opportunistic organisms. This enabled the grouping of microorganisms by both their likely origin and potential impact on human health. Healthcare-associated microbes included organisms commonly found in healthcare settings and typically associated with nosocomial infections; environmental and aquatic microbes referred to those naturally occurring in soil, water bodies and surrounding ecosystems without direct association with human disease. Enteric organisms were defined as those originating from faecal contamination and typically associated with the gastrointestinal tract of humans and animals while opportunistic pathogens included microbes which are generally harmless in healthy individuals but have the potential to cause infection in the immunocompromised. This classification facilitated the understanding of the microbial diversity present in the samples collected while highlighting potential health risks associated with different microbes.

Data analysis

All data was organized in Microsoft Excel Version 16.99.1 (25072013) and analyzed using RStudio (IDE version 2025.05.0+496 “Mariposa Orchid”; Posit Software, PBC) and the R packages *ggplot2*, *dplyr*, *tidyr*, *readr*, *viridis*, and *pheatmap*.

Results

Taxonomic composition and diversity of microbial isolates

From the 17 samples successfully sequenced, a total of 56 distinct microbial taxa and 118 distinct microorganisms were recorded (Table 1). Samples from all 4 towns were consistently dominated by Proteobacteria, whiles Bacteroidetes and Firmicutes also frequently co-occurred across sites. Acinetobacter were detected at lower relative abundances in several samples.

At the town level, samples from Asutsuare comprised 34 microbial taxa with the bank sample (ALBQ) showing the highest richness (28 taxa) and a strong dominance of Proteobacteria (89%). Multiple enteric and opportunistic organisms such as *Escherichia coli*, *Salmonella enterica*, *Shigella flexneri, Shigella sonnei, Acinetobacter baumannii, Klebsiella pneumoniae, Pseudomonas aeruginosa, and Stenotrophomonas maltophilia* were detected, whereas the canal drain sample (CDQ) exhibited lower diversity (6 taxa) and was largely composed of environmental and opportunistic organisms including *Bacteroides* spp., *Comamonas testosteroni*, *Sphingomonas* spp., and *Desulfovibrio* spp.

In Aveyime, a total of 31 taxa were identified across five sampling sites. Proteobacteria remained the dominant phylum in all the samples (64–89%), with variable representation of Bacteroidetes and Firmicutes. The bank and mid-river samples showed higher taxonomic richness compared with puddle samples, with several opportunistic Gram-negative species repeatedly detected. Detected organisms included environmental and opportunistic Gram-negative bacteria such as *Pseudomonas putida*, *Flavobacterium* spp., *Acidovorax* spp., and *Comamonas aquatica*, as well as potentially pathogenic taxa including *Escherichia coli*, *Aeromonas hydrophila*, *Aeromonas veronii*, and *Burkholderia cepacia*.

Samples from Battor showed the highest overall diversity, with 38 microbial taxa identified. Phylum-level distributions were more heterogeneous, with notable contributions from Firmicutes and Actinobacteria alongside Proteobacteria. Detected taxa included opportunistic Gram-negative organisms such as *Acinetobacter baumannii*, *Pseudomonas aeruginosa*, and *Stenotrophomonas maltophilia*, as well as Gram-positive and anaerobic taxa including *Bacillus* spp., *Clostridium* spp., *Staphylococcus* spp., and *Streptococcus* spp. Borehole and bank samples showed broad taxonomic representation, including anaerobic, environmental, and opportunistic organisms compared with the pond and mid-river samples.

In Mepe, 36 taxa were identified, with substantial variation across sites. The pond sample demonstrated complete dominance of Proteobacteria (100%) and included multiple enteric species such as *Escherichia coli*, *Salmonella enterica*, *Shigella boydii*, *Shigella flexneri*, and *Klebsiella* spp, while the bank and mid-river samples showed higher overall richness and broader phylum representation. Several samples displayed extensive representation of *Acinetobacter* species (*A. baumannii*, *A. lwoffii*, *A. calcoaceticus*) alongside other

opportunistic taxa such as *Pseudomonas* spp., *Legionella* spp., and *Stenotrophomonas maltophilia* (Table 1).

All 17 successfully sequenced samples displayed distinct microbial profiles. Of these, 13 samples contained microbial taxa that were not detected at any other sampling site indicating the presence of site-specific microorganisms. These unique taxa were distributed across all townships and multiple sample types, including bank, pond, mid-river, canal drain, borehole, and well samples. Several sampling sites were characterized by single unique taxa, such as *Desulfovibrio* spp. in the canal drain sample (CDQ), *Chitinimonas taiwanensis* in AV. A, *Alcaligenes faecalis* in BP, and *Bdellovibrio bacteriovorus* in the Mepe bank sample. Other sites recorded multiple unique microorganisms, including ALBQ, BB, BW, and M. Pond, each of which harbored between two and five taxa not identified elsewhere. The unique microorganisms identified spanned a range of ecological groups, including enteric bacteria (e.g., *Shigella sonnei*, *Shigella boydii*, *Enterobacter asburiae*), environmental and aquatic taxa (e.g., *Flavobacterium psychrophilum*, *Flavobacterium columnare*, *Bdellovibrio bacteriovorus*), and opportunistic or healthcare-associated organisms (e.g., *Acinetobacter nosocomialis*, *Acinetobacter junii*, *Chryseobacterium anthropi*). Together, these findings highlight pronounced site-level heterogeneity in microbial composition across the sampled environments (Table 1).

Table 1: Taxonomic Composition of Samples

Town	Sample ID	Total Microbial Taxa Identified	Phylum Level Distribution	Identified Microbial Species	Unique Microbes	Public Health Relevance
ASUTSUARE	ALBQ (Bank)	28	Proteobacteria 89%; Bacteroidetes 4%; Firmicutes 4%; Actinobacteria 3%	*Acinetobacter baumannii, Acidovorax sp., Aeromonas hydrophila, Bacillus cereus, Burkholderia sp., Citrobacter freundii, Cronobacter sakazakii, Enterobacter aerogenes, Enterobacter cloacae, Enterobacter hormaechei, Enterobacter ludwigii, Enterobacter sp., Escherichia coli, Flavobacterium sp., Iodobacter sp., Klebsiella oxytoca, Klebsiella pneumoniae, Legionella sp., Pantoea agglomerans, Propionibacterium acnes, Pseudomonas aeruginosa, Pseudomonas putida, Rickettsia endosymbiont, Salmonella enterica, Serratia marcescens, Shigella flexneri, Shigella sonnei, Stenotrophomonas maltophilia*	*Citrobacter freundii, Enterobacter hormaechei, Iodobacter sp., Shigella sonnei*	High microbial richness; strong fecal contamination signal (multiple enteric pathogens); several MDR-associated nosocomial organisms detected
	CDQ (Canal Drain)	6	Proteobacteria 67%; Bacteroidetes 33%	*Bacteroides sp., Comamonas sp., Comamonas testosteroni, Desulfovibrio sp., Sphingomonas sp., Sphingobacterium sp.*	*Desulfovibrio sp.*	Mostly low-virulence environmental/opportunistic taxa; no major high-risk pathogens
AVEYIME	AV. 1 (Puddle)	7	Proteobacteria 71%; Bacteroidetes 14%; Firmicutes 14%	*Pseudomonas aeruginosa, Escherichia coli, Aeromonas veronii, Stenotrophomonas sp., Bacillus sp., Burkholderia cepacia, Flavobacterium sp.*	None	Mixed fecal and opportunistic signal; moderate public-health concern
	AV. A (Puddle)	4	Proteobacteria 75%; Bacteroidetes 25%	*Acidovorax sp., Chitinimonas taiwanensis, Escherichia coli, Flavobacterium sp.*	*Chitinimonas taiwanensis*	Clear fecal contamination signal (*E. coli*) despite small sample size
	AV. B (Bank)	18	Proteobacteria 89%; Bacteroidetes 11%	*Acinetobacter baumannii, Acinetobacter sp., Aeromonas veronii, Alcaligenes sp., Brevundimonas sp., Burkholderia cepacia, Burkholderia sp., Chryseobacterium sp., Comamonas sp., Comamonas aquatica, Comamonas testosteroni, Flavobacterium sp., Pseudomonas aeruginosa, Pseudomonas putida, Pseudomonas sp., Rickettsia*	*Brevundimonas sp., Comamonas aquatica*	Cluster of MDR-associated HAIs mixed with aquatic opportunists; elevated clinical relevance

				endosymbiont, Sphingomonas sp., Stenotrophomonas maltophilia		
	AV. M (Mid-river)	9	Proteobacteria 78%; Bacteroidetes 22%	*Acidovorax facilis, Acidovorax sp., Alcaligenes sp., Enhydrobacter aerosaccus, Flavobacterium psychrophilum, Flavobacterium sp., Pseudomonas aeruginosa, Pseudomonas putida, Pseudomonas sp.*	*Acidovorax facilis, Enhydrobacter aerosaccus, Flavobacterium psychrophilum*	Environmental water community with notable *P. aeruginosa*, moderate human-health relevance
	AV. P (Pond)	14	Proteobacteria 64%; Bacteroidetes 29%; Firmicutes 7%	*Acidovorax sp., Aeromonas hydrophila, Aeromonas veronii, Alcaligenes sp., Bacillus sp., Chryseobacterium sp., Flavobacterium columnare, Flavobacterium sp., Flavisolibacter sp., Pseudomonas aeruginosa, Pseudomonas putida, Pseudomonas sp., Sphingomonas sp., Stenotrophomonas maltophilia*	*Flavobacterium columnare, Flavisolibacter sp.*	Freshwater / fish-associated taxa common; presence of opportunistic pathogens
BATTOR	BB (Borehole)	21	Proteobacteria 48%; Bacteroidetes 24%; Firmicutes 19%; Actinobacteria 10%	*Acinetobacter baumannii, Acinetobacter johnsonii, Acinetobacter sp., Aeromonas sp., Bacillus sp., Bacteroides sp., Burkholderia cepacia, Chryseobacterium sp., Clostridium sp., Escherichia coli, Flavobacterium sp., Klebsiella sp., Mycobacterium sp., Porphyromonas sp., Propionibacterium sp., Pseudomonas aeruginosa, Pseudomonas sp., Sphingobacterium sp., Staphylococcus sp., Stenotrophomonas sp., Streptococcus sp.*	*Clostridium sp., Porphyromonas sp., Propionibacterium sp., Staphylococcus sp., Streptococcus sp.*	Highest diversity in Battor; mix of oral/anaerobic, environmental and opportunistic species; broad opportunistic infection potential
	BP (Pond)	12	Proteobacteria 67%; Bacteroidetes 17%; Firmicutes 8%; Actinobacteria 8%	*Acinetobacter baumannii, Acinetobacter sp., Aeromonas sp., Alcaligenes faecalis, Bacillus sp., Burkholderia sp., Flavobacterium sp., Legionella sp., Mycobacterium sp., Pseudomonas sp., Sphingobacterium sp., Stenotrophomonas maltophilia*	*Alcaligenes faecalis*	Combined HAI-Aquatic signature; notable respiratory-associated pathogens

	BVRHq (*Volta River along Battor Holy Quarters*)	11	Proteobacteria 45%; Bacteroidetes 36%; Firmicutes 9%; Actinobacteria 9%	*Acinetobacter baumannii, Acinetobacter sp., Aeromonas veronii, Bacillus sp., Chryseobacterium sp., Flavobacterium sp., Flavobacterium chungnamense, Flavobacterium macrobrachii, Propionibacterium acnes, Pseudomonas putida, Stenotrophomonas maltophilia*	*Flavobacterium chungnamense, Flavobacterium macrobrachii*	Aquatic/soil organisms common; moderate clinical relevance with some opportunists
	BVRm (Mid-river)	12	Proteobacteria 92%; Bacteroidetes 8%	*Acinetobacter junii, Acinetobacter nosocomialis, Acinetobacter baumannii, Acinetobacter calcoaceticus, Acinetobacter sp., Aeromonas hydrophila, Aeromonas veronii, Aeromonas sp., Escherichia coli, Flavobacterium sp., Pseudomonas sp., Stenotrophomonas sp.*	*Acinetobacter junii, Acinetobacter nosocomialis*	Moderate microbial load; mix of opportunistic and enteric organisms; some MDR indicators
	BVRo (Bank)	20	Proteobacteria 70%; Bacteroidetes 15%; Firmicutes 10%; Actinobacteria 5%	*Acinetobacter johnsonii, Acinetobacter baumannii, Acinetobacter calcoaceticus, Acinetobacter sp., Aeromonas hydrophila, Aeromonas veronii, Bacillus cereus, Bacillus sp., Bacteroides sp., Burkholderia sp., Burkholderia cepacia, Chryseobacterium sp., Escherichia coli, Flavobacterium sp., Klebsiella pneumoniae, Legionella sp., Propionibacterium acnes, Pseudomonas putida, Pseudomonas sp., Stenotrophomonas maltophilia*	None	High complexity: strong healthcare-associated signature plus aquatic *Legionella;* elevated risk
	BW (well)	15	Proteobacteria 67%; Bacteroidetes 27%; Firmicutes 7%	*Aeromonas hydrophila, Aeromonas veronii, Acinetobacter baumannii, Bacillus sp., Chryseobacterium anthropi, Chryseobacterium hominis, Chryseobacterium sp., Escherichia coli, Flavobacterium sp., Pseudomonas alcaligenes, Pseudomonas aeruginosa, Pseudomonas putida, Pseudomonas sp., Pseudomonas stutzeri, Stenotrophomonas maltophilia*	*Chryseobacterium anthropi, Chryseobacterium hominis, Pseudomonas alcaligenes, Pseudomonas stutzeri*	High load of opportunistic Gram-negative rods; several *Chryseobacterium* spp. present
MEPE	M. Bank (Bank)	23	Proteobacteria 74%; Bacteroidetes 13%; Actinobacteria 9%; Firmicutes 4%	*Aeromonas hydrophila, Acinetobacter johnsonii, Acinetobacter tjernbergiae, Acinetobacter radioresistens, Acinetobacter baumannii, Acinetobacter calcoaceticus, Acinetobacter sp., Acinetobacter lwoffii, Acinetobacter beijerinckii, Bacillus sp., Bdellovibrio bacteriovorus, Burkholderia cepacia, Burkholderia sp., Chryseobacterium sp., Flavobacterium sp., Legionella sp., Pseudomonas putida,*	*Bdellovibrio bacteriovorus*	Highest richness overall; diverse Acinetobacter population and mix of environmental and HAI-associated taxa

				Pseudomonas sp., Rhodococcus erythropolis, Rhodococcus sp., Sphingobacterium sp., Stenotrophomonas maltophilia, Thermomonas haemolytica		
	M. Pond (Pond)	22	Proteobacteria 100%	*Acinetobacter baumannii, Aeromonas hydrophila, Aeromonas veronii, Cronobacter sakazakii, Enterobacter aerogenes, Enterobacter asburiae, Enterobacter cloacae, Enterobacter ludwigii, Enterobacter sp., Escherichia coli, Klebsiella oxytoca, Klebsiella sp., Pantoea agglomerans, Pectobacterium carotovorum, Pseudomonas aeruginosa, Salmonella enterica, Serratia marcescens, Serratia sp., Shigella boydii, Shigella flexneri, Stenotrophomonas maltophilia, Xanthomonas sp.*	*Enterobacter asburiae, Pectobacterium carotovorum, Serratia sp., Shigella boydii, Xanthomonas sp.*	Severe fecal contamination signal; many clinically significant enteric pathogens, high public-health concern
	T. Bank (Bank)	4	Proteobacteria 75%; Bacteroidetes 25%	*Aeromonas hydrophila, Acinetobacter sp., Flavobacterium sp., Stenotrophomonas maltophilia*	None	Low-diversity sample dominated by opportunistic species
	T. Middle (Mid-river)	21	Proteobacteria 71%; Bacteroidetes 14%; Actinobacteria 10%; Firmicutes 5%	*Acinetobacter johnsonii, Acinetobacter tjernbergiae, Acinetobacter radioresistens, Acinetobacter baumannii, Acinetobacter calcoaceticus, Acinetobacter sp., Acinetobacter lwoffii, Acinetobacter beijerinckii, Bacillus sp., Burkholderia sp., Burkholderia cepacia, Chryseobacterium sp., Flavobacterium sp., Legionella sp., Pseudomonas putida, Pseudomonas sp., Rhodococcus sp., Rhodococcus erythropolis, Sphingobacterium sp., Stenotrophomonas maltophilia, Thermomonas haemolytica*	None	Extensive *Acinetobacter* representation; strong HAI signature with mixed opportunistic load

Across all the towns, bank and mid-river samples generally exhibited higher microbial richness compared with puddle and canal drain samples. Unique taxa were detected at multiple sites, indicating localized microbial signatures within and between communities. There were nine unique microbes identified in the Aveyime Township, none of which were detected in the other townships. Similarly, 5, 16, and 13 unique microbes were identified in Asutsuare, Battor and Mepe respectively. The UpSet plot (Fig 2) further illustrates the distribution and overlap of microbial taxa across the four townships, highlighting both shared microbial communities and township-specific taxa.

Fig 2: UpSet plot showing the distribution and overlap of microbial taxa across townships. The left horizontal bar chart (A) represents the total number of unique microbial taxa identified within each individual township. The main vertical bar chart (B), together with the intersection matrix (C), shows the number of microbial taxa present exclusively in specific township combinations, as indicated by dark dots, and absent from the remaining townships.

Microbial community structure and beta diversity

Principal Coordinates Analysis (PCoA) was used to assess beta diversity and examine differences in microbial community composition across sampling sites and townships (Fig 3). The first two principal coordinate axes explained 21.57% (PCoA Axis 1) and 17.57% (PCoA Axis 2) of the total variation, capturing the major gradients in community dissimilarity among samples. Distinct clustering and dispersion patterns were observed across towns with samples from Battor clustering within the lower right quadrant of the ordination space, with relatively uniform spacing, indicating a moderately consistent microbial community structure across sites. In contrast, Aveyime samples exhibited greater dispersion, reflecting higher within-site variability in community composition. Mepe samples were heterogeneously distributed, with bank and mid-river samples clustering together yet clearly separated from other sites within the town, suggesting spatial heterogeneity in microbial communities within the township. Samples from Asutsuare displayed the greatest degree of dispersion in ordination space, indicating pronounced variation in microbial community composition among sites. Notably,

the Asutsuare bank sample (ALBQ) clustered closely with samples AV.1 (Aveyime) and M. Pond (Mepe), suggesting partial overlap in microbial community structure across townships. This overlap may reflect shared environmental characteristics or hydrological connectivity influencing microbial assemblages at these sites.

Fig 3: Principal Coordinates Analysis (PCoA) plot representing spatial variations in microbial community compositions across sampling sites and towns. Each point represents a unique sampling site with points coloured by townships (Mepe , Battor , Aveyime , Asutsuare . The distance between points reflects the degree of dissimilarity in the microbial composition. The plot uses two main axes, PCoA Axis 1 (explaining 21.57% of the variation) and PCoA Axis 2 (explaining 17.57% of the variation), to capture the largest dimensions of microbial community differences.

Risk-based stratification of microorganisms with ecological subclassification

The identified microorganisms were classified both by risk levels and ecological origin. Microorganisms were stratified broadly by risk levels into high, medium, and low categories. Within each risk level, organisms were further categorized according to their ecological classification, such that healthcare-associated pathogens, environmental/aquatic organisms, enteric pathogens, and opportunistic organisms were represented as subsets of the respective risk categories (Table 2) .

Table 2: Distribution of microbial taxa across risk levels and ecological categories

Town	Risk Level	Ecological Category	Frequency	Specific Microbes	Associated Diseases Summary
ASUTSUARE	High	Healthcare-associated	6	*Acinetobacter baumannii; Klebsiella oxytoca; Klebsiella pneumoniae; Pseudomonas aeruginosa; Stenotrophomonas maltophilia; Serratia marcescens*	Major nosocomial infections including pneumonia, bloodstream infections, surgical-site infections, Urinary Tract Infections; Multi-Drug Resistance concerns.
		Environmental/Aquatic	1	*Legionella sp.*	Legionnaires' disease, Pontiac fever.
		Enteric	5	*Escherichia coli; Salmonella enterica; Shigella flexneri; Shigella sonnei; Cronobacter sakazakii*	Diarrhea, gastroenteritis, neonatal infection risk (*C. sakazakii*).
		Opportunistic	0		
	Medium	Healthcare-associated	6	*Enterobacter aerogenes; Enterobacter cloacae; Enterobacter hormaechei; Enterobacter ludwigii; Enterobacter sp.; Citrobacter freundii*	Healthcare-associated infections including bacteremia, Urinary Tract Infections, wound infections.
		Environmental/Aquatic	0		
		Enteric	3	*Aeromonas hydrophila; Bacillus cereus; Aeromonas sp.*	Gastroenteritis, wound infections (*Aeromonas*); food poisoning (*B. cereus*).
		Opportunistic	7	*Pseudomonas putida; Pantoea agglomerans; Burkholderia sp.; Bacteroides sp.; Comamonas sp.; Comamonas testosteroni Sphingomonas sp.*	Opportunistic infections (wound, device-related, respiratory).
	Low	Healthcare-associated	1	*Propionibacterium acnes*	Low-virulence skin commensal; device-related infections.
		Environmental/Aquatic	4	*Flavobacterium sp.; Iodobacter sp.; Acidovorax sp. Desulfovibrio sp.*	Low direct human risk; fish/plant-associated bacteria.
		Enteric	0		
		Opportunistic	1	*Rickettsia endosymbiont*	Endosymbiont; very low pathogenicity.
AVEYIME	High	Healthcare-associated	4	*Acinetobacter baumannii; Pseudomonas aeruginosa; Stenotrophomonas maltophilia; Burkholderia cepacia*	Nosocomial infections with Multi-Drug Resistance trends; pneumonia, bloodstream infections.

		Environmental/Aquatic	0		
		Enteric	1	*Escherichia coli*	Diarrheal disease, Urinary Tract Infections.
		Opportunistic	1	*Stenotrophomonas maltophilia*	Opportunistic Multi-Drug Resistance pathogen.
	Medium	Healthcare-associated	3	*Acinetobacter sp.; Alcaligenes sp.; Stenotrophomonas sp.*	Device-associated, wound, respiratory opportunistic infections.
		Environmental/Aquatic	3	*Flavobacterium sp.; Flavobacterium columnare; Flavobacterium psychrophilum*	Environmental/fish pathogens; occasional rare human infections.
		Enteric	4	*Aeromonas hydrophila; Aeromonas veronii; Chitinimonas taiwanensis; Bacillus sp.*	Gastroenteritis, wound infections.
		Opportunistic	9	*Comamonas sp.; Comamonas aquatica; Comamonas testosteroni; Pseudomonas putida; Pseudomonas sp.; Chryseobacterium sp.; Sphingomonas sp.; Bacillus sp.; Alcaligenes sp.*	Opportunistic infections, Respiratory, wound, bloodstream.
	Low	Healthcare-associated	1	*Brevundimonas sp.*	Rare opportunistic infections; generally low risk.
		Environmental/Aquatic	2	*Acidovorax sp.; Enhydrobacter aerosaccus*	Environmental bacteria; low human disease risk.
		Enteric	0		
		Opportunistic	1	*Rickettsia endosymbiont*	Very low pathogenicity.
BATTOR	High	Healthcare-associated	7	*Acinetobacter baumannii; Klebsiella pneumoniae; Pseudomonas aeruginosa; Stenotrophomonas maltophilia; Klebsiella sp.; Serratia sp.; Mycobacterium sp.;*	Major nosocomial pathogens; Multi-Drug Resistance; pneumonia, Urinary Tract Infections, bloodstream infections.
		Environmental/Aquatic	1	*Legionella sp.*	Legionnaires' disease.
		Enteric	3	*Escherichia coli; Salmonella enterica; Shigella spp.*	Enteric infections; diarrhea; neonatal infection risk.

		Opportunistic	2	*Burkholderia cepacia; Stenotrophomonas sp.*	Multi-Drug Resistance respiratory opportunistic pathogens.
	Medium	Healthcare-associated	8	*Acinetobacter calcoaceticus; Acinetobacter johnsonii; Acinetobacter junii; Acinetobacter sp.; Citrobacter freundii; Propionibacterium acnes; Staphylococcus sp.; Streptococcus sp.*	Opportunistic Healthcare-associated Infections: wound, device-related, skin/soft tissue infections.
		Environmental/Aquatic	6	*Pseudomonas sp.; Rhodococcus sp.; Flavobacterium sp.; Thermomonas haemolytica; Porphyromonas sp.; Chryseobacterium spp.*	Opportunistic and environmental microbes; occasional infections.
		Enteric	4	*Aeromonas hydrophila; Aeromonas veronii; Aeromonas sp.; Bacillus cereus*	Gastroenteritis, wound infections.
		Opportunistic	12	*Burkholderia sp.; Chryseobacterium anthropi; Chryseobacterium hominis; Pseudomonas putida; Pseudomonas alcaligenes; Pseudomonas stutzeri; Bacillus sp.; Alcaligenes faecalis; Mycobacterium sp.; Bacteroides sp.; Propionibacterium sp.; Porphyromonas sp.*	Opportunistic infections including respiratory, wound, bloodstream.
	Low	Healthcare-associated	3	*Acinetobacter beijerinckii; Acinetobacter lwoffii; Acinetobacter radioresistens*	Low-virulence opportunists.
		Environmental/Aquatic	4	*Flavobacterium sp.; Flavobacterium chungnamense; Flavobacterium macrobrachii; Bdellovibrio bacteriovorus*	Environmental species; low human disease risk.
		Enteric	0		
		Opportunistic	1	*Sphingobacterium sp.*	Rare opportunistic infections.
MEPE	High	Healthcare-associated	6	*Acinetobacter baumannii; Pseudomonas aeruginosa; Burkholderia cepacia; Klebsiella oxytoca; Serratia marcescens; Pseudomonas sp.*	Nosocomial high-risk pathogens; Multi-Drug Resistance; pneumonia, sepsis, Urinary Tract Infections.
		Environmental/Aquatic	1	*Legionella sp.*	Legionnaires' disease.
		Enteric	5	*Escherichia coli; Salmonella enterica; Shigella boydii; Shigella flexneri; Cronobacter sakazakii*	Severe gastroenteritis; neonatal *Cronobacter* infections.

		Opportunistic	2	*Stenotrophomonas maltophilia; Burkholderia cepacia*	Multi-Drug Resistance respiratory opportunists.
	Medium	Healthcare-associated	10	*Enterobacter aerogenes; Enterobacter cloacae; Klebsiella sp.; Acinetobacter johnsonii; Acinetobacter calcoaceticus; Acinetobacter beijerinckii; Acinetobacter lwoffii; Acinetobacter radioresistens; Acinetobacter tjernbergiae; Acinetobacter sp.*	Healthcare-associated opportunists.
		Environmental/Aquatic	6	*Flavobacterium sp.; Rhodococcus spp.; Iodobacter sp.; Flavisolibacter sp.; Flavobacterium chungnamense; Flavobacterium macrobrachii*	Environmental/fish/soil organisms.
		Enteric	2	*Aeromonas hydrophila; Aeromonas veronii*	Gastroenteritis, wound infections.
		Opportunistic	13	*Pseudomonas putida; Pseudomonas sp.; Rhodococcus erythropolis; Rhodococcus sp.; Chryseobacterium sp.; Serratia sp.; Enterobacter asburiae; Enterobacter ludwigii; Pantoea agglomerans; Bacillus sp.; Acinetobacter spp.; Xanthomonas sp.; Sphingobacterium sp.*	Opportunistic infections; moderate risk.
	Low	Healthcare-associated	4	*Acinetobacter sp.; Pseudomonas putida; Propionibacterium acnes; Mycobacterium sp.*	Low-virulence Healthcare-associated Infections or environmental species.
		Environmental/Aquatic	3	*Pectobacterium carotovorum; Bdellovibrio bacteriovorus; Thermomonas haemolytica*	Plant/soil-associated species; low human risk.
		Enteric	0		
		Opportunistic	0		

The spatial frequency and site-level distribution of pathogenic and potentially pathogenic taxa are illustrated in Fig 4 providing spatial context to the observed risk profiles. The figure demonstrates that clinically relevant microorganisms were not uniformly distributed across sites but instead showed clear site-specific and hydrology-linked patterns. Bank, mid-river, and pond environments exhibited higher frequencies of detection of pathogenic species compared with canal drain and puddle sites, Multiple pathogenic taxa were detected at more than one site within the same town, while other taxa were restricted to single sampling locations.

Variation in detection frequency was observed across towns, with differences in the number of sites at which pathogenic taxa were detected. Sites exhibiting higher clustering of pathogenic taxa corresponded to towns with greater representation of high- and medium-risk organisms in the risk-based classification (Table 2).

Fig 4: Bubble plot providing an overview of the different pathogenic microbes found at specific sampling sites, as well as the overall microbial prevalence. The figure illustrates the detection patterns of clinically relevant microorganisms classified as high- or medium-risk across sampling sites. Each point represents the presence of a pathogenic or potentially pathogenic taxon at a given site. The distribution highlights site-specific and repeated detection patterns of organisms of public health importance across towns and hydrological settings.

Town-level distribution of risk-classified microorganisms

Asutsuare

Microbes were found across all risk levels and spanned most of the ecological categories. High-risk microbes found included healthcare-associated pathogens such as *Acinetobacter baumanii*, *Klebsiella oxytoca*, *Klebsiella pneumoniae*, and *Serratia marcescens*, enteric pathogens such as *Escherichia coli*, *Salmonella enterica*, *Shigella flexneri*, and *Cronobacter sakazakii*; and *Legionella sp.* as the only environmental/aquatic organism. No high-risk opportunistic microbes were detected. Medium-risk organisms included healthcare-

associated pathogens such as *Citrobacter freundii*, and multiple *Enterobacter* species; and enteric pathogens including *Aeromonas hydrophila*, and *Bacillus cereus*. Opportunistic pathogens included *Pseudomonas putida*, *Pantoea agglomerans*, *Burkholderia sp.*, *Shingomonas sp.* and *Comamonas testosteroni and* no medium-risk environmental/aquatic microbes were found. Low-risk organisms included *Propionibacterium acnes* as the single healthcare-associated pathogen; *Flavobacterium sp.*, *Iodobacter sp.*, *Acidovorax sp.*, and *Desulfovibrio sp.* as environmental/aquatic microbes; and *Rickettsia endosymbiont* as the single opportunistic pathogen. No low risk enteric microbes were detected.

Aveyime

Samples from the town harboured a high burden of medium-risk opportunistic taxa. The high-risk microbes detected included healthcare-associated pathogens: *Acinetobacter baumannii*, *Pseudomonas aeruginosa*, *Stenotrophomonas maltophilia*, and *Burkholderia cepacia*. *Escherichia coli* and *S. maltophilia* were the sole enteric and opportunistic pathogens identified respectively. No high-risk environmental/aquatic microbes were detected. Medium-risk organisms detected include the healthcare-associated pathogens: *Acinetobacter sp.*, *Alcaligenes sp.*, and *Stenotrophomonas sp.* while multiple *Flavobacterium* species were identified as environmental/aquatic microbes. The Enteric pathogens detected were *Aeromonas hydrophila*, *Aeromonas veronii*, *Bacillus sp.*, and *Chitinimonas taiwanensis* and opportunistic pathogens included *Alcaligenes sp.*, *Bacillus sp.*, and multiple *Comamonas and Pseudomonas* species. In the low risk category, *Brevundimonas sp.* and *Rickettsia endosymbiont* were the sole low-risk healthcare-associated and opportunistic pathogens identified respectively. *Acidovorax sp.*, and *Enhydrobacter aerosaccus* were detected as low-risk environmental/aquatic microbes and no low-risk enteric pathogens were identified.

Battor

Battor showed the widest distribution of microorganisms across all risk levels and ecological categories. High-risk healthcare-associated pathogens such as *Acinetobacter baumanii*, *Burkholderia cepacia*, *Klebsiella oxytoca*, and *Serratia marcescens* were identified whiles *Legionella sp.* was the only high-risk environmental/aquatic organism detected. The Enteric pathogens detected included *Escherichia coli*, *Shigella spp.* and *Salmonella enterica*, while

opportunistic pathogens identified were *Burkholderia cepacia* and *Stenotrophomonas spp.* Medium-risk organisms included healthcare-associated pathogens, such as multiple *Acinetobacter* species, *Citrobacter freundii*, and *Propionibacterium acnes*, as well as environmental/aquatic taxa including *Pseudomonas* spp., *Rhodococcus* spp., and *Thermomonas haemolytica*. This risk category also comprised potentially pathogenic enteric organisms, such as multiple *Aeromonas* species and *Bacillus cereus*, together with opportunistic pathogens including *Burkholderia* spp., *Chryseobacterium anthropi*, *Chryseobacterium hominis*, *Alcaligenes faecalis*, and multiple *Pseudomonas* species. Low-risk healthcare-associated pathogens detected included *Acinetobacter beijerinckii*, *Acinetobacter lwoffii*, and *Acinetobacter radioresistens*. Low-risk environmental/aquatic organisms comprised multiple *Flavobacterium* species and *Bdellovibrio bacteriovorus*, while *Sphingobacterium* spp. was the only low-risk opportunistic pathogen identified. No enteric pathogens were detected within this risk category.

Mepe

Microorganisms detected in Mepe spanned all three risk levels and several ecological categories. High-risk organisms included healthcare-associated pathogens such as *Acinetobacter baumannii*, *Pseudomonas aeruginosa*, and *Burkholderia cepacia*. *Legionella* spp. was detected as the sole high-risk environmental/aquatic organism. High-risk enteric pathogens comprised *Escherichia coli*, *Salmonella enterica*, and multiple *Shigella* species, while *Stenotrophomonas maltophilia* and *Burkholderia cepacia* were the only high-risk opportunistic pathogens identified. Medium-risk organisms included healthcare-associated pathogens such as multiple *Acinetobacter* and *Enterobacter* species, as well as *Klebsiella* spp. Environmental/aquatic organisms detected at this risk level included *Flavisolibacter* spp. and multiple *Flavobacterium* species. The enteric pathogens identified were *Aeromonas hydrophila* and *Aeromonas veronii*, while opportunistic pathogens comprised multiple *Pseudomonas*, *Rhodococcus*, and *Enterobacter* species. Low-risk organisms included healthcare-associated taxa such as *Acinetobacter* spp. and *Pseudomonas putida*. Environmental/aquatic organisms detected at this risk level included *Pectobacterium*

carotovorum, *Bdellovibrio bacteriovorus*, and *Thermomonas haemolytica*. No low-risk enteric or opportunistic pathogens were detected.

Discussion

This study provides molecular evidence of microbial contamination and potential public health risk in downstream communities along the Volta River following the 2023 controlled spillage of the Akosombo and Kpong dams. Using 16S rRNA gene sequencing, we identified diverse microbial communities across multiple water sources in Asutsuare, Aveyime, Battor, and Mepe, including enteric pathogens, opportunistic organisms, and healthcare-associated bacteria of established public health relevance. Recent evidence from a longitudinal study has shown that flood-impacted aquatic environments can remain microbiologically compromised for extended periods after floodwaters recede, particularly in low and middle-income settings where sanitation and water infrastructure are disrupted (35). The findings from this study are consistent with these observations and highlight the persistence of microbial hazards in downstream environments months after flooding events.

Across all the towns, microbial communities were dominated by Proteobacteria, with frequent co-occurrence of Bacteroidetes and Firmicutes. Similar taxonomic patterns have been reported in flood-affected rivers and disturbed freshwater systems, where increased nutrient loading and hydrological disturbance favour Proteobacteria-rich assemblages (36,37). In this study, bank and mid-river samples generally exhibited higher microbial richness than puddle and canal drain samples. Comparable richness patterns have been documented in recent flood and riverine microbiome studies, where hydrologically connected environments support more diverse microbial communities than isolated or stagnant waters (38,39). For downstream communities that rely on riverbanks and mid-channel waters for domestic activities, this increased diversity includes a broader spectrum of organisms with potential public health implications (40).

Beta diversity analysis further demonstrated pronounced spatial heterogeneity in microbial community composition across sampling sites and towns. Partial clustering by town was observed alongside substantial within-town dispersion, particularly in Asutsuare and Mepe.

Similar patterns of spatial heterogeneity have been described in aquatic microbial ecology studies, where local environmental conditions, water source type, and contamination inputs shape microbial communities over short spatial scales (41). The overlap observed between selected sites across different towns likely reflects shared hydrological connectivity within the Volta River system, a feature that has been noted in large river basins affected by dam operations and controlled spillages (42).

A key public health signal in this study is the repeated detection of enteric-associated organisms, including *Escherichia coli*, *Salmonella enterica*, *Shigella* spp., and *Cronobacter sakazakii*, across multiple towns and water source types. Floodwaters are known to mobilize faecal contaminants from sanitation facilities, agricultural areas, and animal waste into surface and groundwater sources, thereby increasing the risk of waterborne disease transmission (35,43). Recent studies in flood-affected settings have consistently linked elevated levels of enteric bacteria with increased incidence of diarrhoeal and gastrointestinal diseases, particularly among children and other vulnerable populations (44). The strong enteric signal observed in Mepe's pond sample is therefore of particular concern given the documented severity of flooding and displacement in this community.

In addition to enteric pathogens, this study identified multiple opportunistic and healthcare-associated organisms of clinical relevance, including *Acinetobacter baumannii*, *Klebsiella pneumoniae*, *Pseudomonas aeruginosa*, and *Stenotrophomonas maltophilia*. Recent environmental and public health research increasingly recognizes natural and engineered water systems as reservoirs for such organisms, especially in post-flood contexts where infrastructure damage and altered water flow facilitate environmental dissemination (35,45). The detection of these taxa in groundwater-associated sources such as wells and boreholes, particularly in Battor, aligns with reports that shallow or poorly protected groundwater sources are vulnerable to contamination following flooding events (46).

The integration of ecological classification with risk-based stratification provides a structured framework for interpreting microbial findings in a public health context. Similar risk-oriented approaches have been recommended in recent environmental health literature which advocates adapting Quantitative Microbial Risk Assessment (QMRA) frameworks for rural and remote community water systems by incorporating source-specific ecological heterogeneity to enable tiered risk prioritization beyond presence-absence reporting (47). The

pathogen-focused spatial analysis in this study demonstrates that pathogenic and potentially pathogenic taxa are not uniformly distributed across sampling sites, with repeated detection at certain locations and restriction to single sites at others. Such spatial heterogeneity has been widely reported in flood-affected water systems and underscores the importance of site-specific surveillance and intervention rather than uniform community-wide risk assumptions (35,48–51).

Our study also presents with a few limitations Detection of bacterial DNA using 16S rRNA sequencing does not confirm organism viability or infectivity, and species-level resolution may be limited for some taxa. The cross-sectional design, conducted after floodwaters had receded, does not capture temporal dynamics during the acute flooding phase. In addition, antimicrobial resistance profiles were not assessed, despite the detection of organisms commonly associated with multidrug resistance. Nonetheless, the combined taxonomic, beta diversity, and risk-based analyses provide a comprehensive snapshot of microbial hazards in downstream communities following dam-related flooding. Future studies should prioritize longitudinal sampling across seasons, quantitative microbial assays, culture-based confirmation, and resistance gene profiling to better characterize ongoing public health risks in downstream flood-affected settings.

Overall, our study highlights that downstream flood-affected communities along the Volta River system are exposed to spatially heterogeneous microbial risks that vary by water source type and location. The persistence of enteric, opportunistic, and healthcare-associated bacteria months after flooding underscores the need for targeted water safety interventions, strengthened environmental surveillance, and context-specific public health responses in communities impacted by dam spillages and extreme hydrological events.

Acknowledgments

We acknowledge the School of Basic and Biomedical Sciences, University of Health and Allied Sciences for allowing us to use their labs for this work. We would also like to acknowledge Prof Anthony Ablordey and Dr Prince Asare of the Noguchi Memorial Institute for Medical Research for their assistance.

References

1. Percy GV, Thiyaneshwaran KK. Effect Of Flood Event On Water Quality. Rasayan J Chem [Internet]. 2019 Jun [cited 2025 Jul 22];12(2):849–54. Available from: http://www.rasayanjournal.comhttp://www.rasayanjournal.co.in
2. Talbot CJ, Bennett EM, Cassell K, Hanes DM, Minor EC, Paerl H, et al. The impact of flooding on aquatic ecosystem services. Biogeochemistry [Internet]. 2018 Dec 1 [cited 2024 Sep 10];141(3):439–61. Available from: https://link.springer.com/article/10.1007/s10533-018-0449-7
3. Collender PA, Cooke OC, Bryant LD, Kjeldsen TR, Remais J V. Estimating the microbiological risks associated with inland flood events: Bridging theory and models of pathogen transport. Crit Rev Environ Sci Technol [Internet]. 2016 Dec 16 [cited 2025 Jul 22];46(23–24):1787. Available from: https://pmc.ncbi.nlm.nih.gov/articles/PMC5533301/
4. Shucksmith J. Modelling the Transport of Contaminants in Urban Flood Flows. 2017.
5. Haldar K, Kujawa-Roeleveld K, Hofstra N, Datta DK, Rijnaarts H. Microbial contamination in surface water and potential health risks for peri-urban farmers of the Bengal delta. Int J Hyg Environ Health [Internet]. 2022 Jul 1 [cited 2025 Jul 22];244:114002. Available from: https://www.sciencedirect.com/science/article/pii/S1438463922000852
6. Minnesota Department of Health. Causes and Symptoms of Waterborne Illness - MN Dept. of Health [Internet]. 2025 [cited 2025 Jul 22]. Available from: https://www.health.state.mn.us/diseases/waterborne/basics.html
7. Huang LY, Wang YC, Wu CC, Chen YC, Huang YL. Risk of Flood-Related Diseases of Eyes, Skin and Gastrointestinal Tract in Taiwan: A Retrospective Cohort Study. PLoS One [Internet]. 2016 May 1 [cited 2026 Jan 12];11(5):e0155166. Available from: https://pmc.ncbi.nlm.nih.gov/articles/PMC4865035/
8. NHS. Schistosomiasis (bilharzia) - NHS [Internet]. 2022 [cited 2025 Jul 22]. Available from: https://www.nhs.uk/conditions/schistosomiasis/

9. Marie C, Petri WAJ. Schistosomiasis - Infections [Internet]. 2024 [cited 2025 Jul 22]. Available from: https://www.merckmanuals.com/home/infections/parasitic-infections-trematodes-flukes/schistosomiasis

10. Lynch VD, Sullivan JA, Flores AB, Xie X, Aggarwal S, Nethery RC, et al. Large floods drive changes in cause-specific mortality in the United States. Nat Med [Internet]. 2025 Feb 1 [cited 2025 Jul 22];31(2):663. Available from: https://pmc.ncbi.nlm.nih.gov/articles/PMC11835711/

11. Lawal K. Flooding and Waterborne Diseases Affecting Pregnant Women in Lagos Slums. 2025 May 6 [cited 2025 Jul 22]; Available from: https://www.researchgate.net/publication/391459139_Flooding_and_Waterborne_Diseases_Affecting_Pregnant_Women_in_Lagos_Slums

12. Mukherjee M, Hossain MS, Boswell J, Zhang Y, Allen MS, LaMontagne MG, et al. Large, but short-term, increase in fecal indicator bacteria following extreme flooding from Hurricane Harvey in Houston, TX. Frontiers in Water [Internet]. 2024 Nov 12 [cited 2025 Jul 22];6:1466377. Available from: https://qiime2.org;

13. Myers DT, Zgleszewski L, Bier R, Price JR, Morgan S, Jackson JK, et al. High fecal indicator bacteria in temperate headwater streams at baseflow: implications for management and public health. Water Res X [Internet]. 2025 Dec 1 [cited 2025 Jul 22];29:100347. Available from: https://www.sciencedirect.com/science/article/pii/S2589914725000465

14. Rochelle-Newall E, Nguyen TMH, Le TPQ, Sengtaheuanghoung O, Ribolzi O. A short review of fecal indicator bacteria in tropical aquatic ecosystems: Knowledge gaps and future directions. Front Microbiol [Internet]. 2015 Apr 17 [cited 2025 Jul 22];6(MAR):135738. Available from: www.frontiersin.org

15. Acosta-España JD, Romero-Alvarez D, Luna C, Rodriguez-Morales AJ. Infectious disease outbreaks in the wake of natural flood disasters: global patterns and local implications. Infez Med [Internet]. 2024 [cited 2025 Jul 16];32(4):451. Available from: https://pmc.ncbi.nlm.nih.gov/articles/PMC11627491/

16. Kimberlin DW, Jester PM, Sánchez PJ, Ahmed A, Arav-Boger R, Michaels MG, et al. Valganciclovir for Symptomatic Congenital Cytomegalovirus Disease. New England Journal of Medicine. 2015 Mar 5;372(10):933–43.

17. Kofi Yeboah SII, Antwi-Agyei P, Kabo-Bah AT, Bonsu Ackerson NO. Water, environment, and health nexus: understanding the risk factors for waterborne diseases in communities along the Tano River Basin, Ghana. J Water Health [Internet]. 2024 Aug 1 [cited 2025 Jul 22];22(8):1556–77. Available from: http://iwaponline.com/jwh/article-pdf/22/8/1556/1468850/jwh2024186.pdf

18. Abass K, Gyasi RM, Katey D, Frempong F, Garsonu EK. Flood exposure and psychological distress among Ghanaian adults in flood-prone settings. Science of The Total Environment [Internet]. 2022 Aug 20 [cited 2025 Jul 22];835:155481. Available from: https://www.sciencedirect.com/science/article/abs/pii/S0048969722025773

19. Othman F, Sharip SM, Kasim S, Md A, Farrah K&, Baharuddin N. Flooding: The Mental, Social and Health Effects of Flood Disasters. 2022 [cited 2025 Jul 22]; Available from: http://dx.doi.org/10.6007/IJARBSS/v12-i10/15492

20. World Bank. Ghana: Volta River Authority (VRA); Appraisal of the Kpong Hydroelectric Project [Internet]. World Bank. 1977 Mar [cited 2025 Jul 22]. Available from: https://documents1.worldbank.org/curated/en/571301468282896201/pdf/multi-page.pdf

21. Natural Heritage Institute, Water Resources Commission (Ghana), Volta River Authority, Partners. Project Summary: Reoptimization and Reoperation Study of Akosombo and Kpong Dams. 2014 Dec.

22. Volta River Authority. Volta River Authority. 2025 [cited 2025 Jul 22]. Volta River Authority | Kpong Hydro Plant. Available from: https://www.vra.com/our_mandate/kpong_hydro_plant.php

23. African Development Fund. Ghana; Kpong Irrigation Project. 2005.

24. FSRP Ghana. Kpong Irrigation Scheme [Internet]. 2025 Apr [cited 2025 Jul 22]. Available from: https://fsrp.org.gh/blog/Kpong-Irrigation-Scheme

25. Dadzie H. GBC Ghana Online. 2023 [cited 2025 Jul 22]. Water supply to communities affected by Spillage of Akosombo and Kpong Dams will not be contaminated by debris- GWCL. Available from: https://www.gbcghanaonline.com/general/water-supply/2023/

26. Yirenya-Tawiah DR, Rashid AA, Futagbi G, Aboagye I, Dade M. Prevalence of snail vectors of schistosomiasis in the kpong head pond, Ghana. West African Journal of

Applied Ecology [Internet]. 2011 [cited 2025 Jul 22];18:40–5. Available from: https://pure.ug.edu.gh/en/publications/prevalence-of-snail-vectors-of-schistosomiasis-in-the-kpong-head--3

27. Zakhary K. Factors Affecting the Prevalence of Schistosomiasis in the Volta Region of Ghana. Vol. 3, MJM. 1997.

28. Opare J, Hervie T, Mensah E, Brown-Davies C, Asiedu O, Alomatu B, et al. Schistosomiasis in Ghana from baseline to now: the impact of fifteen years of interventions | Enhanced Reader. Front Public Health. 2025 Jun 6;13.

29. Volta River Authority. Press Release: Controlled Spillage of Water from the Akosombo Dam and Kpong Dam [Internet]. Accra; 2023 Sep [cited 2025 Jul 16]. Available from: https://www.vra.com/media/2023/Controlled-Spillage-from-the-Akosombo-Dam-and-Kpong-Dam.php

30. Smallwood T, Lefebvre V, Eyre R, Harrison J, Delaporte S, Attandoh R, et al. Ghana, Akosombo floods: Long- and medium-distance displaced subscribers from flood-affected areas of the Lower Volta River: Situation as of 22 October 2023 [Internet]. 2023 [cited 2024 Jan 31]. Available from: https://www.flowminder.org/resources/publications-reports/ghana-lower-volta-river-akosombo-floods-october-2023#

31. Volta River Authority. Volta River Authority | News - Controlled Spillage of Water from the Akosombo Dam and Kpong Dam [Internet]. Corporate Affairs & External Relations Unit V, editor. www.vra.com. Accra: Volta River Authority; 2023 [cited 2024 Jan 30]. Available from: https://www.vra.com/media/2023/Controlled-Spillage-from-the-Akosombo-Dam-and-Kpong-Dam.php

32. Modern Ghana. Akosombo Dam spillage: Everyone downstream will die if the dam overflows – Former VRA CEO rubbishes any blame. Modern Ghana [Internet]. 2025 May 21 [cited 2025 Jul 22]; Available from: https://www.modernghana.com/news/1402101/akosombo-dam-spillage-everyone-downstream-will.html

33. Arku SK. All you need to know about Ghana’s Akosombo Dam spillage : Peoples Dispatch. Peoples Dispatch [Internet]. 2023 Nov 1 [cited 2025 Jul 22]; Available from:

https://peoplesdispatch.org/2023/11/01/all-you-need-to-know-about-ghanas-akosombo-dam-spillage/

34. Ohio Environmental Protection Agency. Appendix IV-Inland Lakes Manual. In: Surface Water Field Sampling Manual. 2025.

35. Dzodzomenyo M, Asamoah M, Li C, Kichana E, Wright J. Impact of flooding on microbiological contamination of domestic water sources: a longitudinal study in northern Ghana. Appl Water Sci. 2022 Oct 1;12(10).

36. Ting ASY, Zoqratt MZHM, Tan HS, Hermawan AA, Talei A, Khu ST. Bacterial and eukaryotic microbial communities in urban water systems profiled via Illumina MiSeq platform. 3 Biotech [Internet]. 2021 Feb 1 [cited 2026 Jan 12];11(2):40. Available from: https://pmc.ncbi.nlm.nih.gov/articles/PMC7794265/

37. Zheng F, Zhang T, Yin S, Qin G, Chen J, Zhang J, et al. Comparison and interpretation of freshwater bacterial structure and interactions with organic to nutrient imbalances in restored wetlands. Front Microbiol [Internet]. 2022 Sep 21 [cited 2026 Jan 12];13:946537. Available from: https://pmc.ncbi.nlm.nih.gov/articles/PMC9533089/

38. Rahman T, Haider SMA, Khan FTZ, Islam MS, Mahmud ZH, Karim MM, et al. Flood-Driven Microbial Resurgence: Functional Shifts and Beneficial Taxa in Post-Flood Agricultural and Residential Soils in Bangladesh. Microbiologyopen [Internet]. 2025 Oct 1 [cited 2026 Jan 12];14(5):e70069. Available from: https://onlinelibrary.wiley.com/doi/full/10.1002/mbo3.70069

39. Zeglin LH. Stream microbial diversity in response to environmental changes: Review and synthesis of existing research. Front Microbiol [Internet]. 2015 May 18 [cited 2026 Jan 12];6(MAY):128252. Available from: www.frontiersin.org

40. Duze ST, Mkhize L, Marimani M, Patel M. A river in crisis: water quality, microbial burden, and public health implications of a South African urban river. Appl Environ Microbiol [Internet]. 2025 Nov 1 [cited 2026 Jan 12];91(11):e01566-25. Available from: https://pmc.ncbi.nlm.nih.gov/articles/PMC12628798/

41. Xiang H, Yang CG, Xiong HQ, Bao HR, Qu JZ, Luan ZX, et al. Spatial heterogeneity of microbial community structure and its environmental drivers in surface sediments of Erhai Lake. PLoS One [Internet]. 2025 Sep 1 [cited 2026 Jan 16];20(9):e0322915. Available from: https://pmc.ncbi.nlm.nih.gov/articles/PMC12410724/

42. Chen H, Zhang L, Zheng Z, Gao Y, Zhao Y. Hydrological connectivity shape the nitrogen pollution sources and microbial community structure in a river-lake connected system. Front Microbiol [Internet]. 2025 [cited 2026 Jan 16];16:1563578. Available from: https://pmc.ncbi.nlm.nih.gov/articles/PMC12023755/
43. Tan B, De Vera P, Abrazaldo J, Ng C. Flood-associated disease outbreaks and transmission in Southeast Asia. Front Microbiol [Internet]. 2025 [cited 2026 Jan 12];16:1694246. Available from: https://pmc.ncbi.nlm.nih.gov/articles/PMC12585969/
44. Yazdi MS, Ardalan MA, Hosseini M, Zoshk MY, Hami Z, Heidari R, et al. Infectious Diarrhea Risks as a Public Health Emergency in Floods; a Systematic Review and Meta-Analysis. Arch Acad Emerg Med [Internet]. 2024 [cited 2026 Jan 12];12(1):e46. Available from: https://pmc.ncbi.nlm.nih.gov/articles/PMC11221827/
45. Hayward C, Ross KE, Brown MH, Bentham R, Whiley H. The Presence of Opportunistic Premise Plumbing Pathogens in Residential Buildings: A Literature Review. Water (Switzerland) [Internet]. 2022 Apr 1 [cited 2026 Jan 12];14(7):1129. Available from: https://www.mdpi.com/2073-4441/14/7/1129/htm
46. Krishnakumar A, Jose J, Kaliraj S, Aditya SK, Krishnan KA. Assessment of the impact of flood on groundwater hydrochemistry and its suitability for drinking and irrigation in the River Periyar Lower Basin, India. Environ Sci Pollut Res Int [Internet]. 2022 Apr 1 [cited 2026 Jan 12];29(19):28267–306. Available from: https://pubmed.ncbi.nlm.nih.gov/34988810/
47. Peres R. Quantitative Microbial Risk Assessment in Rural and Remote Communities: A Critical Review. IOSR Journal of Environmental Science [Internet]. 2025 [cited 2026 Jan 29];19:67–76. Available from: www.iosrjournals.orgwww.iosrjournals.org
48. World Health Organization. Quantitative Microbial Risk Assessment: Application for Water Safety Management [Internet]. Geneva; 2016 [cited 2026 Jan 12]. Available from: http://www.who.
49. Watkin S, Ciric L, Kiernan M, Cloutman-Green E. A proposed classification system for opportunistic pathogens for improved healthcare infection prevention and control risk assessments.

50. Gerba CP. Environmentally Transmitted Pathogens. Environmental Microbiology: Third Edition. 2015;509–50.

51. Denissen J, Reyneke B, Waso-Reyneke M, Havenga B, Barnard T, Khan S, et al. Prevalence of ESKAPE pathogens in the environment: Antibiotic resistance status, community-acquired infection and risk to human health. Int J Hyg Environ Health. 2022 Jul 1;244:114006.

Fig 1

Click here to access/download;Figure;Fig 1.tiff

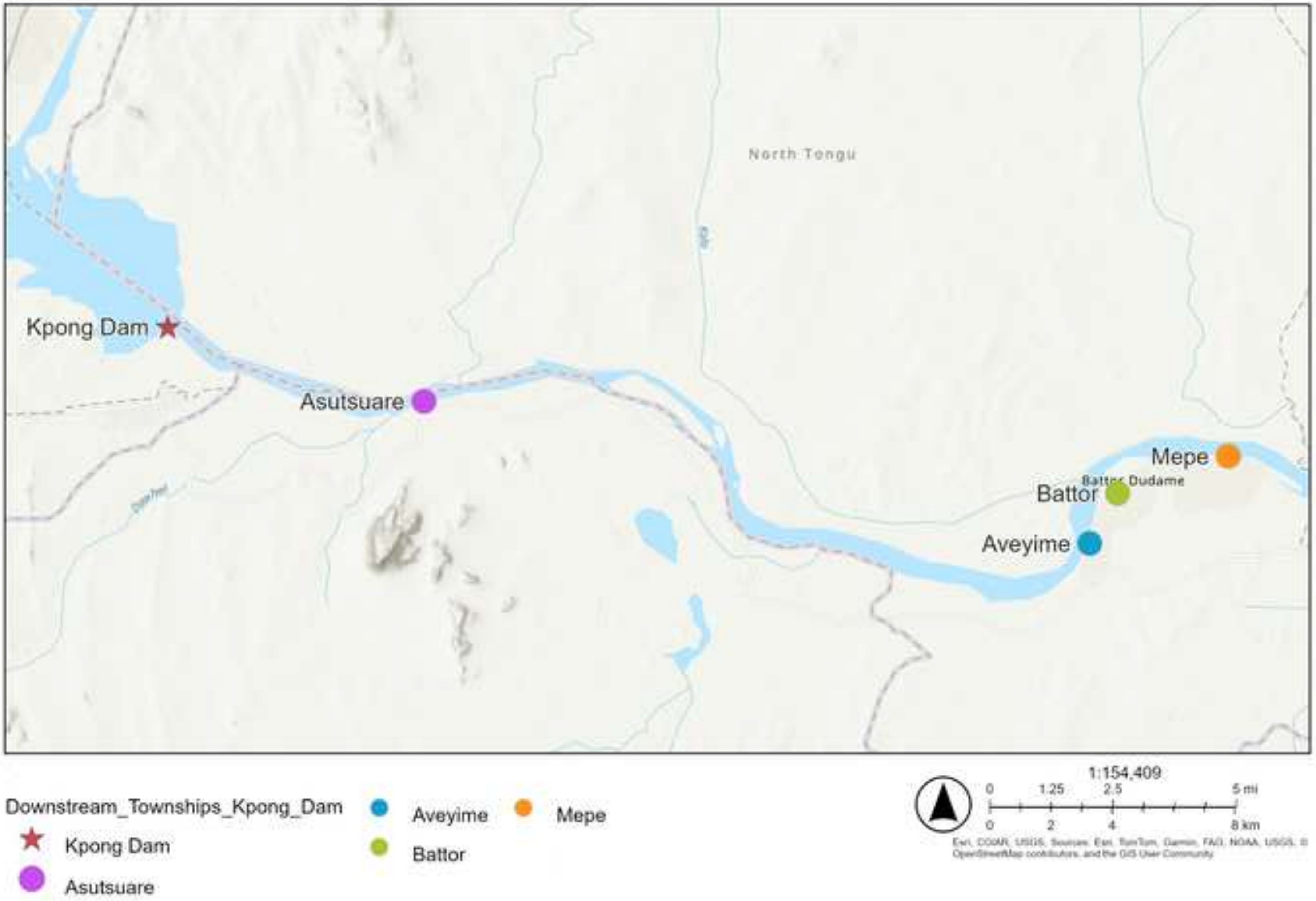

Fig 2

Click here to access/download;Figure;Fig 2.tiff

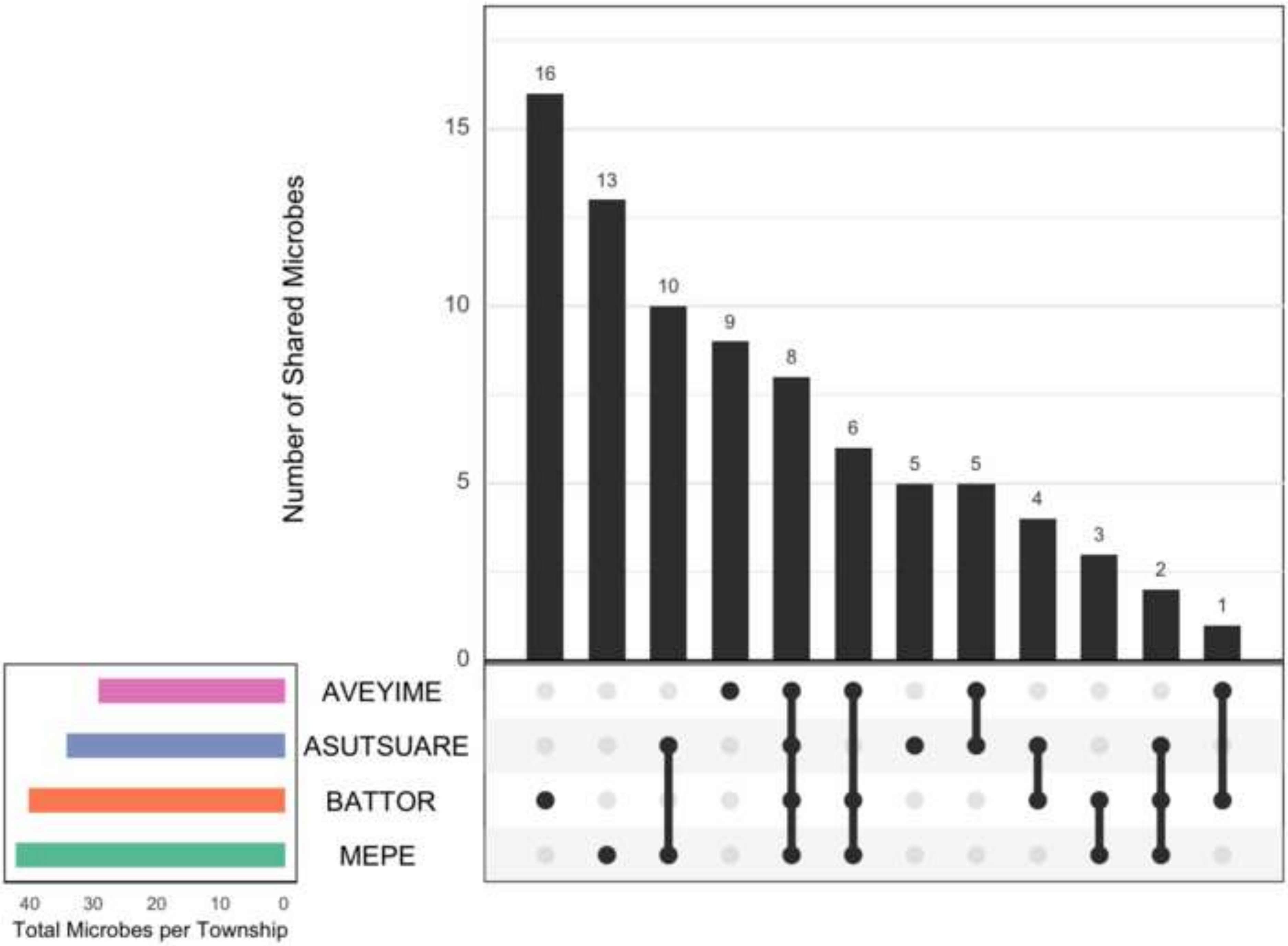

Fig 3

Click here to access/download;Figure;Fig 3.tiff

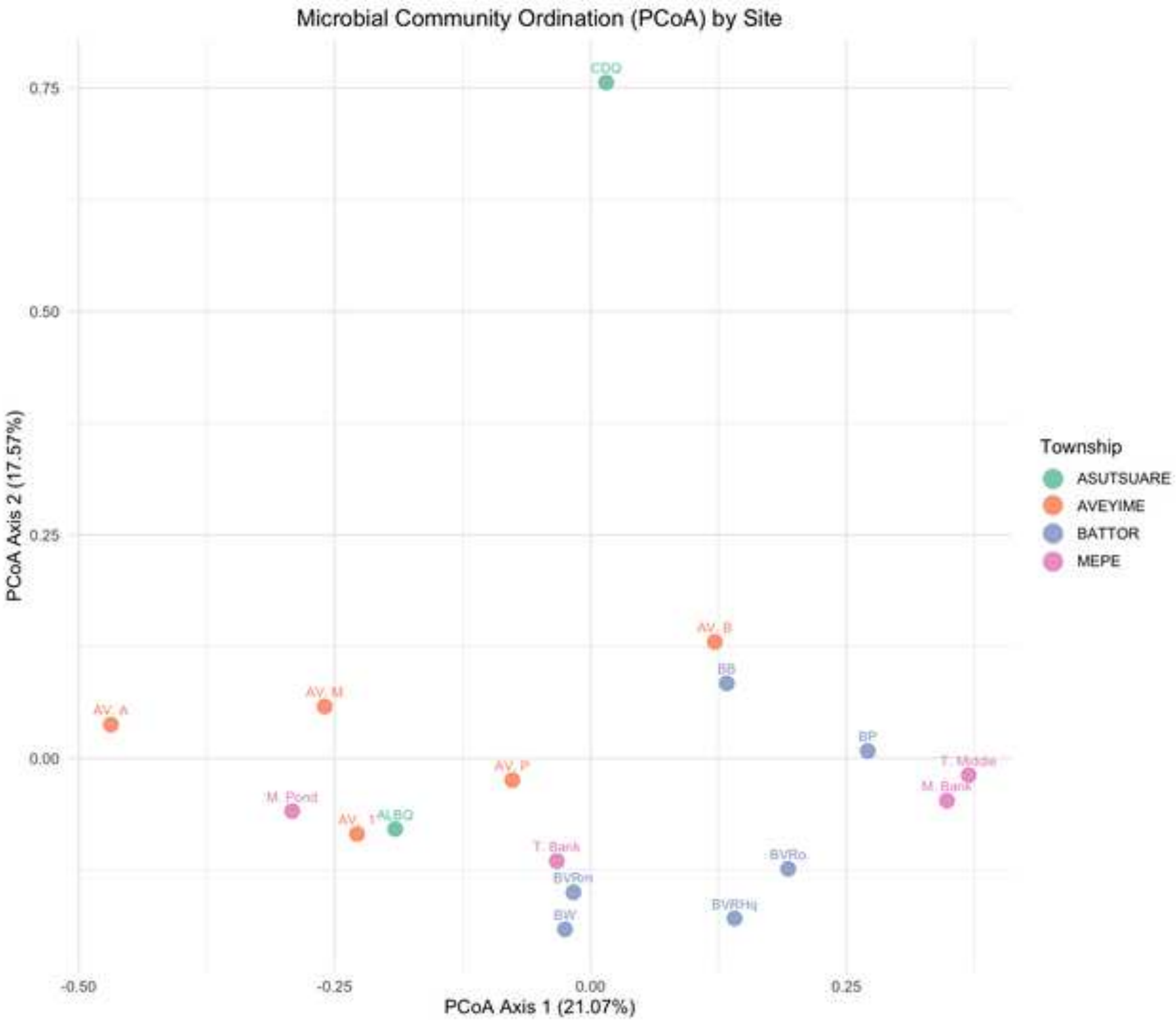

Fig 4

Click here to access/download;Figure;Fig 4.tiff

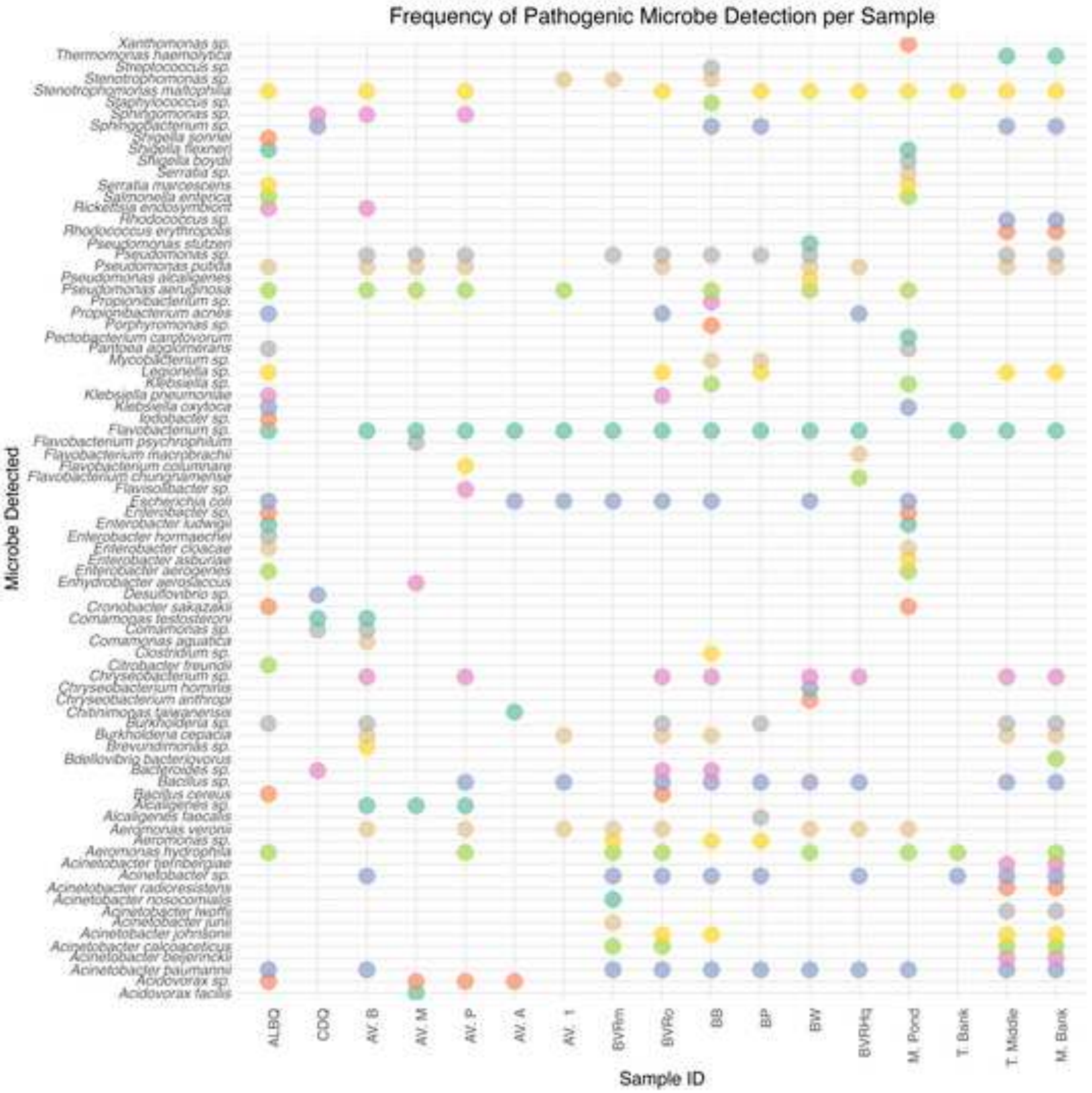

Supplement: Attachment — Submitted filename: PONE-D-26-14434_reviewer1.pdf [file pone.0346766.s005.pdf]
