# Supplementary material for: Downstream community risks post dam-spillage flooding along the Volta River in Ghana: potential pathogens and public health implications
Source: PLoS One. 2026 Jul 2;21(7):e0346766. doi: 10.1371/journal.pone.0346766 (PMC13327315; doi:10.1371/journal.pone.0346766)
Supplement: Attachment — Submitted filename: Response to Reviewers.docx [file pone.0346766.s007.docx]

**Response to Reviewers**

We appreciate the Editor and reviewer's comments and address each point below, with their enquiries or issues italized, and our responses provided underneath. All changes are highlighted with within the manuscript.

**Editor’s comments**

1. *If applicable, we recommend that you deposit your laboratory protocols in protocols.io to enhance the reproducibility of your results.*

**Response:** We thank the editor for this recommendation. All laboratory and analytical protocols used in this study have been deposited on protocols.io and are publicly accessible via the following DOIs:

1. Environmental Water Sample Collection Following Flooding. <https://dx.doi.org/10.17504/protocols.io.4r3l2dd64g1y/v2>
2. Vacuum Filtration and DNA Extraction from Environmental Water Samples. <https://dx.doi.org/10.17504/protocols.io.kqdg3mmkpl25/v2>
3. 16S rRNA Amplicon Sequencing Using Ion GeneStudio S5. <https://dx.doi.org/10.17504/protocols.io.5jyl84468g2w/v1>
4. Bioinformatics and Ecological Risk Classification of Environmental Water Microbiomes. <https://dx.doi.org/10.17504/protocols.io.rm7vz44o2lx1/v1>

**Lines 166-167, 177-178, 195-196, 215-217**

2. *Please provide additional information regarding the permits obtained for the work, including the full name of the authority that approved field site access, or a brief statement explaining why no permits were required.*

**Response:** We thank the editor for this comment. We have added a statement to the Methods section clarifying the ethical and regulatory basis for the study. All study procedures were conducted under ethical clearance granted by the Research Ethics Committee of the University of Health and Allied Sciences (UHAS), Ho, which covered all aspects of the work including field site access and community sample collection. Approval was granted under four protocol identification numbers corresponding to each study community: Asutsuare (UHAS-REC A.10 [162] 23-24), Aveyime (UHAS-REC A.10 [245] 23-24), Battor (UHAS-REC A.10 [248] 23-24), and Mepe (UHAS-REC A.10 [175] 23-24). No additional permits were required for field site access, as sampling was conducted from publicly accessible water bodies along the Volta River downstream of the Kpong Dam. This information has been incorporated into the Methods section of the revised manuscript.

**Lines 130-138**

3. *Please note that PLOS One has specific guidelines on code sharing for submissions in which author-generated code underpins the findings in the manuscript.*

**Response:** We thank the editor for this note. No author-generated code was produced in the course of this study. All analyses were conducted using standard software packages as described in the Methods section. As such, no custom code requires sharing or deposition.

4**.** *Please confirm whether your submission contains all raw data required to replicate the results of your study.*

**Response:** We thank the editor for this comment. We confirm that the minimal dataset required to replicate the findings of this study is available as follows:

- **Raw sequencing data:** The 16S rRNA gene sequencing reads generated in this study have been deposited in the NCBI Sequence Read Archive (SRA) under BioProject accession number PRJNA1469279.
- **Data underlying figures:** The data underlying all figures, including the UpSet plot and PCoA plot, have been included as additional Supporting Information files accompanying this revised submission as follows.

**S1 Fig. Microbial diversity, abundance and community composition charts.** Graphical summaries of microbial relative abundance and community composition across sampling sites, including Krona plots illustrating the taxonomic distribution of microbial communities detected in each sample.

**S2 Fig. Alpha diversity indices and plots.** Tabulated alpha diversity indices and associated graphical representations of within-sample microbial diversity across all sampling sites and townships.

**S1 Table. GPS coordinates of all 22 sampling sites.** Geographic coordinates recorded for each sampling site across the four study townships (Asutsuare, Aveyime, Battor, and Mepe) using a handheld GPS device.

- **S2 Table. Microbial risk classification data.** Tabulated data detailing the township, sampling site, identified microorganism, risk level, ecological category, group classification, and associated disease for all microbial taxa detected across sampling sites.

5. *Please amend your list of authors on the manuscript to ensure that each author is linked to an affiliation.*

**Response:** We thank the editor for this comment. We have reviewed the author list and confirmed that all authors, with the exception of Prince Agyirey-Kwakye, are affiliated with the University of Health and Allied Sciences (UHAS), Ho, Ghana (affiliation 1 and 3). Prince Agyirey-Kwakye is affiliated with the Department of Clinical Genomics, AttoDiagnostics Ltd, Attogroup, Norwich, Norfolk, United Kingdom (affiliation 2). The title page has been amended to ensure all authors are correctly linked to their respective affiliations.

6. *Figure 1 contains map/satellite images which may be copyrighted. Please either obtain written permission from the copyright holder to publish under CC BY 4.0, or remove the figure.*

**Response:** We thank the editor for drawing our attention to this. Figure 1 has been removed from the revised manuscript, and the other figures have been updated accordingly to reflect changes in figure numbers.

Fig 1 deleted

Fig 2 updated to Fig 1

Fig 3 updated to Fig 2

Fig 4 updated to Fig 3

7. *If the reviewer comments include a recommendation to cite specific previously published works, please review and evaluate these publications to determine whether they are relevant and should be cited.*

**Response:** No specific citations were recommended by the reviewers in this submission.

8. *Please review your reference list to ensure that it is complete and correct. If you have cited papers that have been retracted, please include the rationale for doing so in the manuscript text, or remove these references and replace them with relevant current references.*

**Response:** We thank the editor for this prompt. We have thoroughly reviewed the reference list and made the following corrections:

- One reference (previously reference 16, Kimberlin et al.) was removed as it was included in error and was unrelated to the subject matter of this study.
- Two duplicate references were identified and removed: Talbot et al. (2018) and Dzodzomenyo et al. (2022), with single entries retained in the appropriate citation locations.
- Author names for reference 2 (Kamalanandhini et al.) and reference 15 (Othman et al.) were corrected.
- The URL for reference 44 (World Health Organization) was corrected to the full and accurate link.
- Reference 43 (Rodrigues PM) was updated with the correct journal name and author details.
- New references were added to support statements in the text where additional citation was needed.
- Four protocols.io references (references 28–31) were added corresponding to the laboratory and analytical protocols deposited for this study.
- No retracted articles were identified in the reference list.

The revised reference list is included in the updated manuscript.

**Reviewer’s Comments**

1. *All scientific names of bacteria should be written according to standard format. For example in Table 1, all the initial "sp." for the species of the bacteria should not be italicized. In abstract, the scientific names of bacteria should be italicized.*

**Response:** We thank the reviewer for this observation. We have reviewed and corrected the formatting of all bacterial scientific names throughout the manuscript. Specifically, all instances of "sp." in the tables and manuscript have been changed to non-italicized format in accordance with standard microbiological nomenclature. Additionally, all bacterial scientific names in the abstract have been confirmed and corrected to italicized format as required. These corrections have been applied consistently throughout the revised manuscript.

2. *The flood water was occurring in September 2023 but the samples were taken in July 2024. How do you relate this with the water from the dam?*

**Response:** We thank the reviewer for raising this important point. We have added a clarifying statement to the Methods section to address this. Briefly, an initial sampling round was conducted once floodwaters had receded and it was safe to access the communities, however DNA quality from those initial samples was insufficient for sequencing. A second sampling round was therefore undertaken in July 2024 to obtain samples of adequate quality. Importantly, this extended post-flood window also allowed assessment of the persistence of microbial contamination well after the acute flooding phase, which we consider an important and novel aspect of this study. The relevant text has been added to the Methods section of the revised manuscript.

**Lines 123-130**

3. *Are there GPS coordinates for the sampling locations in the study?*

**Response:** We thank the reviewer for this comment. GPS coordinates for all 22 sampling locations are provided in Supporting Information Table S1, which accompanies this submission. We have also ensured that this is clearly referenced in the Sampling Design and Sample Collection subsection of the Methods section, where we state that the exact locations of each sampling site were geo-referenced using a handheld GPS device and that coordinates for all sampling sites are provided as supporting information S1 Table.

**Lines 149-150**

4. *This study would be more interesting if the author could include and relate relevant land use to the sampling locations. In addition, relating the land use to the microbial communities and health risk.*

**Response:** We thank the reviewer for this valuable suggestion. We agree that integrating land use data would provide additional context and further enrich the findings. However, as land use analysis was not an objective of the current study, this falls outside the scope of the present manuscript. We have nonetheless noted this as a recommended direction for future investigations in the revised manuscript, as we agree it would be a valuable addition to future work in these communities.
